# Recent Advances in Synthetic Strategies and Biological Properties of Indazole Scaffolds: A Review

**DOI:** 10.1007/s41061-025-00509-9

**Published:** 2025-07-01

**Authors:** S. N. Murthy Boddapati, Bhuvaneswari Chalapaka, Abraham Emmanuel Kola, Sreekantha Babu Jonnalagadda

**Affiliations:** 1 School of Chemistry & Physics, College of Agriculture, Engineering & Science, University of KwaZulu-Natal, Westville Campus, P Bag X 54001, Durban, 4000 South Africa; 2 Department of Chemistry, Sir C R Reddy College, P G Courses, Eluru, A.P. 534007 India; 3 Department of Chemistry, Adikavi Nannaya University, Rajamahendravaram, A.P. India; 4 Department of Chemistry, Y.V.N.R Government Degree College, Kaikaluru, A.P. India; 5 Freshmen Engneering Department, Ramachandra College of Engineering, Eluru, A.P. 534007 India

**Keywords:** Indazole, Heterocycles, Biological activities, Clinical trails, Synthetic routes, Mechanistic insights

## Abstract

Research on heterocyclic compounds is an area of continuous focus, capturing the interest of both synthetic and natural product chemists. Indazoles are one of the rare heterocycles that are available in nature. Indazole and its derivatives are one of the most important classes of heterocycles in pharmacological molecules. The structurally different indazole motifs, with impressive bioactivity, have drawn increasing attention from medicinal chemists in recent years for the continuous development of novel drug moieties. Thus, knowledge of the biological activities and synthetic pathways of indazole scaffolds is essential to enhancing further developments in the number of indazole-based lead molecules. The goal of the present review is to highlight information on the biological properties of some existing indazole-based drugs and activities of novel bioactive indazole compounds in clinical trails, with specific attention to the most recent advances in various synthetic strategies towards indazole and its derivatives over the past 7 years (2017–2024). Moreover, we discuss the substrate tolerance and mechanistic insights for most of the summarized synthetic protocols.

**Graphical Abstract:**

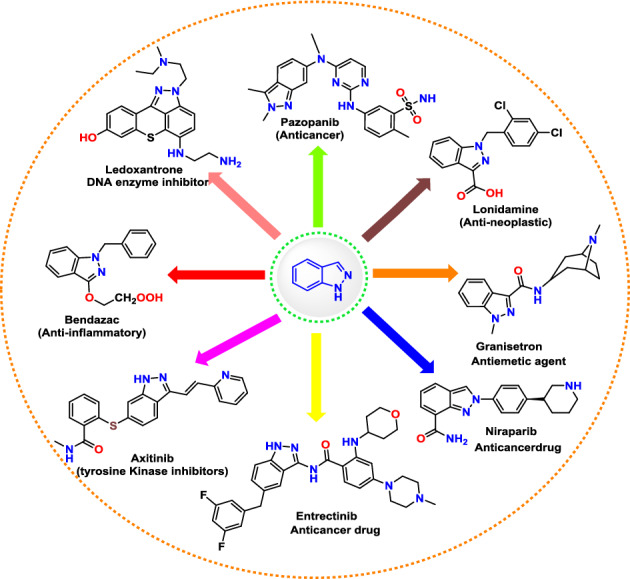

## Introduction

Most pharmacologically active scaffolds and a wide range of natural products constitute heterocyclic moieties. The diverse range of pharmacological and biological activities exhibited by *N*-containing heterocycles makes them highly significant [1]. Nitrogen heterocycles make up 70% of the most widely sold pharmaceuticals [2]. The family of *N*-containing heterocycles, especially the structural diversity of the indazole nucleus, has drawn immense attention in the past and present due to its broad biological potential.

**Fig. 1:**
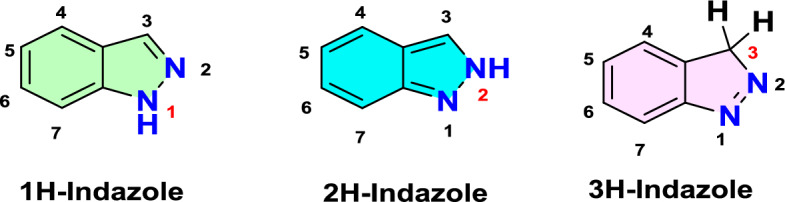
Tautomeric forms of indazoles

Indazole is an aromatic heterocyclic aromatic compound that has fused benzene and pyrazole rings. It is also known as benzpyrazole or isoindazone [3]. Two nitrogen atoms arranged in a five-membered ring make up the structure of indazole, which has ten π electrons. With two nitrogen atoms, the indazole ring displays annular tautomerism with respect to the location of the NH hydrogen atom. Because of the delocalization of π electrons, it can be seen in three tautomeric forms: 1*H*-indazole, 2*H*-indazole, and 3*H*-indazole, as shown in Fig. 1. Because of the energy difference between these tautomers, the 1*H*-tautomer (benzenoid form) exists in solid, liquid, and gaseous phases, and its derivatives are typically more thermodynamically stable than the consequent 2*H*- form (the quinonoid form). Thus, although it can tautomerize to 2*H*- and 3*H*-indazole forms, the indazole heterocycle is most often known as 1*H*-indazole (Fig. 1) [4]. Like the pyrazole molecule, indazole’s reactivity reflects a dual nature [5] that shows similarities to both pyrrole and pyridine. Indazole is amphoteric, which on deprotonation gives an indazolate anion and, on protonation, forms an indazolium cation. The subsequent *pK*_*a*_ values are 13.86 for the equilibrium between the indazolate anion and indazole and 1.04 for the equilibrium between indazole and the indazolium cation [6]. The planar nature of the heterocyclic ring of indazole allows a wide range of derivatives via positional functionalization and side chain replacements of varying lengths. The indazole nucleus is a constituent in a majority of agrochemicals, pharmaceuticals, dyes, and critical intermediates for drugs. They serve as essential starting materials in the synthesis of various drug-like molecules.

## Pharmacological Importance of Indazoles

Indazole is a powerful heterocyclic compound with a broad range of biological applications. This rarely available natural alkaloid is a typical ingredient in conventional medicine for curing a variety of disorders. In recent years, indazole motifs have grabbed the attention of medicinal chemists due to their utility as pharmacophores in drug discovery.

**Fig. 2:**
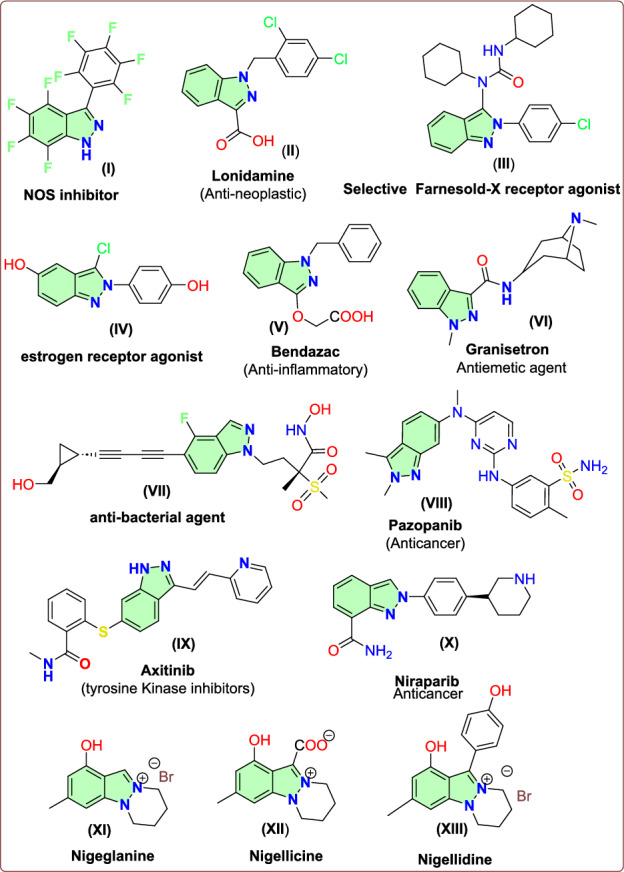
Significant bioactive indazole scaffolds

The broad spectrum of pharmacological and biological activity exhibited by indazole compounds makes them extremely important for a great number of scientists and researchers working in the field of drug discovery. There have been many publications, reviews, and over 400 patents or patent applications highlighting the exceptional biological activity associated with the indazole core [7–9]. A number of biological activities have been displayed by these compounds, including anti-cancer, anti-microbial, selective inhibitors of nitric oxide synthase (NOS), anti-inflammatory, and male contraceptive effects (Fig. 2).

The indazole motif is a very useful heterocyclic scaffold for creating new, physiologically active drugs. In particular, selective NOS-II inhibition shows excellent promise as a nitric oxide synthase (NOS) selective inhibitor (**I**, Fig. 2) [10] that aids in the production of nitric oxide from L-arginine. Additionally, indazole derivatives have applications as anti-neoplastic agents (**II**, Fig. 2), selective farnesoid-X receptor agonist (**III**, Fig. 2), and estrogen receptor agonist (**IV**, Fig. 2) [11–13]. One popular NSAID for treating musculoskeletal and joint pain is bendazac (**V**, Fig. 2), a nonsteroidal anti-inflammatory medication based on indazole [14]. Granisetron (**VI**, Fig. 2), an antagonist of the serotonin 5-HT3 receptor, is commonly used as an antiemetic [15]. Actelion Pharmaceuticals developed a number of 1*H*-indazole and analogues as potent anti-bacterial drugs with greater potency when compared to ciprofloxacin. Among these, compound **VII** in Fig. 2 was the most potent moiety with excellent minimum inhibitory concentration (MIC) efficacy against a range of bacterial strains [16]. Pazopanib (**VIII**, Fig. 2), also categorized as a tyrosine kinase inhibitor and marketed under the brand name Votrient, is used to treat advanced soft tissue carcinoma and renal cell carcinoma (RCC). Axitinib (**IX**, Fig. 1) is used to treat RCC [17, 18]. In the early 1900s, the compounds derived from the mustard family were utilized in cancer treatment. The initial members of the indazole family with similar properties include niraparib (**X**, Fig. 2), pazopanib, and axitinib [19–21]. All these potent drug motifs contain indazole. Indazoles are seldom found in nature, with only three compounds, nigeglanine (X**I**, Fig. 2) [22], nigellicine (**XII**, Fig. 2) [23, 24], and nigellidine (**XIII**, Fig. 1) [25], isolated from natural sources.

**Fig. 3:**
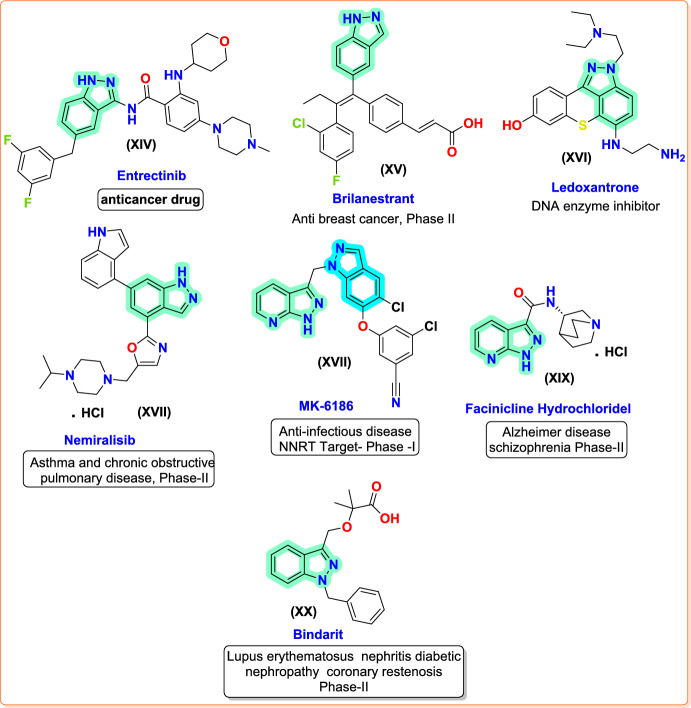
Bioactive indazole compounds in clinical trails

In recent years, many approved drugs and molecules under clinical investigation have possessed an indazole skeleton. More potent lead molecules or clinical drug moieties can be developed by modifying the active indazole derivatives according to structure–activity relationships. For instance, entrectinib (**XIV**, Fig. 3), the most current indazole-based medication approved for clinical use in 2019, was as of April 2021 marketed under the trade name Rozlytrek, used to treat certain cancer types in adults and children. It is a modern tropomyosin receptor kinase (TRK) inhibitor of silencing ROS1 and the anaplastic lymphoma kinase (ALK) repressor through central nervous system (CNS) activity, specifically aiming at solid tumors with NTRK1/2/3 (coding TRKA/TRKB/TRKC) fusion mutations of the ALK and ROS1 genes [26–28]. Brilanestrant (GDC-0810) (**XV**, Fig. 3) is an estrogen receptor (ER) inhibitor for breast cancer that is selective and orally accessible, and created by Johnson Roche; very good *in vitro* activity has been seen in its preliminary research and development [29–31]. The phase II trial (trial ID: NCT01823835) examining the combination of **XV** and palbociclib against breast cancer is still ongoing. Ledoxantrone’s (**XVI**, Fig. 3) phase II clinical trial was started 25 years ago to investigate its potentiality against colorectal cancer and metastatic colorectal cancer. However, at the prescribed schedule and dose, compound **XVI** is less effective against colorectal cancer [32, 33]. Clinical phase II studies were performed for hormone-refractory prostate cancer in 1999–2004, which demonstrated weak activity. GSK developed nemiralisib (X**VII**, Fig. 3), a potent and highly selective PI3Kδ inhibitor, primarily for chronic obstructive pulmonary disease (COPD) and asthma treatment [34–37]. Currently, a phase II clinical study is in progress against activated PI3Kδ syndrome (research ID: NCT02593539). Compound **XVII** was obtained by amending the C6 position of indazole, with an indole substituent at the C6 position, and it was regarded as the most effective [38, 39]. MK-6186 inhibitor (X**VIII**, Fig. 3) was an indazole-based compound investigated by Merck to treat HIV. With chloro-benzonitrile and duo indazole rings as constituents [40], it has demonstrated remarkable efficacy against both untamed and common viruses such as K103N and Y181C mutants with NNRTI resistance. Moreover, compound **XVIII** displayed suitable pharmacokinetic properties in dogs and rats, opening the door to a single human dosage [41]. Compound **XVIII** is a new non-nucleoside reverse transcriptase of the second generation. Facinicline hydrochloride (X**IX**, Fig. 3) is a potent *α*7-nicotinic acetylcholine receptor agonist that was found to improve sensorimotor and cognitive function in animal models [42–44]. It is used clinically to treat Alzheimer’s disease (AD)- and schizophrenia-related cognitive impairment. The phase II clinical trial of AD (study ID: NCT00454870) compound **XIX** was identified as an enhancer of episodic secondary memory quality and working memory scores [45].

Bindarit (X**X**, Fig. 3), with anti-inflammatory activity, is an indazole derivative that efficiently inhibits the human chemokine (C–C motif) ligand 2 (CCl_2_) transcriptions. It can ameliorate symptoms in animal models of numerous human diseases like adjuvant arthritis [46], autoimmune encephalitis [47], breast cancer [48], lupus nephritis [49], and severe acute pancreatitis [50]. The safety and effectiveness were assessed in phase II clinical trials (study ID: NCT01109212) on patients with lupus nephritis and diabetic nephropathy, which were completed in 2008. Based on the results, the drug was well tolerated and achieved a considerable decrease in albumin and urine CCl_2_ excretion [51].

Despite the outstanding advancements in indazole chemistry, there is a lack of the most up-to-date and concise reports on the preparation and biological applications of therapeutically significant indazole motifs compared to its bioisosteres such as indole or benzimidazole, probably due to the relatively low abundance of the indazole moieties in natural products. On the other hand, only a few reviews focused on these scaffolds. For example, Paidesetty et al. reviewed the chemistry and tautomeric effects of indazoles on the construction of indazole scaffolds, their biological properties, and future perspectives [52]. Congshan and coauthors [53] presented in their review the key aspects of investigating anticancer properties of indazoles, such as the structure–activity relationship (SAR), mechanisms of action, and structural modifications, and highlighted the significance and therapeutic potential of the indazole motifs as anticancer agents. Yin et al. [54] reviewed the antihypertensive activities and mechanism of action of various indazoles and their derivatives, along with an SAR study of the antihypertensive effects. Juvaley et al. [55] reviewed aspects of the importance of structural modifications in the therapeutic potential of indazole compounds towards the progression of anticancer agents. They described the SAR along with the compound’s structure with the respective half maximal inhibitory concentration (IC_50_) values against multiple human cancer cell lines and enzymes. Dehaen et al. [56] reported a number of atom-economical approaches via etal-promoted C−H functionalization and annulation processes to access structurally diverse indazoles. Archana’s group [57] published a detailed review of current advances in catalyst-based green strategies by spotlighting the transition metal, acid/base-catalyzed, and eco-friendly methods employed in the synthesis of indazole scaffolds. Park et al. provided a concise review [58] that provides a summary of synthetic strategies and functional utilization of 1*H*-indazole *N*-oxides holding significant potential in medicinal chemistry. Pan et al. summarized [59] modern developments in ultimate functionalization of 2*H*-indazoles. Hajra et al. [60] reported the progress of direct functionalizations of indazoles disclosed during the last 20 years.

Considering the plethora of applications of indazoles and their derivatives in numerous disciplines, we believe that the cumulation of synthetic approaches pertaining to these scaffolds will be very helpful for new researchers to concentrate on the construction and applications of indazole scaffolds. In continuation of our previous efforts [61, 62], herein we present a comprehensive review of these medicinally essential indazole motifs. The goal of this review is to compile several advancements in the synthesis of indazole scaffolds during the past 7 years (2017–2024, to date), as well as specific biological properties of indazoles and their derivatives. The improvements in indazole synthesis, along with a concise synopsis of their biological properties, open up new avenues for next-generation researchers to work on indazole scaffolds to construct a variety of potent lead molecules.

## General Synthetic Methods of Indazole

The construction of indazole motifs has generated intense interest due to the potential use of these naturally occurring compounds in the treatment of different diseases. The diverse biological applications of indazole and its analogues have resulted in the attention of synthetic organic chemists globally, motivating them to devise innovative and effective methods for constructing pharmacologically noteworthy indazole scaffolds. A number of paths for synthesizing these compounds have been developed, such as metal-free, metal-promoted intramolecular C–N bond formation.

**Scheme 1:**
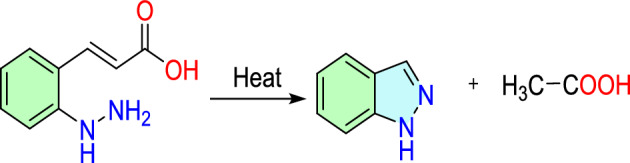
The first report of the creation of indazole

Early in the 1880s, Nobel laureate Emil Fisher created indazoles by synthesizing *o*-hydrazino cinnamic acid, as presented in Scheme 1 [63]. 1*H*-Indazole is produced by thermally cyclizing *o*-hydrazino cinnamic acid in this process. When he first attempted to create the anhydride of *o*-hydrazino cinnamic acid, he unexpectedly obtained a vast number of products. Among these, he discovered that one contained no oxygen, which was later identified as indazole.

**Scheme 2:**
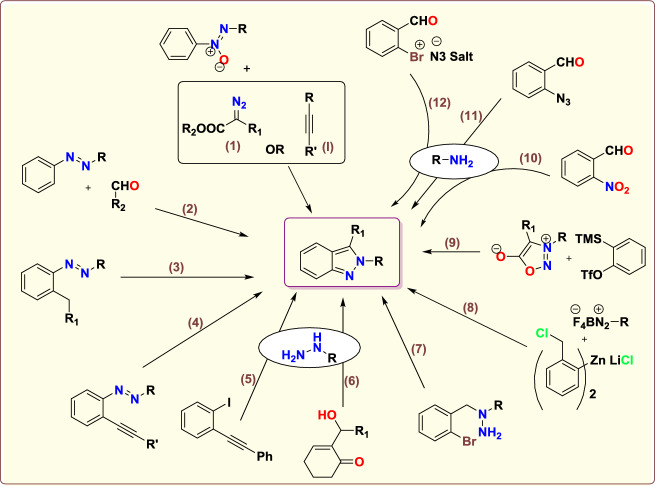
Previously reported techniques for the construction of indazoles [64–75]

There are numerous traditional synthetic routes reported to create 2*H*-indazoles (Scheme 2); For instance, indazoles can be obtained by tandem Rh(III)-mediated C−H alkylation plus intramolecular decarboxylative cyclization of azoxy motifs with diazo esters [64] or alkynes [65] (routes (**1**) and (**2**)); I_2_-catalyzed intramolecular oxidative annulation of *o*-alkyl azoarenes; through the reaction of 2-halophenyl acetylenes with aryl hydrazine(route **3**) [66]; and Pd-promoted intramolecular C–N bond making of *ortho*-alkyne azoarene (route **4**) [67].

Additional methods have used 2-nitro benzaldehyde and a primary amine via intramolecular reductive amination followed by *N*-hetero-cyclization [68, 69] through in situ imine creation by the condensation between 2-azido benzaldehyde with primary amine [70]; Cu-promoted condensation reaction of primary amine, 2-br-benzaldehyde, and azide (via three-component one-pot response) [71]; Pd/dppf-catalyzed intramolecular amination of *N*-aryl-*N*-(*o*-bromobenzyl)-hydrazines in the presence of base NaO^*t*^Bu [72]; from bis(2-chloromethylaryl) zinc reagents and aryldiazonium salt [73]; from arynes and sydnones via TBAF-promoted [3+2] dipolar cycloaddition [74]; and Baylis–Hillman adduct on cross-coupling reaction with aryl hydrazine [75]. Other efficient and recent synthetic protocols developed for the construction of indazoles are described here.

An alternative to the conventional synthetic protocols, C–H activation processes lead to transition metal-mediated cross-coupling reactions that have gained significance recently for the formation of carbon–carbon and carbon–nitrogen bonds [76–78]**.**

### Rh(III)-Catalyzed C–H Activation of Phthalazinones and Allenes

**Scheme 3:**
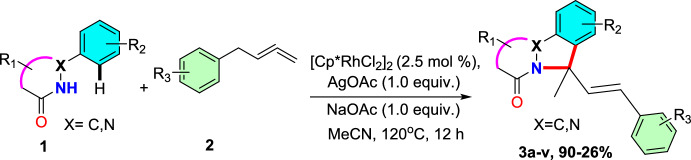
Synthesis of indazole scaffolds **3a**–**v**

Yin’s group described an efficient Rh(III)-promoted annulation of pyridazinones or phthalazinones by diverse allenes for the construction of indazole motifs with a quaternary carbon in moderate to good yields [79]. They created an effective C–H oxidative cyclization process using Rh(III) applicable to a range of allenes to produce *N*-aryl phthalazinones and pyridazinones. After successive C–H activation, olefin insertions after *β*-hydride removal, and intramolecular cyclization was performed in order to synthesize the intended products (Scheme 3). Under ideal reaction conditions, various 1-phenyl-1,2-dihydropyridazine-3,6-diones(**1**)reacted with buta-2,3-dien-1-ylbenzene(**2**) at 120 °C in an air-filled environment in the presence of 2.5 mol% [Cp*RhCl_2_]_2_ and 10 mol% AgOAc in MeCN for 12 h to obtain the respective target indazole derivatives (**3a**–**v**) in 26–88% yields. A broad substrate scope, good atom efficiency, and high Z-selectivity were all achieved with ease by this synthetic protocol. With this viable approach, a series of indazole derivatives with yields ranging from moderate to excellent were synthesized. This catalytic system displayed excellent atom economy and substrate tolerance.

**Scheme 4:**
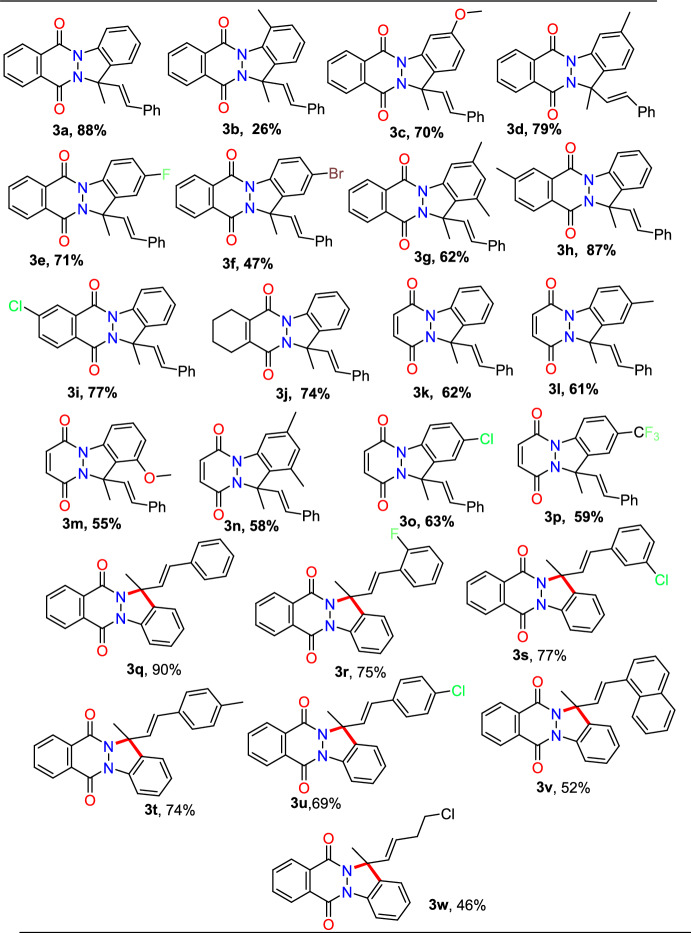
Substrate scope for the Rh(III)-promoted C–H activation of phthalazinones and allenes

Following that, the range of substrates for *N*-aryl phthalazinones and pyridazinones was investigated (Scheme 4). Initially, at ideal reaction conditions, model substrates 1-phenyl-1,2-dihydropyridazine-3,6-dione (**1a**) reacted with buta-2,3-dien-1-ylbenzene (**2**) in an air-filled environment to produce the anticipated indazole (**3a**) in 88% yield.

As indicated by Scheme 4, under standard reaction conditions, the substrates bearing different electron-donating groups (EDGs) or electron-withdrawing groups (EWGs) at various positions undergo the [4+1] cyclization process and produce the cyclic products in superior yields. Steric hindrance is responsible for the lower yield of the intended product **3b** obtained by the reaction of the substrate **1b** bearing an *ortho*-methyl unit. *N*-aryl phthalazinones with –OMe, –Me, –F, –Cl, and –Br groups afforded the consequent products (**3c–3f**) in 47–79% yields at optimized conditions. The 3,4-di Me-substituted substrate yielded the intended product **3g** in 62% yield. Moreover, the phthalazine moiety, with an electron-donating –CH_3_ group and electron-withdrawing –Cl group, afforded the required products (**3h** and **3i**) in 87% and 77% yields, respectively. Next, 1-phenyl-1,2-dihydropyridazine-3,6-dione was combined with buta-2,3-dien-1-ylbenzene to afford the indazole product **3k** in 62% yield. Moreover, 1-phenyl-1,2-dihydropyridazine-3,6-diones donating the −Me, −OMe, and −diMe substituents afforded the products **3l–n** in 55–61% yields. Similarly,1-phenyl-1,2-dihydropyridazine-3,6-diones having withdrawing −Cl and −CF_3_ groups gave the consecutive products **3o** and **3p** in 63% and 59% yields, respectively. Further, the adaptability of this Rh(III)-promoted [4+1] annulation process was assessed for diversely substituted allenes (Scheme 4). Allenes with 2-Me and 2–F substituents gave the intended products **3q** and **3r**in 90% and 75% yields, respectively. Allene with *meta* substitution such as 3–Cl reacted well to supply the equivalent product **3s** in 77% yield. Moreover, para-substituted allenes having 4–Me and 4–Cl substitutions gave the desired products **3t** and **3u** in 80% and 69% yields, respectively. Likewise, 1-naphthyl-substituted allenes afforded the cyclized product **3v** in 52% yield. Next, the aliphatic allene obtained the required compound **3w** in 46% yield, demonstrating that conjugated olefin as a substrate is not a force driving the catalytic system. Conversely, in this method, 1,1,4-trisubstituted and simple aliphatic allenes bearing an ester unit failed to afford the desired annulation products.

**Scheme 5:**
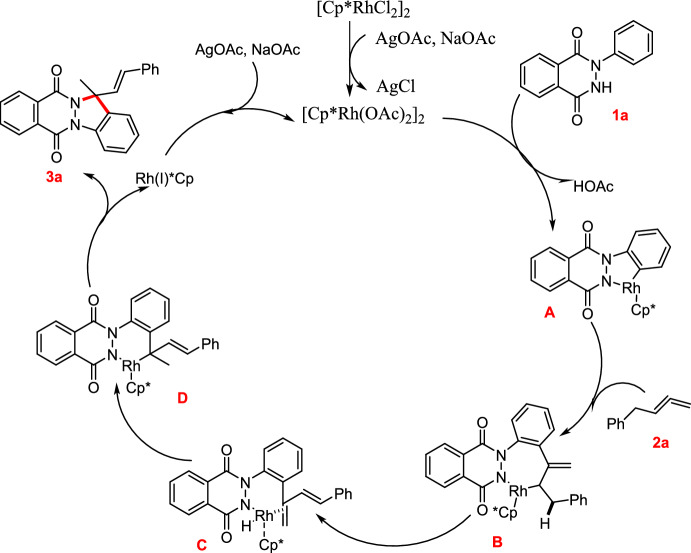
Possible mechanism for indazole **3a** formation

The probable mechanistic route for the reported Rh(III)-mediated [4+1] annulation of *N*-aryl phthalazinones and pyridazinones with allenes is described in Scheme 5. In order to form intermediate **A**, substrate, **1a** must first react with active rhodium, which is created in situ through ion exchange of [Cp*RhCl_2_]_2_ with silver acetate (AgOAc) and sodium acetate (NaOAc). Then, intermediate **B** is produced by allene **2a** insertion and subsequent removal of the β-hydride. Next, the Rh–H bond in intermediate **C** is included in the diene moiety to form **D**, which, on reductive elimination, gives product **3a**. Finally, the activated rhodium is regenerated by the oxidation of Rh(I) species obtained in a catalytic cycle with AgOAc and NaOAc.

### Rhodium-Catalyzed Synthesis of Substituted Indazole *N*-Oxides

Feng’s group described atom-economic, straightforward, and one-pot rhodium(III)-mediated C–H activation and cyclization protocol for the construction of valuable indazole *N*-oxides in modest to fine yields from commonly available *N*-nitrosoanilines (**4**) and sulfoxonium ylides (**5**) [80].

**Scheme 6:**
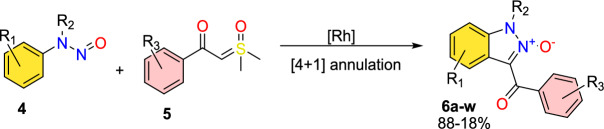
Synthesis of indazole *N*-oxides

In this method, nitroso is used as a traceless guiding group. Significantly, the reactivity pattern for the *N*-nitroso group is represented in this new annulation process, which has never been seen before. The protocol [80] features its prevailing reactivity, strong atom economy and good functional group tolerance under an ambient atmosphere in mild conditions (Scheme 6).

**Scheme 7:**
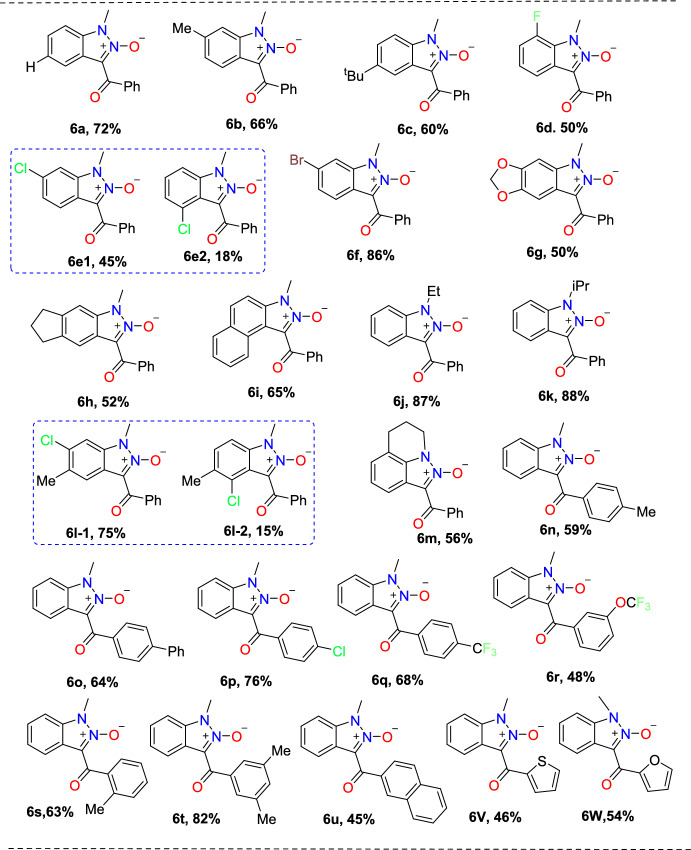
Scope of sulfonium ylides for the construction of indazole *N-*oxides

Under optimized reaction conditions, the scope of various *N-*nitrosoanilines (**4**) with **5a** as the coupling partner was explored (Scheme 7). The meta-substituted substrates (*m*-Me, *m*-Cl, *m*-Br) showed efficiency by providing the respective products **6b**, **6e**, and **6f** in moderate (86–45%) yields in 10 h. The *m-*Cl-substituted *N*-nitroso anilines produced a mixture of regioselective products isolated in the ratio of 2.5:1 (**6e-1**, **6e-2**). The reaction produced the consecutive product **6c** in superior yield using *para*-substituted *N-*nitrosoanilines with an electron-donating group (EDG) (*p*-^*t*^Bu). The *ortho*-substituted substrate *N*-(2-fluorophenyl)-*N*-methylnitrous amide produced the respective indazole product **6d** in 50% yield. Moreover, the tricky disubstituted *N*-methyl-*N*-nitrosoanilines showed their compatibility by affording the indazoles **6h** and **6i** in good yields. In addition, product **6j** was successfully obtained in 65% yield through the domino reaction of naphthalene scaffolds. The reaction went well for the *N*-ethyl and *N*-propyl substituents, yielding the products **6j** and **6k** in 87% and 88% yields, respectively. Furthermore, substituted *N*-nitroso-tetrahydroquinoline tolerated this reaction well, yielding **3at** and **3au** in 56 and 62% yields, respectively. Beyond that, the disubstituted *N-*nitrosoaniline with both –Cl and –Me substituents afforded a mixture of products **6l-1** and **6l-2** in a 5:1 ratio, giving 75% and 15% yields, respectively. Next, the reaction with *N*-nitroso-tetrahydroquinoline proceeded smoothly to afford the product **6m** in 56% yield.

Further, the researchers examined the reaction between an extensive range of sulfoxonium ylides and *N*-nitrosoanilines **1a**. The results revealed that aromatic sulfoxonium ylides with diverse electron-withdrawing and electron-donating groups at *para* or *meta* position exhibited excellent compatibility to form the respective indazole scaffolds (**6n–u**) in satisfactory to excellent yields. Benzoyl sulfoxonium ylide with *p*-Me, *p*-phenyl, *p*-Cl, and *p*-CF_3_ groups provided the products **6n–q** in 59–76% yields. Similarly, the benzoyl sulfoxonium ylides with *m*-OCF_3,_ *o*-Me and 3,5-diMe substituents afforded the corresponding products **6r–t** in 82–48% yields. Likewise, the naphthyl-based benzoyl sulfoxonium ylide also yielded the respective product **6u** in 45% yield. Further, heterocyclic substrates like furanyl and thienyl yielded the target products **6v** and **6w** in 46% and 54% yields, respectively.

**Scheme 8:**
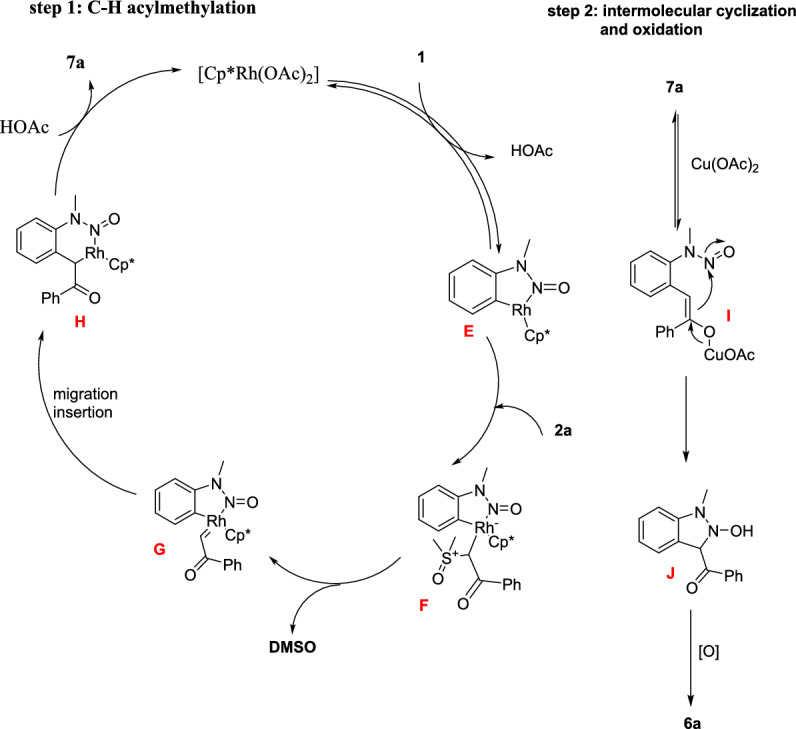
Possible mechanism for the formation of inazole *N*-oxide

Scheme 8 summarizes the possible mechanistic path. First, the *N*-nitrosoaniline (**4**) on cyclometalation gave the rhodacyclic intermediate **E**, which undergoes coordination with sulfoxonium ylide (**5**) to produce the Rh(III) intermediate **F**. The intermediate **F** on consequent α-elimination of dimethyl sulfoxide (DMSO) gave a reactive rhodium α-oxocarbene species **G** that further commenced migratory insertion of the Rh–C bond to construct a six-membered rhodacyclic intermediate **H**. The Rh–C and Rh–N bonds of intermediate **H** on protonolysis discharge the acylmethylated intermediate **7a** collectively by the active catalyst regeneration. After that, the Cu(OAc)_2_-promoted intramolecular cyclization affords the intermediate **I**, which on consequent oxidation either with Cu(II) or Ag_2_O additives, provides the final target product **6a**. Mechanistic investigations have shown that the C–H activation is reversible through a concerted metalation–deprotonation (CMD) mechanism.

### Rh(III)-Promoted Annulation of Azobenzenes with Vinylene

**Scheme 9:**
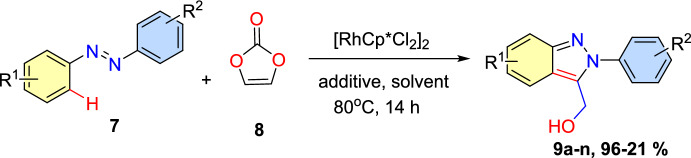
Construction of 2*H*-indazol-3-yl-methanolderivatives

Park’s group reported [81] rhodium(III)-mediated C−H functionalization and consequent intramolecular cyclization reaction between azobenzenes and vinylene carbonate to obtain 2*H*-indazoles (Scheme 9). Based on the azobenzenes’ electronic characteristics, this transformation produces *ortho*-alkylated azo-intermediates, which by decarboxylation forms (2*H*)-indazoles. Interestingly, in this reaction, the vinylene carbonate operates as an acetyl proximate or acetaldehyde to aid the [4+1] or [4+2] annulation reactions. The electron-rich azobenzenes on Ag^+^ additive supported [4+1] annulation, producing the respective C3-hydroxymethylated (2*H*)-indazoles. In contrast, the electron-poor azobenzenes exhibited comparatively low reactivity during the [4+2] annulation reaction. This distinguished method showed undesirable chemo- and regioselectivity, and wide functional-group tolerance in moderate reaction conditions.

**Scheme 10:**
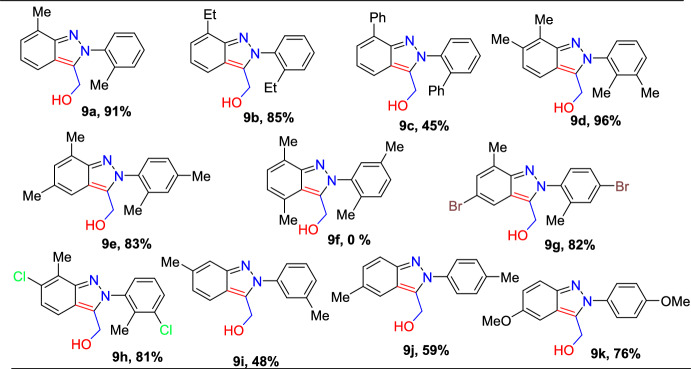
Substrate scope for the generation of 2*H*-indazol-3-yl-methanol derivatives

Next, the authors explored the scope of the azobenzenes **7a–q** towards the coupling reaction with vinylene carbonate (**8**) under standard conditions; Scheme 10 presents the results. Ortho-substituted azobenzenes **7a–c** having electron-rich groups (*o*-Me, *o*-Et, -*o*-Ph) produced the corresponding C3-hydroxymethylated (2*H*)-indazoles **9a**–**c** in moderate to high (45–91%) yields.

Azobenzenes with 2,3-diMe and 2,4-diMe substitutions coupled with vinylene carbonate gave the indazoles **9d** and **9e** in 96% and 83% yields, respectively. Conversely, the sterically crowded azobenzene **7f** with 2,5-dimethyl units did not form the desired product **9f**. Moreover, 2-methyl-4-bromo and 2-methyl-3-chloro azobenzenes tolerated the reaction well to afford the consecutive products **9g** and **9h** in 82% and 81% yields, respectively. The azobenzenes with electron-donating 3-Me, 4-Me, and 4-OMe groups produced the target indazoles **3n** and **3o** in 48% and 59% yields **9i–k** in 48–76% yields, respectively.

**Scheme 11:**
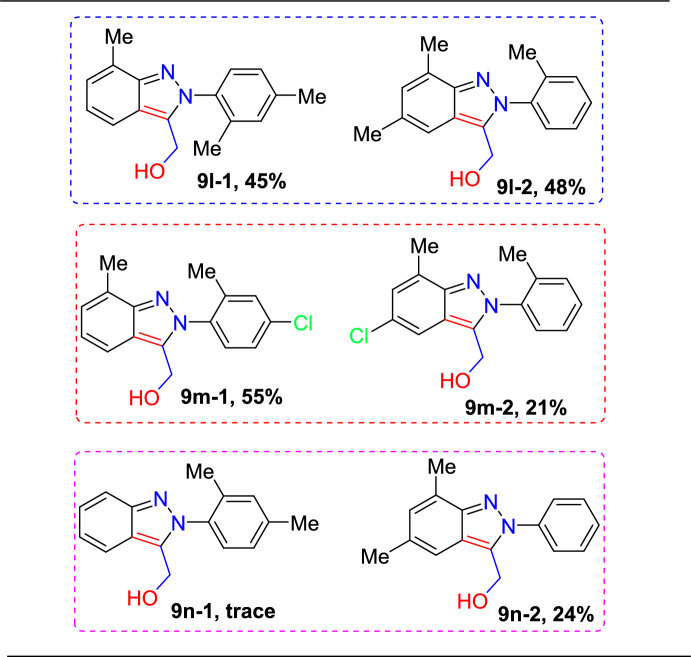
Substrate scope for unsymmetrical 2*H*-indazol-3-yl-methanol scaffolds

Next, under standard reaction conditions, the unsymmetrical azobenzenes **1l**–**n** also tolerated the reaction with vinylene carbonate (**8**) and afforded the target indazoles **9l**–**n** in moderate yields (Scheme 11). Unsymmetrical azobenzene having electron-rich –Me units on both aromatic rings (**7q**) produced an approximately equimolar quantity of **9l-1** and **9l-2** in a collective yield of 93%. The unsymmetrical azobenzene with a –Me group on one ring and −Me and –Cl groups on other ring and -diMe groups reacted with (**8**) to produce the corresponding (2*H*)-indazoles **9m-1** and **9n-2** as the major products, indicating that the transformation can primarily take place on a more electron-dense aromatic ring. These findings imply that the electronic environment of the azobenzene motif can regulate the site selectivity of this technique.

**Scheme 12:**
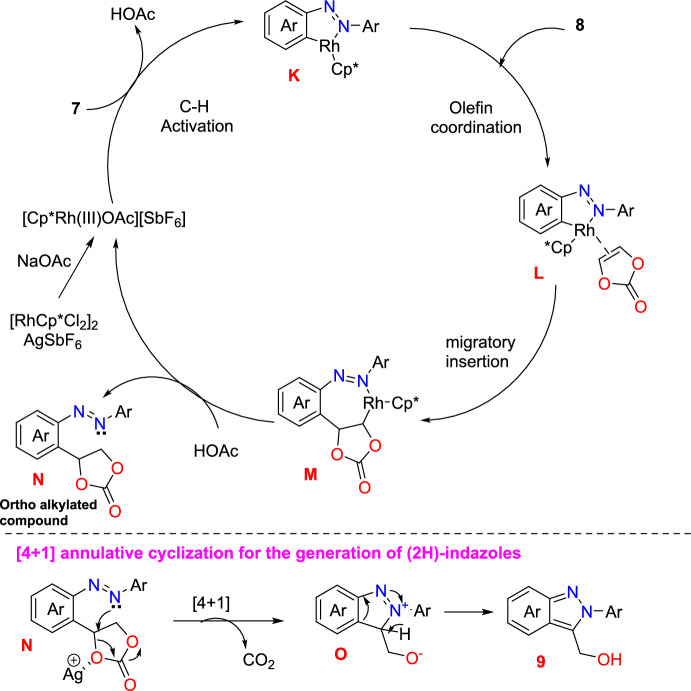
Plausible mechanism of 2*H*-indazol-3-yl-methanol synthesis

Scheme 12 illustrates the plausible mechanism predicted based on the experimental data and literature reports on the C−H functionalization of azobenzenes. In the presence of NaOAc, [RhCp*Cl_2_]_2_ and AgSbF_6_ create the cationic Rh(III) catalyst, which activates the azobenzene (**7**) *ortho*-C−H bonds to obtain the five-membered rhodacycle intermediate **K**. Next, the seven-membered rhodacycle species **M** is produced by the olefin coordination of the intermediate **K** with **8** followed by migratory insertion. The intermediate **M** then undergoes protonation, giving the *ortho*-alkylated product **N**. Next, the Ag^+^ additive, as a Lewis acid, facilitates the nucleophilic substitution of an azo group at the α- or β-position on the dioxolan-2-one moiety. An azo group can attack the benzylic (α) carbon in electron-rich azobenzenes to produce the [4+1] annulation intermediate **O**. This intermediate then undergoes aromatization to produce (2*H*)-indazole (**9**).

### Rh-Catalyzed Solvent-Dependent Synthesis via Multiple Bond Activations

**Scheme 13:**
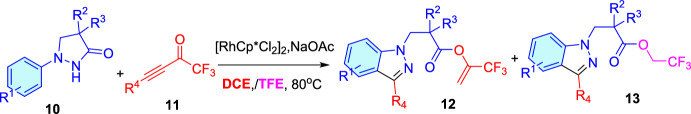
Synthesis of CF_3_-tethered indazoles

Fan et al. reported a solvent-dependent selective construction of CF_3_-tethered indazole scaffolds by the cascade reactions of 1-aryl pyrazolidinones (**10**) with trifluoromethyl ynones (**11**) carried out in DCE or TFE) as presented in Scheme 13 [82]. This was the first report on the construction of CF_3_-tethered indazole scaffolds via simultaneous alkynyl activation, pyrazole formation, and CF_3_ migration. This method has a few key features like redox-neutral conditions, wide substrate tolerance, excellent competence and compatibility with a range of functional groups, high atom economy, easily controllable selectivity, and the successful activation of multiple chemical bonds, including an alkynyl triple bond, and can be readily scaled up.

The generation of the target products involves the cascade N−H/C−H/C−N/C−C bond breakage with pyrazole ring development and pyrazolidinone ring opening. 1-Phenyl pyrazolidinone works as a C_2_N_2_ synthon, whereas the trifluoromethyl ynone serves up as a C_1_ synthon for the production of the pyrazole motif. In the interim, for pyrazolidinone ring opening, the trifluoromethyl ynone serves as an enol unit and affords a trifluoro propenoxy fragment through the breakage of the alkynyl triple bond and relocation of the broken moiety. Indazole motifs tethered with a trifluoroethoxy moiety were produced selectively through in situ transesterification using DCE as solvent instead of trifluoroethanol.

**Scheme 14:**
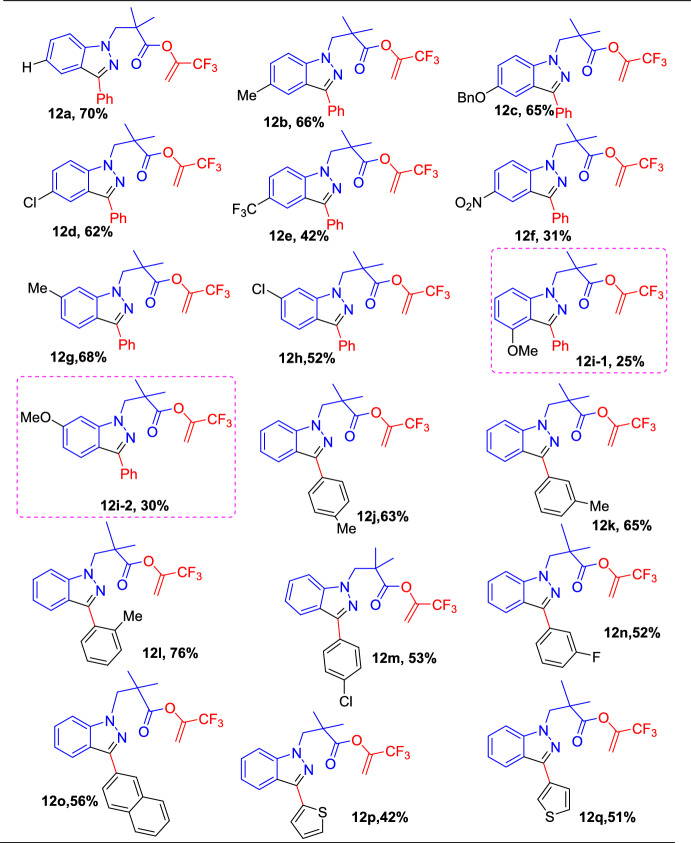
Substrate scope for preparation of CF_3_-tethered indazoles

Initially, the conditions for the reaction of 4,4-dimethyl-1-phenylpyrazolidin-3-one (**10**) with 1,1,1-trifluoro-4-phenylbut-3-yn-2-one (**11**) which gave the target indazoles 3,3,3-trifluoroprop-1-en-2-yl 2,2-dimethyl-3-(3-phenyl-1*H*-indazol-1-yl)propanoate (**12a**) and 2,2,2-trifluoroethyl 2,2-dimethyl-3-(3-phenyl-1*H*-indazol-1-yl)propanoate(**13a**) in good yields were optimized. The suitability of different substituted 1-arylpyrazolidinones (**10**) for the reaction with 1,1,1-trifluoro-4-phenylbut-3-yn-2-one (**11**) was examined, and Scheme 14 summarizes the obtained results. 1-Aryl pyrazolidinones bearing either an electron-donating group such as −Me or −OBn— and/or an electron-withdrawing group like −Cl, −CF_3_, and −NO_2_ on the *para*-position of the phenyl rings reacted with 1,1,1-trifluoro-4-phenylbut-3-yn-2-one (**11**) efficiently. It produced the respective CF_3_-indazole scaffolds **12b**–**e** in moderate yields. Moreover, 1-aryl pyrazolidinone with a −Me, −Cl, unit on the meta position of its 1-phenyl ring reacted with **11** quickly to give the target products **12g** and **12h** in 68% and 52% yields, respectively. Conspicuously, these meta-substituted substrates have a couple of unsymmetrical *ortho*-sites for C−H bond activation; interestingly, they prefer to react with **11** absolutely on the less sterically hindered side, indicating some steric effects on this reaction. 1-Arylpyrazolidinone (**1**) bearing a *m-*OMeC_6_H_4_ moiety produced a combination of two regio-isomers, **12i-1** and **12i-2,** in a 1:1.5 ratio with 25% and 30% yields, respectively.

Further, the scope of CF_3_-ynones (**12**) was explored and phenyl-substituted CF_3_-ynones with either a donating group like *p*-Me, *m*–Me, or *o*-Me units produced the target products **12j**–**12l** in 63–76% yields. The *ortho-*MePhenyl-substituted CF_3_-ynone gave a better yield compared to its meta and para isomers. Similarly, phenyl CF_3_-ynones with withdrawing groups like *p*-Cl and *m*-F on the phenyl ring reacted with 4,4-dimethyl-1-phenylpyrazolidin-3-one (**10a**) efficiently to produce the target products **12m** and **12n** in good yields. The naphthyl-, 2-thienyl-, 3-thienyl-, or ferrocenyl-group substituted CF_3_-ynone participated in this cascade reaction and afforded the products **12o**, **12p**, and **12q**, respectively, in moderate yields.

**Scheme 15:**
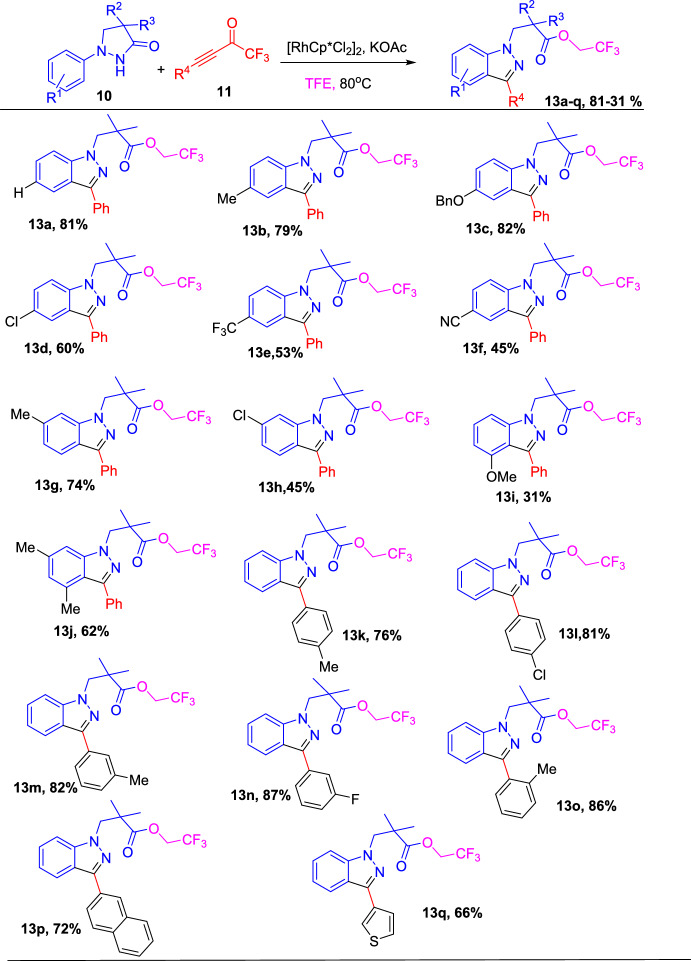
Scope of CF_3_-ynones

Further, the scope of the substrates was explored for the construction of (**13**), by investigating the generality of substrate **10** using **11a** as a model coupling partner. The outcomes are presented in Scheme 15. The investigation revealed that a variety of (**10**) having either alkyl and alkoxy (an electron-donating group) or halides, −CN and −CF_3_ (an electron-withdrawing group) at *para*-, *meta*-, or *ortho*-position of their phenyl ring reacted with **11a** in TFE gently to afford the products **13a−13j** in modest to superior yields. The experimental results suggested that the reactivity of (**10**) is reliant on the electronic characteristics of the attached substituent to some extent, and (**10**) with a strong electron-withdrawing group produced the respective indazole products in lower yields compared to those having an electron-donating group. In addition, the authors investigated the generality of the CF_3_-ynones (**11**) in the construction of (**13**). The results show that phenyl-substituted CF_3_-ynones with *p*-Me, *o*-Me (EDG) or *p*-Cl, *m*-F (EWG) units on the phenyl moiety reacted with **10** to produce the corresponding products **13k–o** in high yields. Next, the target indazole products **13p**, **13q**, and **13r** obtained good yields using naphthyl- and thienyl-substituted CF_3_-ynones.

**Scheme 16:**
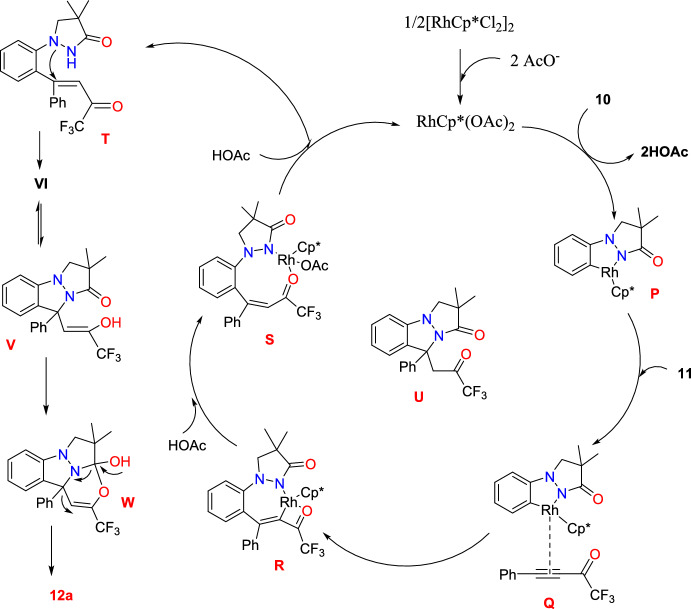
Plausible mechanism for CF_3_-tethered indazole **12a**

Scheme 16 describes the plausible mechanism for the preparation of **12a**, which was reported based on experimental evidence and control experiments. Initially, ligand exchange between NaOAc and [RhCp*Cl_2_]_2_ produces an active cationic Rh(III) species. Next, the five-membered rhodacycle intermediate coordination with the triple bond of **11** affords the intermediate **Q**. Further, the rhodium complex **R** generated **P** by consequent N−H/C−H bond metalation of **10** with Rh(III) by migratory insertion of the activated triple bond into the C–Rh bond. The intermediate **R**, on protonation, obtains the intermediate **S** that, on further protonation, gives the intermediate **T** and regenerates the active Rh(III) species. The electron-withdrawing solid effect of the −COCF_3_ unit causes rhodium to be more likely to be introduced into the α-position of the carbonyl group, which accounts for the regioselectivity perceived in the alkenylation. Next, the intermediate **T** on regioselective intramolecular nucleophilic addition affords the intermediate** U**, which endures a tautomerization to form the enol intermediate **V**. Concurrently, an intramolecular nucleophilic attack triggers a cascade transformation involving the formation of C–O bonds to produce the intermediate **W**, followed by cleavage of C–N/C–C bonds and opening of the pyrazolidinone ring, producing the target product **12a.** During the experimental investigations, the control experiments suggested that a Rh catalyst is not necessary for the transformation of **U** to **12a.**

### Through Rh-Catalyzed C–H Functionalization

**Scheme 17:**
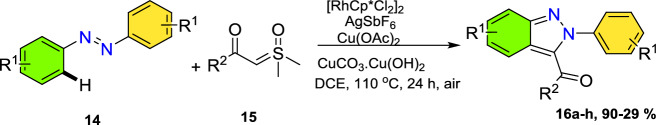
Rh(III)-catalyzed synthesis of 3-acyl-2*H*-indazoles

Kim’s group described prominent and chemoselective Rh(III)-catalyzed C–H functionalization and intramolecular annulation reactions between numerous azobenzenes and sulfoxonium ylides for the construction of 3-acyl (2*H*)-indazoles (Scheme 17) [83]. This method tolerates the production of an array of C3-acylated (2*H*)-indazoles having a higher degree of chemo-selectivity and substrate tolerance.

**Scheme 18:**
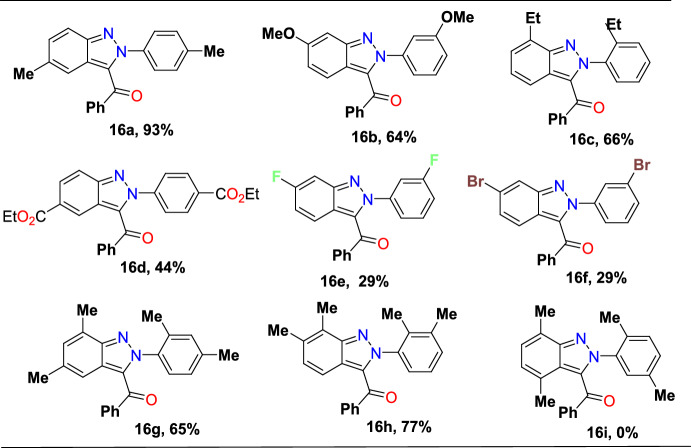
Scope of symmetrical azobenzenes

The functional group tolerance and limitation of this transformation were investigated under the optimal reaction conditions as shown in Scheme 18. The electron-rich *para-*substituted azobenzenes with *p*-Me, *m*-OMe, and *o*-Me substituents on both rings (**14a–c**) on reaction with 2-(dimethyl(oxo)-λ^6^-sulfaneylidene)-1-phenylethan-1-one (**15**) produced high yields of indazole adducts **16a**, **16b**, and **16c** in 93%, 64%, and 66% yields, respectively. However, electron-deficient azobenzene with *p*-COOEt, *m*-F, and *o*-Et on similar reactions gave the target indazoles **16d**, **16e**, and **16f** in 44%, 29% and 30% yields, respectively. Moreover, disubstituted azobenzenes with 2,4-diMe and 2,3-diMe units on both the rings were also compatible to afford **16g** (65%) and **16h** (77%), respectively. However, sterically congested 2,5-diMe disubstituted azobenzene did not undergo the coupling reaction to afford the consecutive 3-acyl (2*H*)-indazole **16i**.

**Scheme 19:**
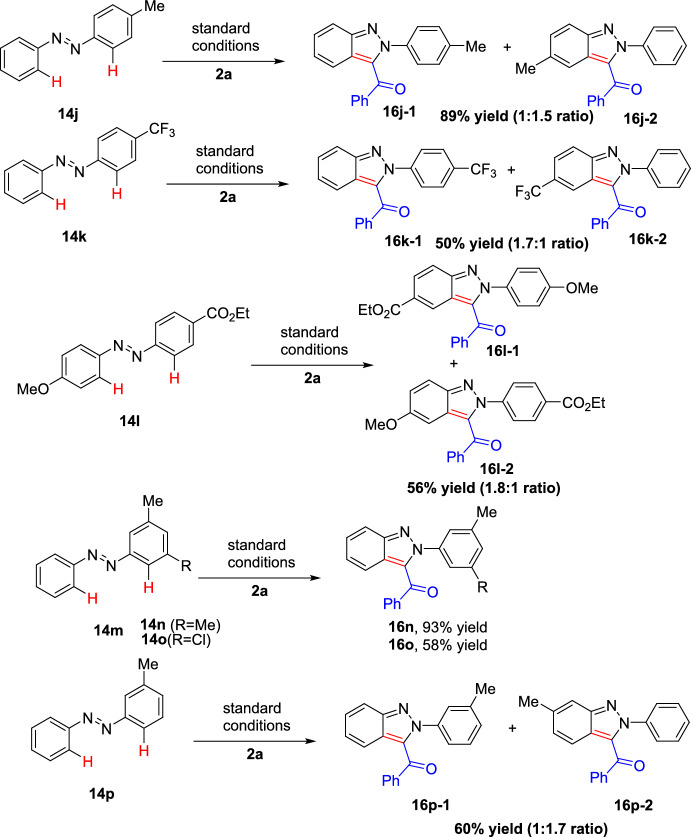
Scope of unsymmetrical azobenzenes

Additionally, with unsymmetrical azobenzenes **14j−p**, the reactions mainly took place at the C−H bonds on the electron-rich aromatic ring to provide the corresponding indazoles (**16j-2**, **16k-1**, and **16l-1**) as major products (Scheme 19). The authors also investigated the steric effects on azobenzenes, and the products **16n** and **16o** were obtained in 93% and 58% yields, respectively. The steric environment of azobenzene is also essential to control the regioselectivity of this transformation. Furthermore, the electronic and steric effects of this process were also observed by using unsymmetrical azobenzene **14p**, affording a mixture of 3-acyl (2*H*)-indazoles **16p-1** and **16p-2** in 60% combined yield with a 1:1.7 ratio.

**Scheme 20:**
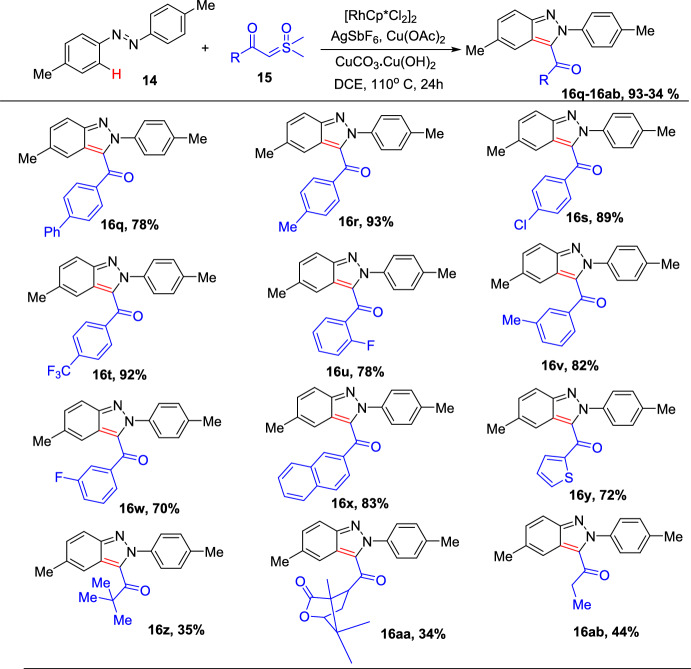
Scope of sulfoxonium ylides

To further explore the extent of this conversion, an extensive range of sulfoxonium ylides were tested for coupling with (*E*)-1,2-di-*p*-tolyldiazene, as shown in Scheme 20.

Sulfoxonium ylides bearing electron-rich or electron-deficient groups such as 4-ph, 4-Me,4-Cl, 4-CF_3_, 2-F, 3-Me, and 3-F on the phenyl ring on simple intramolecular annulation reactions afforded the resultant 3-acyl (2*H*)-indazoles **16q**–**w** in superior yields. This transformation also demonstrated a fine compatibility for sulfoxonium ylides containing naphthyl and heterocyclic moieties to afford the indazole products **16x** and **16y** in 83% and 72% yields, respectively. It was detected that this reaction was not only limited to aromatic sulfoxonium ylides, but the alkyl counterparts also contributed to the annulation procedure to supply the intended products **16z–16ab**–**16ab** in moderate yields, albeit with a bit of decreased reactivity.

**Scheme 21:**
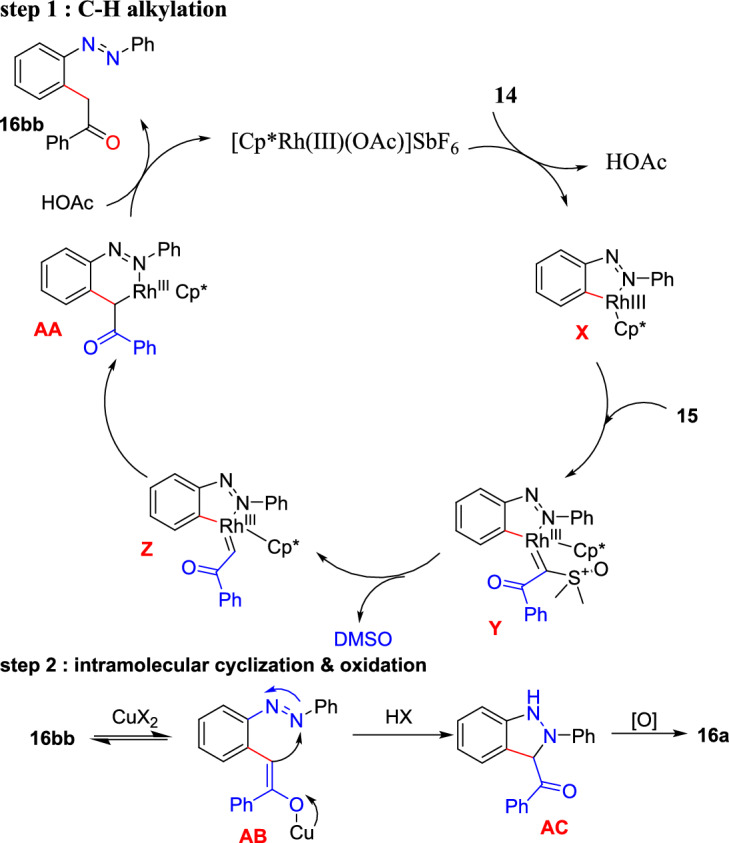
Plausible reaction mechanism for 3-acyl-2*H*-indazole**16a**

The predicted mechanism for the construction of 3-acyl-2*H*-indazole **16a** is depicted in Scheme 21. Coordination of a cationic Rh(III) catalyst with an azo group of **14** followed by C−H bond breakage gave a Rhoda-cycle species **X**, which on consequent coordination of sulfoxonium ylide **15** generates a Rh(III)-alkyl intermediate **Y**; this undergoes α-elimination of dimethyl sulfoxide (DMSO) to generate a reactive α-oxo-Rh-carbene species **Z**. Next, the alkylated product **16bb** was generated by protonation of six-membered rhodacycle **AA** produced from **Z** by migratory insertion. After that, the base-promoted intramolecular cyclization of **16bb** produces a 1,3-dihydroindazole intermediate **AB.** Finally, the intermediate **AB** on oxidation either with molecular oxygen or Cu(II) additives furnished 3-acyl (2*H*)-indazole **16a**.

### Pd-Promoted Synthesis

**Scheme 22:**
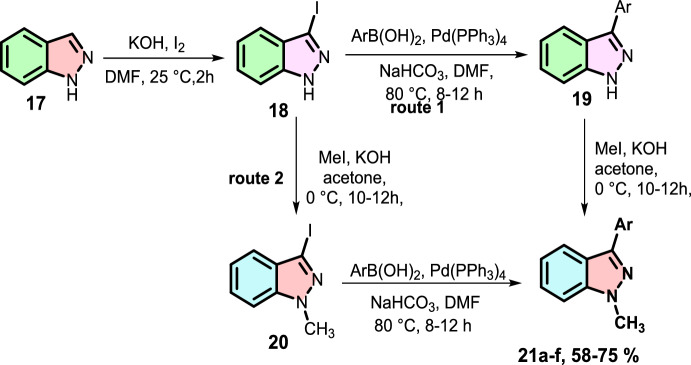
Pd-mediated construction of *N*-methyl-3-aryl indazoles

Bollikolla and group reported the successful Pd-mediated construction of *N*-methyl-3-aryl indazole scaffolds (Scheme 22) by two divergent approaches from starting material 1*H*-indazole via iodination, Suzuki–Miyaura coupling and methylation reactions [84].

**Scheme 23:**
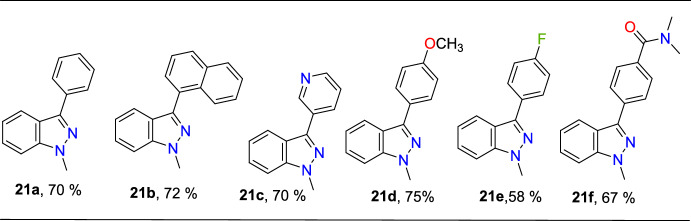
Synthesis of title compounds **21a–j**

The reaction shows extensive substrate tolerance with various aryl boronic acids for Suzuki–Miyaura coupling with 3-iodo indazole moieties to form the targeted indazoles **21a–f** in good yields, as shown in Scheme 23.

### Ag-Catalyzed Synthesis

**Scheme 24:**
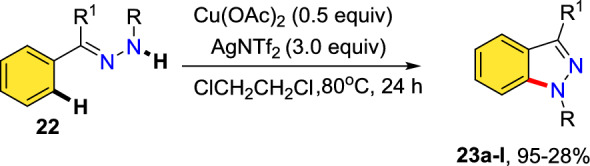
Ag(I)-catalyzed synthesis of 1,3-substituted indazoles

Lee’s group described the preparation of 1*H*-indazole motifs through inter-molecular oxidative C–H amination mediated by Ag(I) [85]. The technique involves efficient amination towards the production of a series of 3-substituted indazole motifs, which are otherwise difficult to prepare through other types of C–H amination reactions (Scheme 24). The preliminary mechanistic study suggested that the process occurs via an outer-sphere electron transfer catalyzed by the Ag(I) oxidant used. This procedure was identified as quite effective for the construction of a range of medicinally significant 1*H*-indazoles having amide, ester, olefine, ketone, −CF_3_, and aromatic units at the 3-position.

**Scheme 25:**
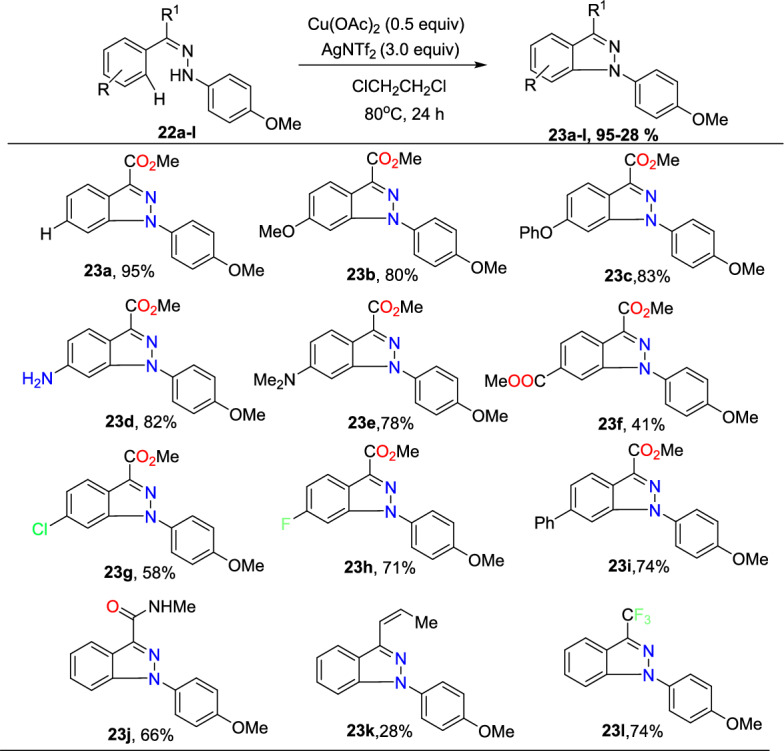
Substrate scope for 1,3-substituted indazole

At standard reaction conditions, the capacity of the reported amination was examined (Scheme 25). The unsubstituted arylhydrazone and aromatic hydrazones bearing electron-donating units at the *para*-position (**22a−e**) efficiently contributed to this amination to afford good to excellent yields of products **23a–e**. It is noteworthy that the substrate with an unmasked amine unit productively changed to the intended product (**23d**). It was also shown that the intramolecular amination of aromatic hydrazones bearing electron-withdrawing units was achieved and exhibited fine compatibility with substituents such as −COOMe, −Cl, and –F to afford the respective indazole scaffolds (**23f–h**) in good yields. Biaryl-derived arylhydrazone also gave product **23i** in good yield. This amination occurs to obtain the much more sterically accessible C−H bond if a *meta*-substituted arylhydrazone was examined. Notably, 1*H*-indazoles bearing amide, olefin, and even CF_3_ groups at the 3-position (**23j–l**) attained superior yields under the current C−H amination protocol.

**Scheme 26:**
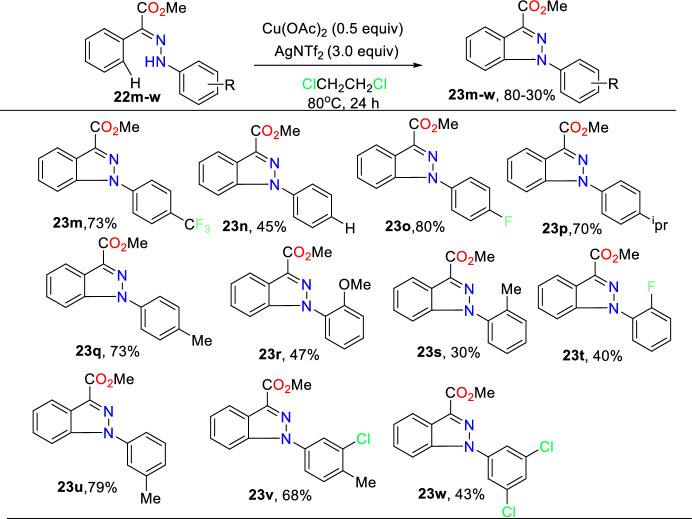
Substrate scope for 1-aryl substituents

Subsequently, the authors inspected the substrate tolerance with respect to the units on the 1-aryl ring of indazole (Scheme 26). An extensive range of functional units at the *para*-position fruitfully provides the indazole products (**23m–q**) with good yields apart from the electronic character of the substituents. Substrates having *ortho*-substituents (**22r–t**) or a *meta*-substituent (**22u**) were also examined, though with relatively lessened reaction proficiency, and produced the products in 30–79% yields. In addition, reactions with multi-substituted aromatic rings were found to be successful as well (**23v–w**), delivering the generality of reported amination to afford the target indazoles **23v** and **23w** in moderate yields.

**Scheme 27:**
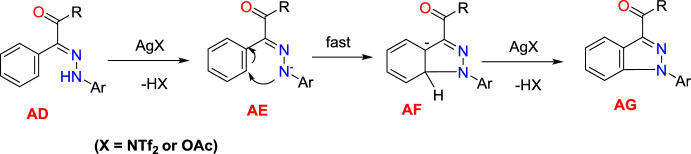
Plausible reaction mechanism 1,3-substituted indazole

The probable reaction mechanism was portrayed based on the standard literature and initial experiments (Scheme 27). First, the arylhydrazone (**AD**) undergoes oxidation via proton-coupled single-electron transfer (PCSET) to form a nitrogen-centered radical (NCR) intermediate (**AE**) induced by an equivalent amount of silver. Next, the intermediate **AE** on quick intramolecular C−N bond formation obtained the intermediate **AF**, which gave the required product **AG** by additional single-electron transfer (SET) oxidation followed by concomitant base-aided rearomatization. Based on the optimization studies, it was observed that Cu(II) is more likely engaged in deprotonation than oxidation of a substrate.

### Sn-Catalyzed Synthesis

**Scheme 28:**
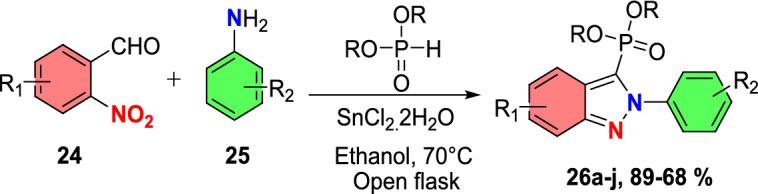
Synthesis of 2-aryl-2*H*-indazole-3-phosphonates

Sudhapriya et al. presented an efficient, eco-friendly, one-pot, tin(II) chloride dehydrate (SnCl_2_.2H_2_O)-promoted coupling followed by the reductive cyclization of primary amine (**25**), 2-nitrobenzaldehyde (**24**), and phenylacetylene or dialkyl phosphonates to attain 3-alkynyl-2-aryl-2*H*-indazole or 2-aryl-2*H*-indazole-3-phosphonates (Scheme 28) [86] by means of N–N bond formation. The reaction is economical and practical, and occurs at milder reaction conditions in open-flask conditions. This transition metal-free reaction showed broader substrate tolerance with superior yields; hence, it proceeded with a fine atom economy by eliminating H_2_O as a byproduct.

**Scheme 29:**
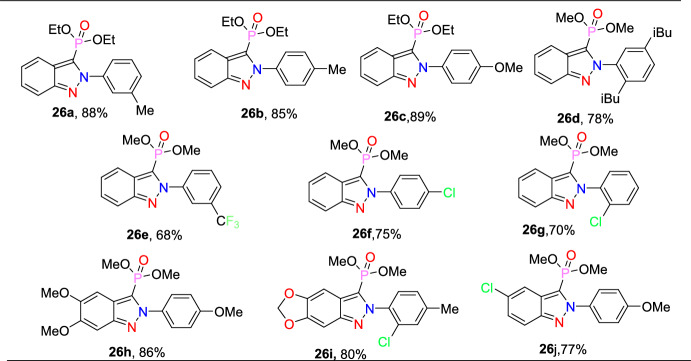
Substrate scope for 2-aryl-2*H*-indazole-3-phosphonates

The reaction scope was investigated by varying the 2-nitrobenzaldehydes to synthesize 2-aryl-2*H*-indazole-3-phosphonate analogues (Scheme 29). The reaction of 2-nitrobenzaldehyde (**24**) with various anilines (**25**) having electro-donating *m*–Me, *p*-Me, *p*-OMe, and 2,4-di^*i*^Bu units afford the respective indazoles **26a–d** in superior yields. Similarly, the 2-nitrobenzaldehyde with anilines bearing electron-withdrawing *m*-CF_3_, *p*-Cl, and *o*-Cl afforded the target indazoles **26e–g** in 68–75% yields. The reaction of 2-nitrobenzaldehyde (**24**) with 4-methoxy aniline at 70 °C for 2 h gave the indazole (**26h**) at 86% yield. Similarly, treatment of 2-nitrobenzaldehyde bearing O(−CH_2_)_2_ with 2-chloro-4-methylaniline afforded the corresponding product **26i** in 80% yield. A combination of 2-nitrobenzaldehyde bearing a chloro group with 4-methoxy aniline resulted in the subsequent product **26j** in good yield.

**Scheme 30:**
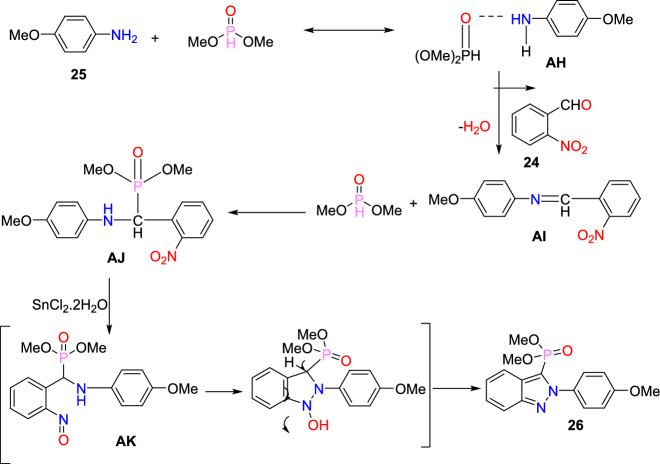
A possible mechanism for the construction of 2-aryl-2*H*-indazole-3-phosphonates

Based on literature reports and control experiments, the possible mechanistic path towards the production of indazole phosphonate **26** is described in Scheme 30. Most probably, the conversion was triggered by the less basic anisidine **24**, which manifests the proton donor property and produces a pre-reaction complex **AH** with the dimethyl phosphonate. Next, the 2-nitrobenzaldehyde **25** combines with intermediate **AH** from an imine (**AI,**), which on hydrophosphorylation, gave the α-aminophosphonate **AJ**. The nitroso compound **AK** was obtained by the reduction of α-aminophosphonate (**AJ**) in the presence of SnCl_2_.2H_2_O. Finally, the reduced nitroso group of **AJ** on the nucleophilic attack of amine and consecutive dehydration produced the target indazole **29**.

### Bimetallic (Pd/Cu)-Catalyzed Synthesis

**Scheme 31:**
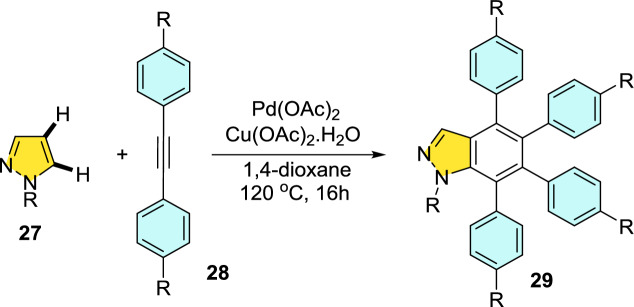
Construction of tetraaryl indazoles using Pd catalyst

An efficient Pd-mediated benzannulation of pyrazoles by internal alkynes to produce fluorescent indazoles was reported by Joo’s group (Scheme 31) [87]. They obtained the indazoles by the reaction of the C–H bonds of the pyrazole ring. This convergent approach directed the rise of tetraaryl indazoles as novel fluorophores. The authors synthesized varied indazoles with dissimilar units on the benzene ring using palladium(II) acetate as a catalyst and copper(II) acetate [Cu(OAc)_2_·H_2_O] as an oxidant.

In addition, this procedure proved to be expected and practical for other diazoles and imidazoles to give the consecutive benzannulated fused benzimidazoles. Complementary to several cyclization phenomena that generate the heterocyclic rings from functionalized arenes, this tactic has the advantage of straight translation of simple diazoles for the production of benzo-fused heteroarenes with numerous substituents on the benzene ring. These palladium-promoted and Cu-catalyzed benzannulation reactions offer a large variety of indazoles from simple pyrazoles in one step.

**Scheme 32:**
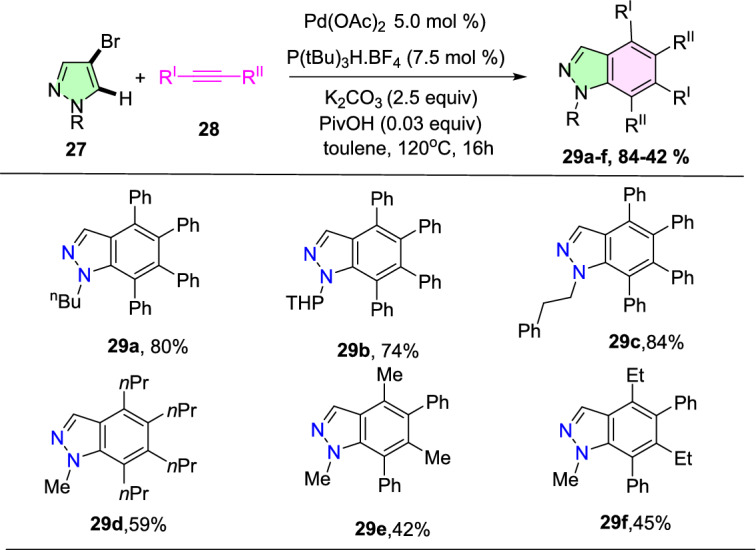
Substrate scope for the synthesis of tetraaryl indazoles

In an attempt to augment the regioselectivity, 4-bromo pyrazoles (**27**) were set up to facilitate the oxidative addition at the C-4 position. When 4-bromo pyrazoles (**27**) were subjected to the catalytic system comprising Pd(OAc)_2_ and P(^*t*^Bu)_3_H_·_BF_4_, the consequent indazoles were acquired in superior yields (Scheme 32). The 4-bromo-1-methyl-1*H*-pyrazole and the tetrahydropyranyl (THP)-protected pyrazoleon benzannulation reaction with 1,2-diphenylethyneprovided1-methyl-4,5,6,7-tetraphenyl-1*H*-indazole**29a**and THP produced 4,5,6,7-tetraphenyl-1*H*-indazole **29b** in good yield. Next, the 4-bromo-1-phenethyl-1*H*-pyrazole reacts with 1,2-diphenylethyne to afford the target indazole **29c** in 53% yield. Next, the 4-bromo-1-methyl-1*H*-pyrazole on benzannulation with oct-4-yne afforded the product **29d** in 59% yield. Moreover, the protocol is compatible with the unsymmetrical alkynes for benzannulation of the 4-bromo-1-methyl-1*H*-pyrazole to produce the target indazoles **29e** and **29f** in moderate yields. Furthermore, it was also reported that the method depending on the Pd(0)/Pd(II) catalytic cycle was somewhat more efficient than the oxidative benzannulation for the preparation of indazoles **29d–29f**.

### Cu-Catalyzed, Hydrazine-Free Synthesis

**Scheme 33:**
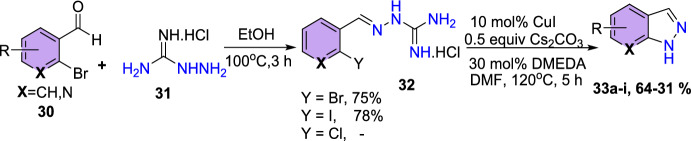
Construction of substituted 1*H*-indazoles using Cu catalyst

Rekowski’s group obtained the substituted 1*H*-indazoles via CuI-mediated, hydrazine-free conversion of 2-(2-bromoarylidene) guanylhydrazone hydrochlorides with high yields utilizing cesium carbonate as a base and *N*,*N*′-dimethylethylenediamine (DMEDA) as a ligand at 120 °C for 5 h (Scheme 33) [88]. Further, the quantum chemical calculations were used to study the reaction path. From these calculations and nuclear magnetic resonance (NMR) experiments, the substrate’s C=N double bond configuration was identified. It is crucial because it is one of the few that uses aminoguanidine hydrochloride, a less toxic nitrogen source, instead of the highly toxic hydrazines. An additional benefit is that this method produces the N-1 unsubstituted products in one step, i.e., it is not necessary to perform an extra deprotection step. The fact that both the catalyst and the starting materials are easily obtained and reasonably priced represents a third benefit.

Initially, during the investigation of optimization conditions for the cyclization of (E)-2-(2-halobenzylidene)hydrazine-1-carboximidamide hydrochloride, the outcomes reveal that among the halogens, iodine-containing substrates provided the target indazole effectively with slightly increased yields with the bromine-containing substrates. However, the chlorine-containing substrate did not produce the target product. However, in view of the difficulties in acquiring the substituted 2-iodo benzaldehydes, the authors chose to perform all further reactions with (*E*)-2-(2-bromo benzylidene)guanylhydrazone hydrochlorides.

**Scheme 34:**
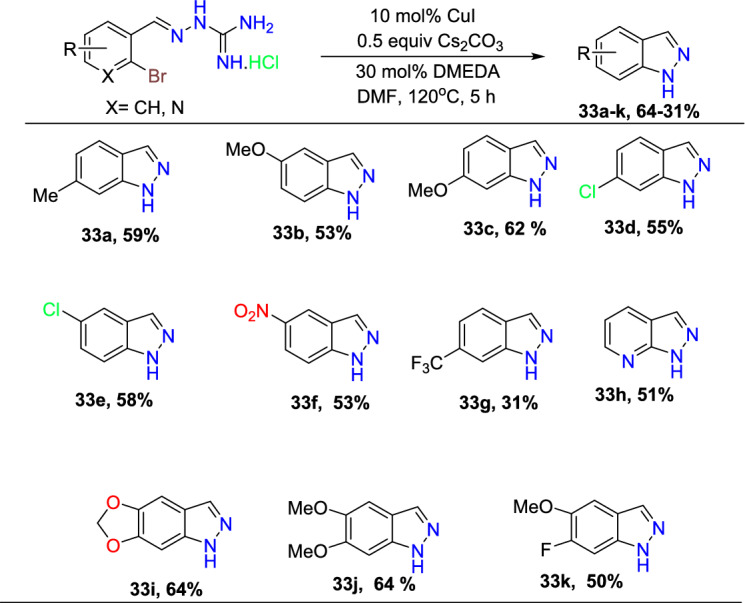
Synthesis of substituted 1*H*-indazoles **33a–k**

With optimization outcomes for the model cyclization, the authors next explored the substrate scope and constraints in the synthesis of the novel 1*H*-indazoles (Scheme 34). Initially, various 2-bromo benzaldehydes (**30**) were combined with the aminoguanidine hydrochloride (**31**) in EtOH at 100 °C for 3 h to obtain numerous substituted 2-(2-bromo benzylidene) guanylhydrazone hydrochlorides (**32**) in modest to superior yields. Having all the precursors in hand, the authors performed cyclizations under optimized conditions to obtain the 1*H*-indazoles **33a–i** as sole products in 31–64% yield. The 2-(2-bromo benzylidene) guanylhydrazone hydrochlorides (**32**) with the electron-donating 4-Me, 5-OMe, and 4-OMe units obtained the indazoles **33a–c** in 53–62% yield. Similarly, the 2-(2-bromo benzylidene) guanylhydrazone hydrochlorides having electron-withdrawing units like 4-Cl, 5-Cl, 5-NO_2_, and 4-CF_3_ substituents afford the target indazoles **33d–g** in 49–55% yield. Interestingly, the substrate with the electron-withdrawing 5-NO_2_ and 4-CF_3_ groups produced the consequent products **33f** and **33g**, respectively, in good yields. Interestingly, the experimental results revealed that the starting materials with halogen at C-4 provided lower yields of the target indazoles compared to 5-halogen-substituted substrates. Next, the pyridine-based substrate generated the 1*H*-pyrazolo[3,4-b]pyridine (**33j**) in 51% yield. Moreover, diverse 4,5-disubstituted substrates undergo a clean cyclization to the subsequent 5,6-disubstituted target products **33i–k** in 64–50% yield.

**Scheme 35:**
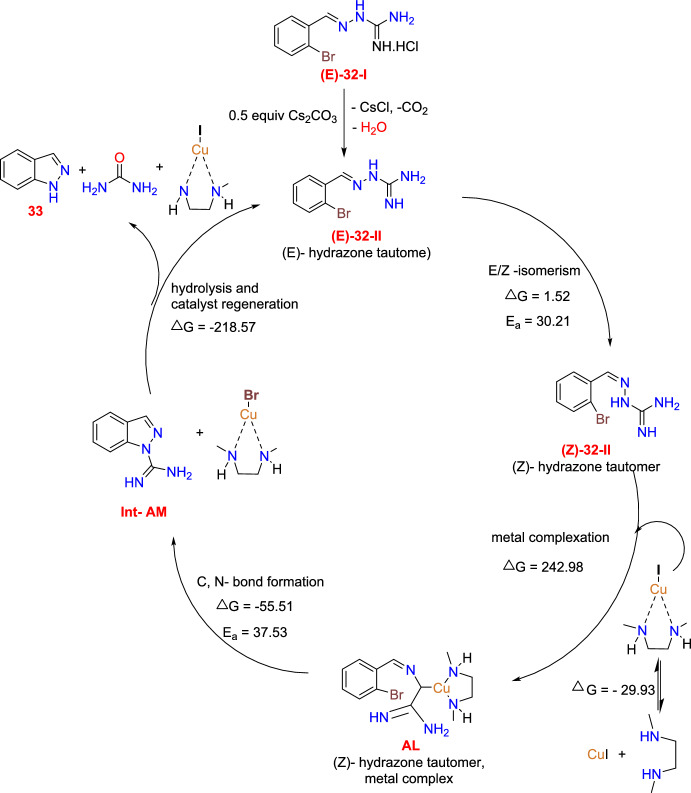
A possible mechanism for substituted 1*H*-indazoles **33a**

Scheme 35 illustrates the proposed mechanism based on the density functional theory (DFT) study results. Initially, neutralization of the guanylhydrazone hydrochloride of ((***E)-32-I)*** with Cs_2_CO_3_ forms the hydrazone tautomer (***E***)-**32-II.** Next, this hydrazone tautomer undertakes E/Z-isomerization to form the resultant hydrazone tautomer **(Z)-32-II**, which undergoes complexation with the CuI-DMEDA complex to produce a metal complex **AL**. This complex **AL** undergoes an intramolecular C–N bond construction that leads to the formation of intermediate **Int-AM** and a CuBr-DMEDA complex. Finally, the hydrolysis of **Int-AM** delivers 1*H*-indazole (**2a**) and urea, along with the regeneration of the CuI-DMEDA complex. Interestingly, the DFT calculations performed by the authors do not support a classic oxidative addition/reductive elimination sequence; as an alternative, they recommended that the metal complex of **(Z)-32-II** (hydrazone tautomer) operates as the direct precursor of the intramolecular cyclization.

### Cu-Promoted Two-step Syntheses of Indazole Carboxylic Acids

**Scheme 36:**
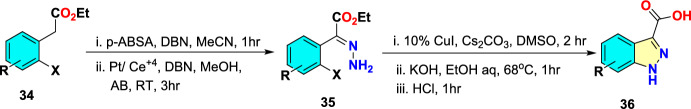
Synthetic route for indazole carboxylic acids

Laxman’s group described a two-step preparation of indazole carboxylic acids through a C–N cyclization (Scheme 36) [89]. In the first step, the one-pot union of carboxylic ester (**34**) was treated with *p*-ABSA and CH_3_CN, followed by drop-wise blending with 1,5-diazabicyclo[4,3,0]non-5-ene (DBN). This blend was mixed for 1 h at room temperature. Then, Pt/ Ce^4+^ (nano-CeO_2_) catalysts and MeOH were added and sonicated the total reaction mixture for 15 min to obtain the E-hydrazone esters (**35**). In the second step, the obtained E-hydrazone esters **35a–h** on CuI-catalyzed cyclization using Cs_2_CO_3_ base under DMSO for 2 h gave the respective indazole esters, which on reflux condensation with KOH and EtOH at 68 °C and hydrolysis result in the corresponding indazole carboxylic acids **36a–h** in superior yields.

**Scheme 37:**
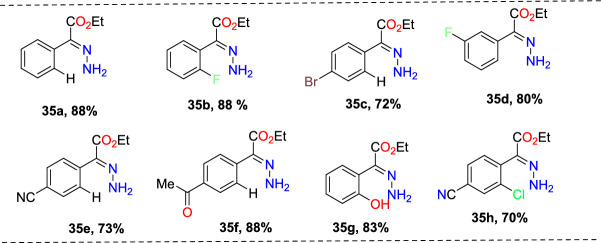
Synthesis of E-hydrazone esters **35a–h**

**Scheme 38:**
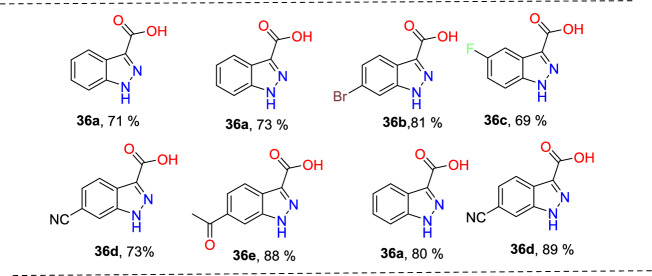
Substrate scope for indazole carboxylic acids

Initially, various carboxylic esters (**34**) afford the respective E-hydrazones (**35**) in good to excellent yields (Scheme 37). The unsubstituted ethyl 2-phenylacetate and mono-substituted ethyl 2-phenylacetates with 2-F, 4-Br, 5-F, 4-CN, 2-OH, and 4-COOMe units under standard reaction conditions (Step 1, Scheme 36) afford the consecutive E-hydrazone esters **35a-g** in good to excellent yields. Similarly, the ethyl 2-(2-chloro-4-cyanophenyl)acetate produced the respective E-hydrazone esters **35h** in 70% yield. Next, the unsubstituted E-hydrazone (**35a**) and the E-hydrazone with 2-F and 2-OH substitutions (**35b** and **35g**) on C–N cyclization produced the same product **36a** in 71%, 73%, and 80% yields respectively (Scheme 37). Similarly, the *E*-hydrazones 4-Br, 5-F, and 4-COCH_3_ units afforded the products **36c**, **36d**, and **36f** in 69–88% yields on C–N cyclization (Scheme 38).

### Using Cu(I) Complex of Dihydro Bis(2-mercapto benzimidazolyl) Borate Catalyst

**Scheme 39:**
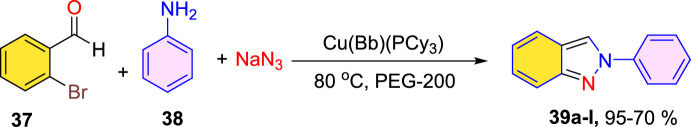
Synthetic path for 2-aryl-2*H*-indazoles

Dariush’s group illustrated the construction of 2-aryl-2*H*-indazoles [90] using a Cu(I) complex of dihydro bis(2-mercapto benzimidazolyl) borate and tricyclo hexylphosphine ligands [Cu(Bb)(PCy_3_)] as an efficient homogeneous catalyst in PEG-200 as a green media (Scheme 39). This method is valuable due to its milder reaction conditions, avoiding explosive hydrazoic acid, ease of work-up, broad applicability, and short reaction times. The added catalyst needed a simple handling procedure in addition to its nontoxicity and potent σ-donating capacity. In large-scale experiments, this Cu-based catalytic system is also noteworthy for its ability to produce the intended product with excellent yields. This approach can accommodate various functional groups and eliminates the need for costly and hazardous catalysts and reagents.

**Fig. 4:**
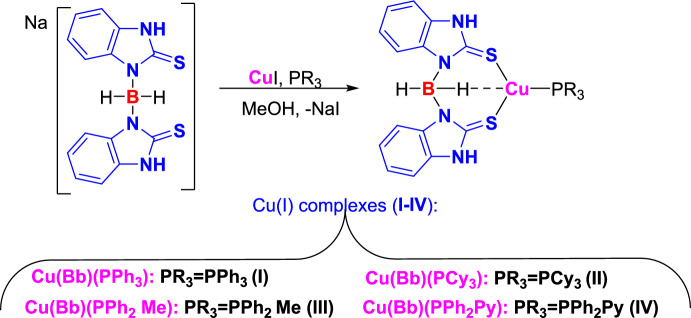
Synthetic route for Cu(I) complexes

The used Cu(I) complexes, Cu(Bb)(PPh3) (I), Cu(Bb)(PCy3) (II), Cu-(Bb)(PPh2Me) (III), and Cu(Bb)(PPh2Py) (IV) were obtained as per the reported protocol (Fig. 4) [89].

**Scheme 40:**
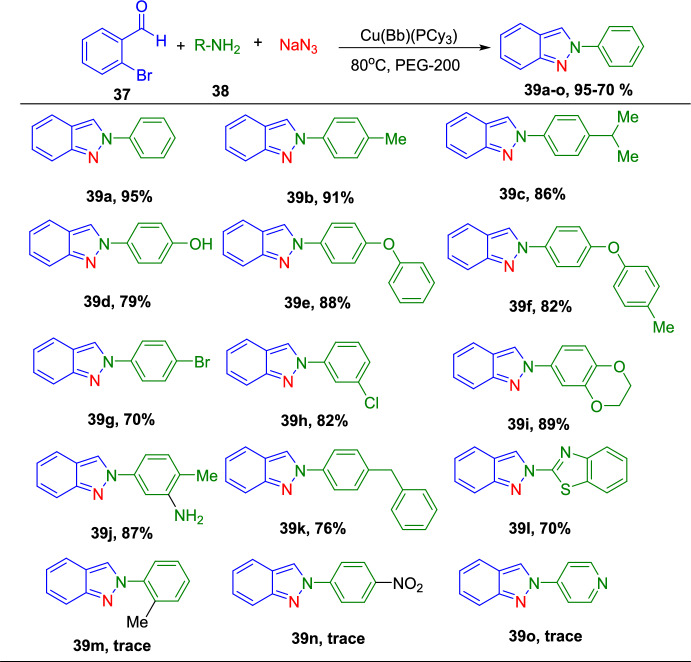
Synthesis of 2-aryl-2*H*-indazoles **39a–o**

Under ideal conditions, the scope of the substrates for the reported protocol was explored (Scheme 40). Primarily, under standard conditions, 2-bromo benzaldehyde reacted with aniline to obtain the 2-phenyl-2*H*-indazole (**39a**) in an excellent 95% yield. Further, the anilines containing electron-donating units at the *para*-position, such as −Me, −^*i*^Pr, −OH, −OC_6_H_5_, −OC_6_H_4_Me, and –Br units, reacted with 2-bromo benzaldehyde and sodium azide to produce the respective indazole scaffolds **39b–f** in good yields. The anilines with halo substitutions like 3-Cl also produced consecutive indazoles of **39h** in good yield. Remarkably, bicyclic anilines like 2,3-dihydrobenzo[*b*][1,4]dioxin-6-amine could also be effectively changed to the consequent target indazole product **39i** in excellent yield. Furthermore, the resultant indazole product **39j** was obtained in a high yield by the smooth reaction of aniline bearing two electron-releasing (−Me and −NH_2_) substitutions with sodium azide and 2-bromo benzaldehyde. 2-(4-Benzylphenyl)-2*H*-indazole (**39k**) was obtained in 76% yield from the aliphatic amine benzylamine. Gratifyingly, the bioactive motif 2-aminobenzo thiazole gave the respective indazole **39l** with a 70% yield. Unfortunately, the sterically hindered *ortho*-substituted aniline *o*-toluidine gave the analogous 2*H*-indazole product **39m** only in trace amounts, indicating the negative influence of steric bulk on the reaction. Lastly, anilines having an electron-withdrawing *p*-NO_2_ unit and heterocyclic anilines, like 4-aminopyridine, did not form the respective 2*H*-indazole products **39n** and **39m**.

**Scheme 41:**
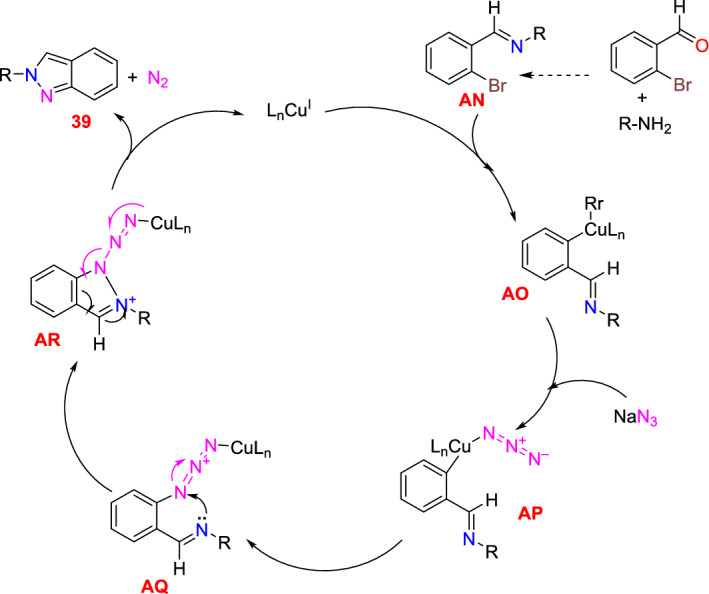
Possible mechanism for 2-aryl-2*H*-indazole formation

Scheme 41 illustrates the plausible mechanism for the Cu(Bb)(PCy_3_)-catalyzed formation of 2*H*-indazole derivatives predicted on the basis of sequential condensation/C–N and N–N bond formation/aromatization transformations. First, 2-bromo benzaldehyde condenses with primary amines with the aid of a catalyst to form an imine intermediate **AN**. Following the imine formation, Br is activated using the azide ion and the Cu(Bb)(PCy_3_) complex (intermediate **AO**) to obtain intermediate **AP**. Later, this intermediate **AP** is electronically reorganized (via intermediates **AQ** and **AR**), and the corresponding 2*H*-indazole is produced by nitrogen extrusion and Cu(I) catalyst regeneration. This intermediate then endured electronic reorganization (via intermediates **AQ** and **AR**) followed by the nitrogen extrusion and Cu(I) catalyst regeneration to achieve the corresponding target 2*H*-indazole (**39**).

### Ionic Liquid-Supported Copper-Catalyzed Synthesis Under Microwave Irradiation

**Scheme 42:**
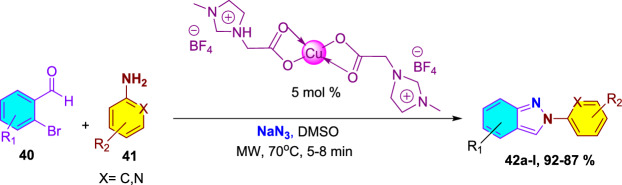
Synthetic route for 2-aryl-2*H*-indazoles **42a–l**

Upala et al. described an efficient and greener ionic liquid-supported Cu-catalyzed construction of 2-aryl-2*H*-indazoles by a one-pot multi-component reaction of aromatic amines with 2-bromo benzaldehydes in the presence of sodium azide, under microwave irradiation [91]. This process is more eco-friendly and highly energy-efficient than the conventionally reported methods by employing microwave and ionic liquid-supported catalysts. Overall, in this protocol, the authors used an ionic liquid-supported Cu catalyst to form consecutive C–N and N–N bonds to afford the targeted 2*H*-indazole motifs in very high yields (Scheme 42). It is noteworthy that under neat conditions at an elevated temperature of 100 °C, there is no formation of the desired 2*H*-indazole scaffold.

**Scheme 43:**
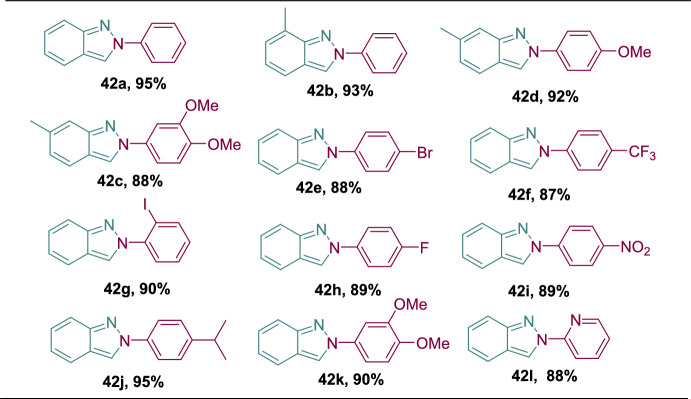
Synthesis of 2-aryl-2*H*-indazoles **42a–l**

In the presence of ionic liquid-supported Cu-catalyst, this protocol exhibits brilliant substrate scope by various 2-bromo benzaldehydes (**40**) and anilines (**41**) to afford the target indazole scaffolds **42a–l** in exceptional yields (Scheme 43). First, under the optimized reaction conditions, aniline reacted with 2-bromo benzaldehyde in the presence of NaN_3_ in DMSO to give the 2-phenyl-2*H*-indazole (**42a**) in excellent yields. Further, the 2-bromo benzaldehydes with an electron-donating 2-Me unit on reaction with aniline gave the indazole **42b** in 93% yield. Interestingly, 2-bromo-6-methylbenzaldehyde with 4-OMe and 3,4-diOMe produced the products **42c** and **42d**, respectively, in 92% and 88% yields.

Next, the reaction of 2-bromo benzaldehyde with aniline carrying various electron-withdrawing units such as 4-Br, 4-CF_3_, 2-I, 4-F, and 4-NO_2_ produced the target indazoles **42e–i** in 88–90% yields. Similarly, the 2-bromo benzaldehyde reaction with anilines having electron-donating 4-^*i*^Pr, 3,4-diOMe substitutions gave the indazoles **42j** and **42k** in excellent yields. Finally, the heteroaniline, such as pyridin-2-amine combined with 2-bromo benzaldehyde, under standard reaction conditions afforded the 2-(pyridin-2-yl)-2*H*-indazole in 88% yield. Aryl amines with an electron-withdrawing group resulted in a lower yield than electron-releasing groups. However, arylamines with greater steric hindrance gave the target 2*H*-indazoles comparatively in lower yield.

**Scheme 44:**
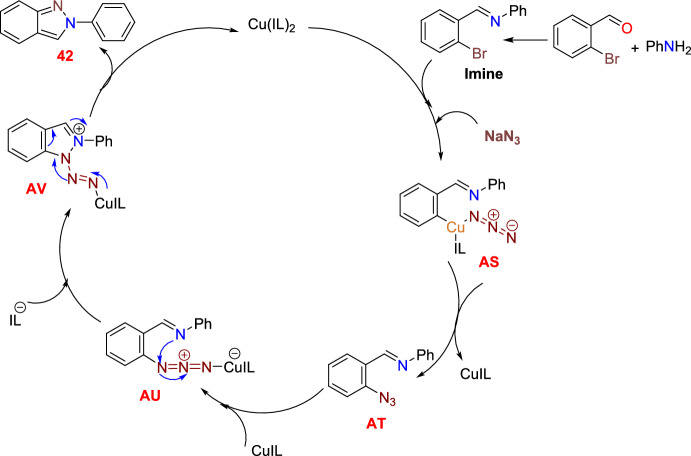
Plausible mechanism for construction of 2-phenyl-2*H*-indazole **42a**

Scheme 44 describes the proposed reaction mechanism for the catalytic heterocyclization reaction. Primarily, arylamine and bromobenzaldehyde are expected to react to obtain the imine. Next, the imine is in harmony with the Cu catalyst, and the substitution of bromine by azide leads to the intermediate **AS**, which gives the intermediate **AT**, which is further transformed into the copper(I)-azide complex **AU**. Next, the azide’s terminal nitrogen atom is coordinated with the Cu catalyst, which causes the activation of the azide. On the other hand, a N–N bond was formed by the attack on active azide by the nitrogen atom of benzylidene aniline to form the intermediate **AV**. Finally, the targeted 2*H*-indazole motif was obtained by the emission of nitrogen gas followed by the regeneration of the ionic liquid-supported Cu catalyst, which facilitated a continuous, self-improving, reusable synthetic strategy.

### Cu-Catalyzed Intramolecular *N*-arylation of *o*-Chlorinated Arylhydrazones

**Scheme 45:**
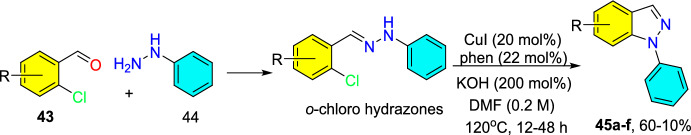
One-pot synthesis of *N*-phenyl-1*H*-indazoles

Yara et al. reported the construction of *N*-phenyl- and *N*-thiazolyl-1*H*-indazoles via a Cu-promoted intramolecular *N*-arylation of inexpensive and readily available *o*-chlorinated arylhydrazones (Scheme 45) [92]. This is the first report on the synthesis of pharmacologically significant N-thiazolyl motifs, and it also promoted the preparation of *N*-phenyl-1*H*-indazoles (**45a–f**) in a superior yield. Using this method, a group of *N*-phenyl motifs and *N*-thiazolyl scaffolds (**47a–d**) were obtained in 10–70% and 12–35% yield, respectively, by stirring the *o*-chloroaryl hydrazones, KOH, CuI, and 1,10-phenantroline for 12–48 h in dimethylformamide (DMF) at 120 °C.

**Scheme 46:**
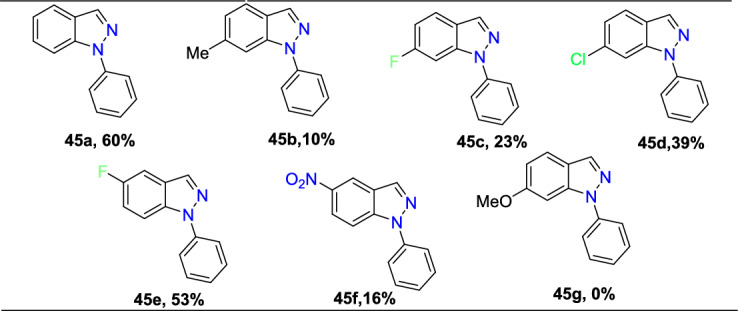
Substrate scope for *N*-phenyl-1*H*-indazoles

Various* o*-chlorobenzaldehydes reacted with phenylhydrazene to afford the respective *o*-chlorinated arylhydrazones, which on Cu-promoted intramolecular *N*-arylation, produced the *N*-phenyl-1*H*-indazole derivatives **45a–f** in 10-60% yields (Scheme 46). The structure of the starting materials generally had a significant influence on the reactions. The *o*-chlorinated benzaldehyde combines with phenylhydrazine to form (E)-1-(2-chlorobenzylidene)-2-phenylhydrazine (**45a**) in 60% yield under standard reaction conditions. Similarly, the *o*-chlorinated arylhydrazones obtained from starting material *o*-chlorobenzaldehyde with additional substituents such as 4-Me (electron-donating), 4-F, and -4-Cl (electron withdrawing) produced the consecutive ***N***-phenyl-1*H*-indazole derivatives **45b–d** in low yields. The 2-cholro-5-fluoro benzaldehyde in reaction with phenylhydrazine gave the respective arylhydrazones that produced the consecutive indazole **45e** in 53% yield. The substrates with 4-F and 5-F substitutions needed longer reaction times (39–48 h) to increase the yields. Interestingly, the 5-NO_2_-substituted arylhydrazone obtained the target indazole **45f** with 16% yield in a shorter reaction time of 12 h. The 4-methoxy containing arylhydrazone was not transformed at all to the required 1*H*-indazole **45g**, indicating that the arylhydrazones with electron-donating units at the 4-position showed inefficiency towards this method.

**Scheme 47:**
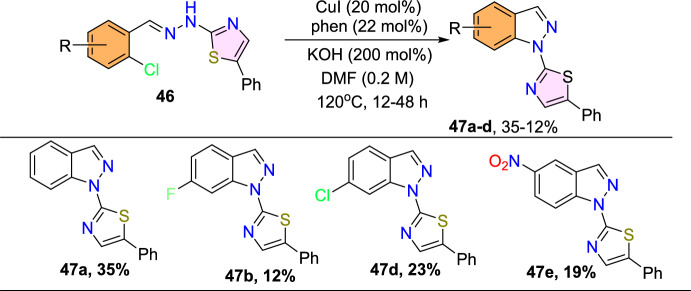
Substrate scope for*N*-thiazolyl-1*H*-indazoles

Next, the authors applied identical conditions to translate *N*-thiazolyl-substituted arylhydrazones (**46**) to the subsequent *N*-thiazolyl-1*H*-indazoles **47a–d** (Scheme 47). The results revealed that the yields were lower comparable to *N*-phenyl-1*H*-indazole series products. In this instance, the starting material structure also affected the product yield. Unsubstituted *N*-thiazolyl arylhydrazone yielded the products **47a** in 35% yield. *N*-Thiazolyl arylhydrazones with electron-withdrawing −F, −Cl, and −NO_2_ substituents gave the respective products **47b–d** in 12–23% yield only. Moreover, it was also reported that the conditions were not favorable for transforming arylhydrazones with donating units at the 3-position, like 3-Me and 3-OMe.

### Synthesis Using an Intramolecular Ullmann-Type Reaction

**Scheme 48:**
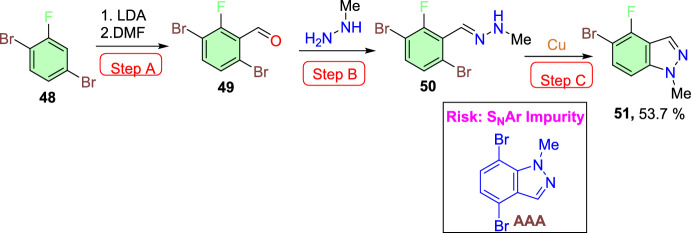
Route for the synthesis of fluorinated indazoles

Zhang’s group developed an improved and concise synthetic path towards fluorinated indazole motifs [93], which is an active pharmaceutical ingredient intermediate. The reported route proceeds through an electronically promoted metalation/formylation series followed by the condensation with methylhydrazine to obtain a hydrazone. Finally, it ends with a Cu-catalyzed intramolecular Ullmann cyclization to generate the aspired indazole (Scheme 48). The practical difficulties of the Ullmann reaction, like poor reactivity and thermal hazard concerns, were overcome by this method using safe and optimal conditions, statistical modelling, and an unusual isolation method that leads to the isolated material in high purity with brilliant yields at a laboratory scale. This method is helpful for the preparation of critical intermediates for active pharmaceutical ingredients. The authors mitigated the safety risks deriving from the utilization of unsafe reagents, solvents, or combinations thereof.

During the exploration of the paths towards the construction of 5-bromo-4-fluoro-1-methyl-1*H*-indazole (**51**), it was expected that the electrons of the aryl fluorine at the *ortho*-position fluorine in the starting material 1,4-dibromo-2-fluorobenzene (**48**) are required that could direct the lithiation electronically. Next, the dimethyl formamide reacted with the produced lithiate and delivered the 3,6-dibromo-2-fluorobenzaldehyde (**49**) (Scheme 48, step A). Subsequently, the aldehyde (**49**) condensed with methyl hydrazine afforded the hydrazone intermediate (**50**) (Scheme 48, step B). Finally, the hydrazone intermediate (**50**) on intramolecular Ullmann ring closure (Scheme 48, step C) provided the targeted 5-bromo-4-fluoro-1-methyl-1*H*-indazole (**51**) directly in 53.7% yield on chromatographic isolation. This strategy was peril-free, as it could be anticipated that the hydrazone (**50**) could undergo an unwanted S_N_Ar ring closure of secondary nitrogen of the hydrazone onto the fluorine attached to aromatic carbon to obtain **LI**, which was supposed to result in downstream impurities if not controlled.

**Scheme 49:**
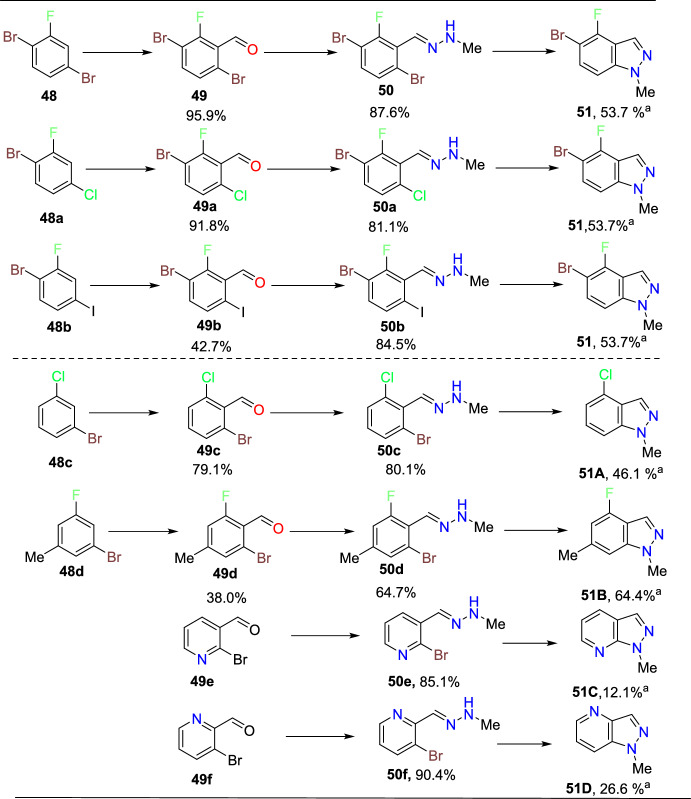
Substrate scope for fluorinated indazoles. ^a^ Chromatographicisolated yields; compounds **49e** and **49f** are purchased commercially available materials

The scope of the protocol was then tested by exploring the generality of the enhanced Ullmann conditions (Scheme 49). The 1-bromo-4-chloro-2-fluorobenzene (**48a**) and 1-bromo-2-fluoro-4-iodobenzene (**48b**) reacted with dimethyl formamide and delivered the aldehydes **49a and 49b**, respectively. Next, these aldehydes condensed with methyl hydrazine afforded the respective hydrazone intermediates **50a** and **50b**. Finally, these hydrazone intermediates on intramolecular Ullmann ring closure provided the same 5-bromo-4-fluoro-1-methyl-1*H*-indazole (**51**) product directly in good yield. For the chlorinated analogue (**48a**), the halogen variation in the formylation step goes smoothly; in contrast, the iodine analogue (**49a**), most likely because of the lability of the C–I bond apparent aryne reactivity, leads to dimeric impurities that significantly hampered the reaction due to apparent aryne reactivity.

In every case, as anticipated, the methyl hydrazine condensed with the generated aldehyde to form the hydrazone in superior yields. In Ullmann’s steps, the variation of the halogen (ring closure of **50a** and **50b**) revealed the tactic of employing a bromine in the C−X bond. A prolonged intramolecular ring closure occurs when chlorine is substituted (ring closure to **51** from **50a**). Even though some products are produced, an early equal amount of respective S_N_Ar impurity is produced while using CuCl as a catalyst. Similar to the reaction seen with bromine, comparable conversion occurs even when the halogen was switched to iodine (ring closure to **51** from **50b**), with the exception that a significant des-iodo hydrazone impurity results from the comparative lability of the C–I bond. In other words, the uniqueness of the reacting halogen reveals an envelope of reactivity that is fascinating since, while a C−Cl bond is ostensibly too strong, the C−Br bond works well.

In contrast, the C−I bond is fragile and is likely to hydrodehalogenate beneath the reaction conditions. For hydrazones with electron-withdrawing substituents at the *ortho*-position, the reaction proceeds similarly to form the respective indazole scaffolds (**51A**, **50B**). Pyrazolopyridines (**51C**, **51D**) can also be prepared using this method. Interestingly, both of them are volatile. In general, the intramolecular Ullmann cyclization of electron-deficient arenes proceeds similarly to substrates containing bromine as the reacting halogen.

**Scheme 50:**
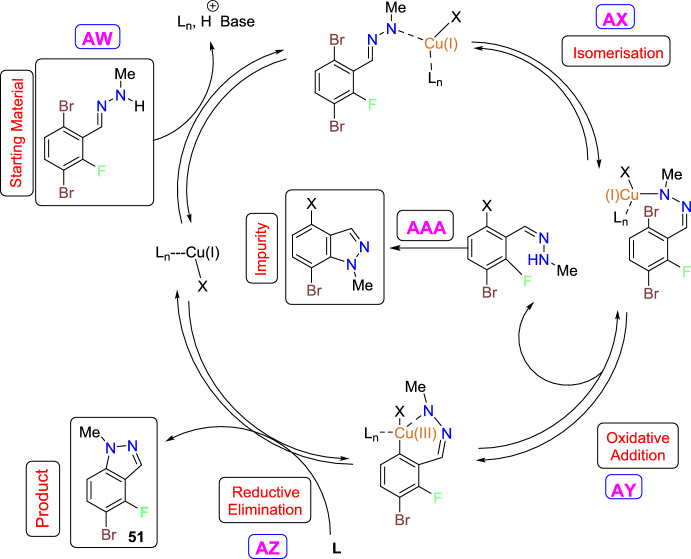
Presumptive Ullmann mechanism for fluorinated indazole (**51**)

The presumptive mechanism was presented in Scheme 50. The starting material, in coordination with the Cu catalyst and deprotonation by DBU, produces **AW**. The copper catalyst’s Lewis acidity may facilitate a tautomerization event, or it may just remove adequate electron density from the hydrazone system to reduce the thermal barrier to isomerization, allowing bond rotation in either scenario (**AX**). Another possibility is that DBU is involved in this as well. A transient nucleophilic attack on the benzylic position would rearrange electron density to permit bond rotation about the previous double bond before expelling DBU and reforming the double bond. This isomerization event, however, needs to result in a geometry that permits the oxidative addition (**AY**) of Cu catalyst to a formal Cu(III) complex, which then, on reductive elimination (**AZ**), yields the desired product (**51**) along with observed S_N_Ar impurity (**AAA**).

### Cu-Catalyzed Synthesis

**Scheme 51:**
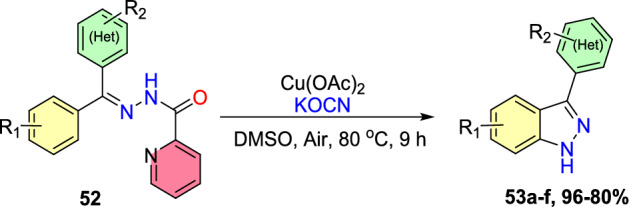
Cu-mediated construction of 3-aryl-1*H*-indazoles

A traceless picolinamide directing group-aided copper-promoted oxidative intramolecular C–H amination of hydrazones for the preparation of 1*H*-indazoles was described by the Ding group (Scheme 51) [94].

This method was very effective by facilitating the direct formation of *N*-heterocyclic compounds employing picolinamide as a traceless guiding group and the low-cost copper salts as oxidants with extensive functional-group compatibility in producing modest to superior yields of target indazoles **53a–f**.

**Scheme 52:**
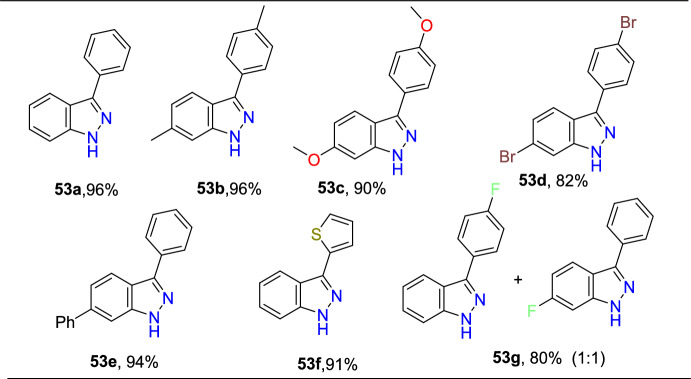
Substrate scope for 3-aryl-1*H*-indazoles

Under the optimized reaction conditions, different substrates were reacted to test the generality and scope (Scheme 52). Initially, the *N*′-(diphenylmethylene)picolinohydrazide on copper-promoted oxidative intramolecular C–H amination afforded the consecutive indazole product 3-phenyl-1*H*-indazole **53a** in 94% yield. Importantly, all substrates examined obtained the target products in modest to brilliant yields. Generally, the oxidative amination reaction was uninfluenced by the electronic characteristics of the substituents on the arene ring. Substrates with monosubstitution bearing two identical groups such as −Me and −OMe (electron-donating) and −Br (electron-withdrawing) on the *para*-position of the aromatic rings gave only corresponding products **53b–d** with 82–96% yields. Next, the (*E*)-*N*′-([1,1′-biphenyl]-4-yl(phenyl)methylene)picolinohydrazide on similar reaction afforded the target 3,6-diphenyl-1*H*-indazole **53e** in 94% yield. Amazingly, the substrate bearing thienyl also shows good reactivity in this reaction to furnish the product **53f** in superior yield. The reaction of hydrazone (**52**) with a mono-fluoro (electron-withdrawing) substituent produced a regioselective mixture of the product (**53g**) in a 1:1 ratio with a good yield of 80%.

**Scheme 53:**
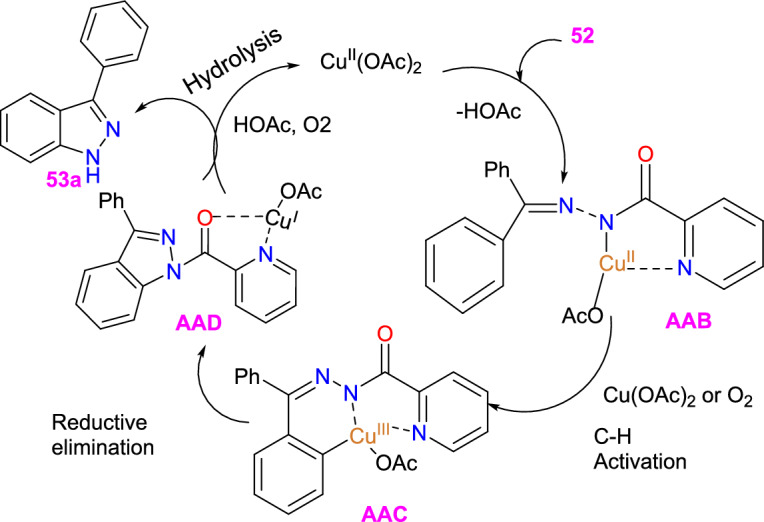
Possible mechanism for 3-phenyl indazole **53a**

Based on introductory mechanistic studies and the relevant previous reports, the possible reaction mechanism (Scheme 53) is depicted with compound **52** as the model substrate. Initially, the reaction ensued by the coordination of Cu(OAc)_2_ with **1a** to form species **AAB** and release HOAc. Subsequently, intermediate **AAB** endures C−H activation and oxidation to generate a six-membered intermediate **AAC**, which undergoes reductive elimination to obtain the species **AAD** and Cu(I) species. Finally, the hydrolysis of intermediate **AAD** directed to the required product **53** might be due to the activation of the amide group by the coordination of Cu(I) species with intermediate **AAD** via N,O coordination. The Cu(I) species could be oxidized with HOAc and O_2_ for the reproduction of Cu(OAc)_2_ for the next catalytic cycle.

### Cu(OAc)_2_-Promoted C–N Bond Formation

**Scheme 54:**
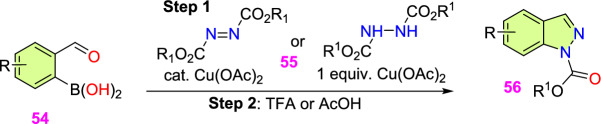
Cu(OAc)_2_ promoted construction of 1*N*-alkoxycarbonyl indazoles

Jirgensons and co-workers reported an effective Cu(OAc)_2_-promoted construction of 1*N*-alkoxy carbonyl indazoles (**56**) from the reaction of 2-formyl boronic acids (**54**) with diazadicaboxylates (**55**) followed by acid/base-facilitated ring closure by the development of a C–N bond (Scheme 54) [95]. A stoichiometric amount of Cu(II) acetate was required for the C–N bond development step. This one-pot, two-step protocol can be carried out under reasonably moderate reaction conditions and is based on easily accessible building blocks.

**Scheme 55:**
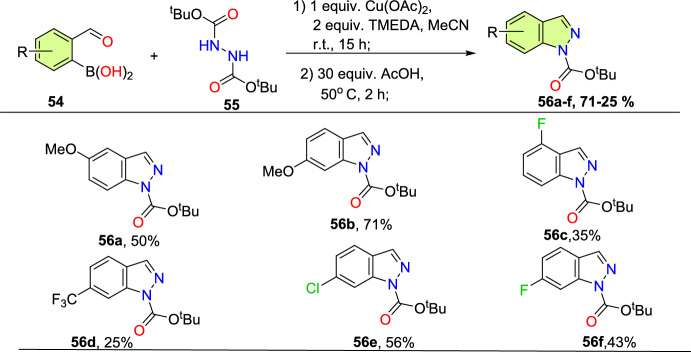
Scope of boronic acids **54** in the reaction with diazadicarboxylate tert-butyl-2-acetylhydrazine-1-carboxylate (**55**)

Initially, various 2-formyl boronic acids (**54**) reacted with di-tert-butyl hydrazine-1,2-dicarboxylate (**55**), affording target indazoles **56a–f** in a one-pot, two-step synthesis. AcOH was identified as an appropriate acid for the cyclization step to furnish the analogous products (Scheme 55). 2-Formylboronic acids with an electron-donating –OMe unit at the fourth and fifth position reacted with diisopropyl (E)-diazene-1,2-dicarboxylate (**56**) and produced the indazole products **56a** and **56b** in 50% and 71% yields respectively. Similarly, the 2-formyl boronic acids with electron-withdrawing 3-F, 5-CF_3_, 5-Cl, and 5-F units gave the indazole motifs **56c–f** in moderate to low yields.

**Scheme 56:**
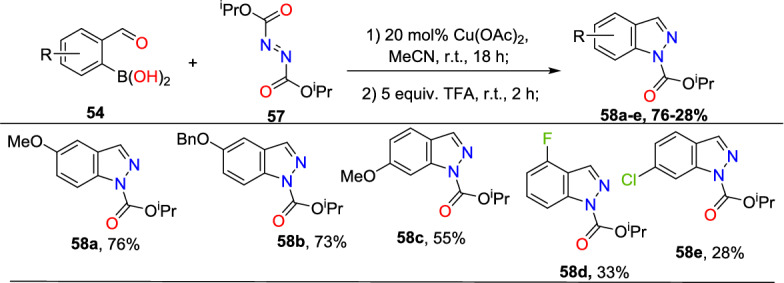
Scope of boronic acid **54** in the reaction with diisopropyl (E)-diazene-1,2-dicarboxylate(**57**)

Next, diverse hydrazine dicarboxylates such as tert-butyl-2-acetylhydrazine-1-carboxylate, diisopropyl (E)-diazene-1,2-dicarboxylate, and dibenzyl (E)-diazene-1,2-dicarboxylate were investigated for the compatibility for reaction with substituted 2-formyl boric acid (Scheme56). However, the best yield of indazole product was with tert-butyl-2-acetylhydrazine-1-carboxylate (**57**), and trifluoroacetic acid (TFA) was identified as an appropriate acid for the cyclization process to provide the consequent products **58a–e**. 2-Formyl boronic acids with electron-donating 4–OMe, 4-OBn, and 5-OMe units gave the indazole motifs **58a–c** in 76–55% yields. Similarly, the formyl boronic acids with electron-withdrawing 3-F and 5-Cl units gave the target indazole products **58d** and **58e** in comparatively low yields.

**Scheme 57:**
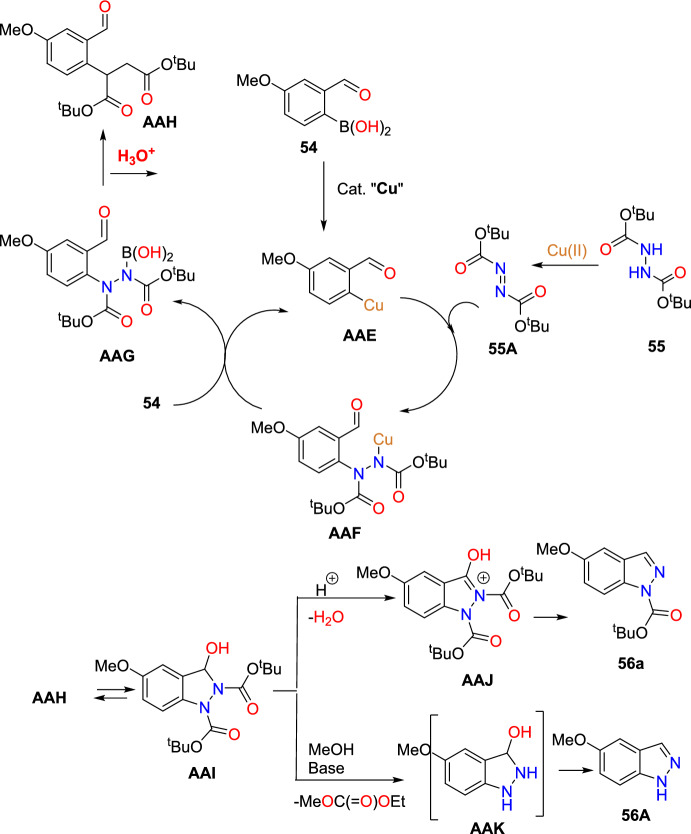
Proposed mechanism for 1*N*-alkoxycarbonyl indazole

Scheme 57 presents the mechanism for the formation of the C–N bond in the Cu-promoted reaction of aryl boronic acids with diazadicarboxylates. The formation of an aryl copper species **AAE** was suggested from aryl boronic acid **54** on transmetalation reaction with a Cu catalyst. The addition of intermediate **AAE** to the N=N double bond gives an arylhydrazine **AAF** which undergoes the transmetalation with boronic acid **54** to give intermediate **AAG** and return aryl copper species **AAE** into the catalytic cycle. Work-up would produce arylhydrazine **AAH**.

Uemura and Chatani [96] report that dialkoxycarbonylhydrazines are not competent substrates for this reaction unless an additional oxidant is added. According to this, a stoichiometric amount of copper oxidizes hydrazine **55** to diazadicarboxylate **55A**. Scheme 57 provides the predicted mechanism for the condensation of the arylhydrazine intermediate into indazole. *N*-acyliminium ion **AAJ** was formed from intermediate **AAI** in the presence of acid. One ethoxycarbonyl group in intermediate **AAJ** is selectively hydrolyzed to yield 1N-ethoxycarbonyl indazole **56a**. Instead, under a basic environment, both ethoxy carbonyl groups are broken down to generate the intermediate **AAK**, which on dehydration, produces the indazole **56A**. In turn, the basic condition enables the cleavage of both ethoxycarbonyl groups, leading to intermediate **AAK**, which eliminates water to give indazole **56A**.

### Synthesis of Substituted Indazole Acetic Acids by N–N Bond Formation Reaction

**Scheme 58:**
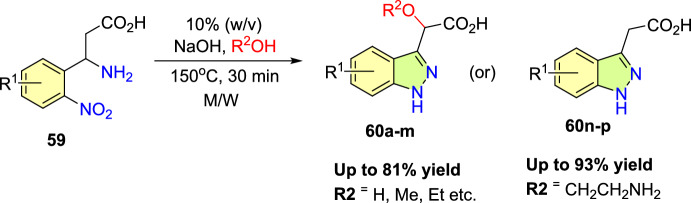
Base-catalyzed cyclization of 2-nitrobenzylamines

Luke group reported novel cascade N–N bond formation reactions [97] towards the practical construction of three different indazole acetic acid motifs (unsubstituted, hydroxy, and alkoxy) by heating 3-amino-3-(2-nitroaryl)propanoic acids with an appropriate nucleophile/solvent under basic conditions, as presented in Scheme 58. In this work, two different heterocyclization reactions were reported, one of which was the conversion of 3-amino-3-(2-nitroaryl)propanoic acids into 1*H*-indazoles in the presence of NaOH and various alcohols. By using simple alcohols as solvents, the reaction of 3-amino-3-(2-nitroaryl)propanoic acids with ethanolamine and NaOH resulted in the exclusive formation of 2-indazole acetic acids without incorporation of the corresponding alkoxy group.

A pilot screening of reaction conditions shows that low concentrations of base or lower temperatures are directed to either incomplete consumption of **59** or poor conversion to the target products. A variety of alkoxyindazole acetic acids (**60a–n**) were produced by microwave irradiation of **59**, in particular alcohols at a high temperature, i.e., 150 °C. From the experimental screening, both methanol and ethanol were recognized as effective solvents, producing superior yields of the desired products (**60a** and **60c**), and a reduction of yields was observed when moving to higher alcohols, with longer chain lengths (compare **60c–e**). Pleasantly, alcohol **60b** was obtained in good yield by using water as an unhindered nucleophile. The isobutanol (2° alcohol) provided the respective indazole carboxylic acid **60h** in low yield, whereas the reaction with isopropanol was unproductive, which might be due to repeated over-pressurization events. The reaction involving allyl or benzyl alcohol resulted only in complex reaction mixtures, and the trifluoroethanol reaction only produced a trace amount of product. It is noteworthy that an extra polar group like alcohol or a methoxy group was well tolerated, generating the desired alkoxy products **60f** and **60g** in good yields (Scheme 58).

**Scheme 59:**
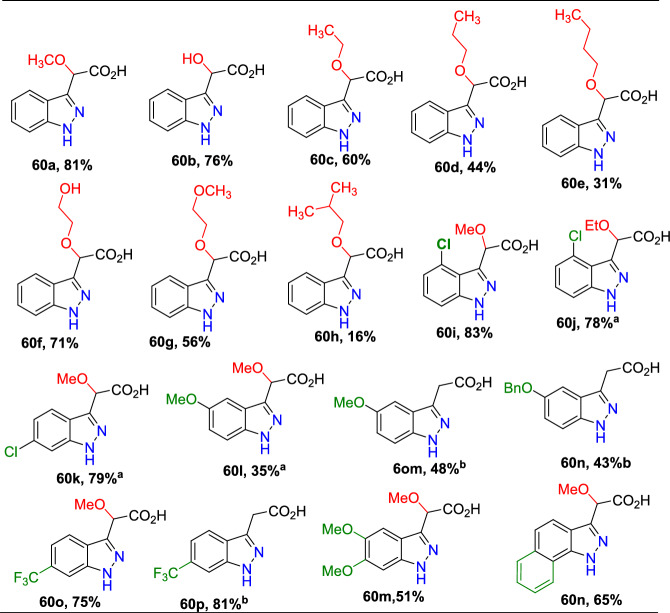
Microwave-assisted synthesis of hydroxy- and alkoxyindazole acetic acids (**60a–h**)

Further, the influence of substituents on an aromatic ring for cyclization of 3-amino-3-(2-nitroaryl)propanoic acids utilizing the high-yielding alcohols ethanol, methanol, and ethanolamine were examined (Scheme 59). The presence of a chlorine atom at the fourth 4-position of the starting material did not show a perceptible effect on the overall outcome of the reaction, and the products **60i** and **60j** are obtained in high yields. Conversely, the introduction of an electron-donating −OMe or −OBn group in the 5-position is responsible for the noticeable reduction in yields of products **60l–n**. The presence of electron-withdrawing –Cl (**60 k)** or –CF_3_ groups (**60o)** at the 6-position created the target compounds in good yields. The introduction of di-OMe groups at the 5- and 6-positions gave the target product **60m** in 51% yield. In addition, the benzo-fused analogue **60n** was obtained in good yields by the cyclization of the equivalent naphthyl starting material. It was also reported that if water was used as the nucleophile with 3-amino-3-(3-methoxy-2-nitrophenyl)propanoic acid, even though the full conversion was attained, the product could not be isolated, as it was unstable.

**Scheme 60.**
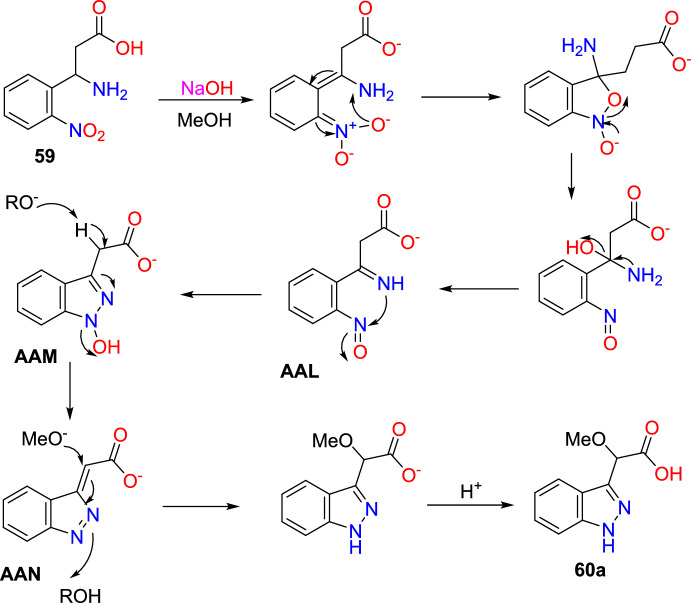
Plausible mechanism for alkoxyindazole acetic acid **60a**

The predicted mechanism is presented in Scheme 60. The nitroso intermediate **AAL** was produced by the initial oxygen transfer and elimination, which on consequent intramolecular capture by the pendent imino group, gave the indazole motif **AAM**. Next, the vinylogous imine derivative **AAN** is then produced by enolization and hydroxide elimination. Finally, the alkoxide nucleophile is then added to this intermediate **AAN**, followed by acidic workup to produce the indazole methoxyacetic acid product **60a**.

### Through Aone-Pot Sequential Strategy

**Scheme 61:**
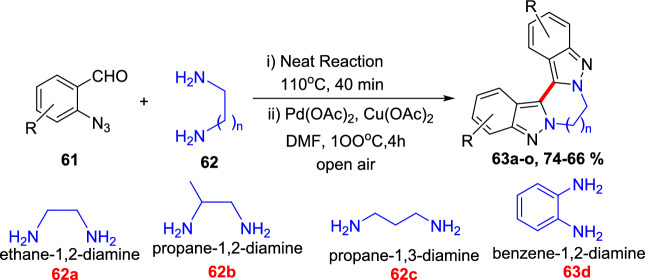
Synthetic route for bis-indazoles

Kishan’s group described an efficient one-pot sequential strategy for the preparation of fused bis-indazoles/indazoles starting from amines and *o*-azido aldehydes (Scheme 61) [98]. The formation of 2*H*-indazole achieved the total transformation by the Pd-catalyzed intramolecular cross-dehydrogenative coupling reaction of *o*-azidoaldehydes and amines. The reaction sequence resulted in the development of three heterocyclic rings via the formation of five new bonds.

**Scheme 62:**
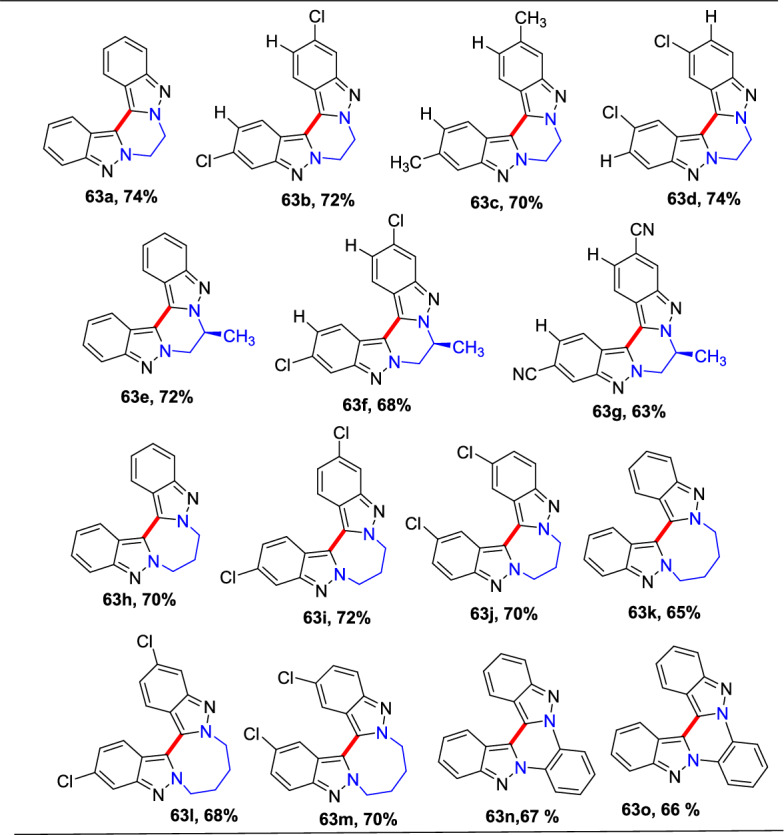
Substrate scope of the one-pot sequential cascade reaction of annulated bis-indazoles

The described protocol features reduced synthetic steps and wide functional group tolerance with good product yields. Under the optimized conditions, the authors tested the sequential cascade protocol with different *o*-azido aldehydes (**61**) and diamine derivatives (**62**), and Scheme 62 summarizes the results. The authors examined the scope and limitations of diamine substrates such as ethane-1,2-diamine (**62a**) and propane-1,2-diamine (**62b**) with different *o*-azido aldehydes (**61**). Initially, the target products **63a–d** were achieved in superior yields by the reaction of ethane-1,2-diamine (**62a**) in ethane with azido aldehyde (**61**) containing –Cl and –CH_3_ derivatives.

The scope of the protocol with diverse diamines were tested, and the reaction of propane-1,2-diamine (**62b**) with azido aldehyde (**61**) and substituted azido aldehyde with −Cl and −CN produced the desired products **63e–g** in good yields. A series of diamines acquiescent to ethane-1,2-diamine were not limited to piperazine derivatives. The three- or four-carbon diamines (**2**), like propane-1,3-diamine (**62c**) and butane-1,4-diamine (**62**), show good compatibility for this reaction, providing the consecutive diazepine indazoles or diazocine indazoles **63h–k** and **63l–m** in good yields (66–72%). In addition, the aromatic diamines, such as benzene-1,2-diamine (**62d**) and 4,5-dimethylbenzene-1,2-diamine (**62**), gave acceptable yields of the indazole product **63n** and **63o**.

**Scheme 63:**
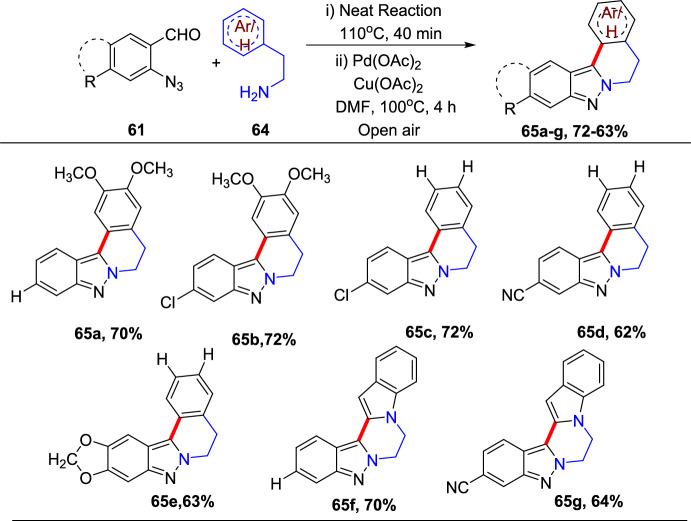
Substrate scope of the one-pot sequential cascade reaction of annulated indazoles

The adaptability of this sequential one-pot annulations strategy further extended to the construction of annulated bis-indazoles. Under the standard reaction conditions, the protocol was effectively extended to annulated indazoles using aryl/hetero aryl benzyl amines **64a–c** (Scheme 63) to produce the analogous annulated indazoles **65a–g** in fine yields. *o*-Azido aldehydes and 2-azido-4-chlorobenzaldehyde easily coordinated with dimethoxyphenylethylamine (**64a**) to afford the consecutive products **65a** and **65b** in 70% and 72% yields, respectively. Moreover, the developed chemistry is applied for the construction of a vital group of heterocyclic compounds. *o*-Azido aldehydes having −Cl, −CN, and −O–CH_2_–O− derivatives afforded the respective indazole products **65c–e** in good yields. Finally, these sequential cascade conditions were well applied for the reaction of 2-indol-1-ylethanamine with *o*-azido aldehydes to produce the consecutive products **65f** and **65g** in excellent yields.

**Scheme 64:**
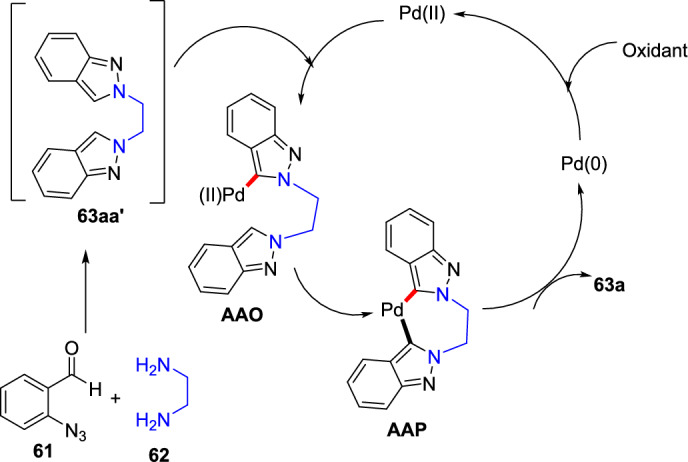
Proposed mechanism for the one-pot sequential cascade reaction

A proposed mechanism for the reported protocol is presented in Scheme 64. Initially, the *o*-azido aldehyde (**61**) reacts with diamine (**62)** to produce the isolatable 1,2-di(2*H*-indazol-2-yl)ethane intermediate **63aa′**. In the next stage, the electrophilic palladation at the C3-position of one of the indazoles affords the intermediate **AAO**, which on further electrophilic palladation at the C3-position of another indazole gave the respective seven-membered palladacycle intermediate **AAP**. Next, the intermediate **B** on subsequent reductive elimination generates the final product **63a**, and the regeneration of the catalyst takes place in the presence of an oxidant.

### Ultrasound-Promoted Heteropoly Acid-Catalyzed Synthesis

**Scheme 65:**
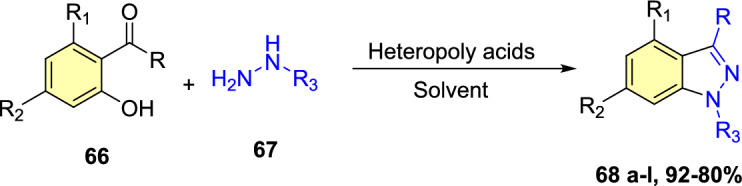
Synthesis of indazole using conventional and ultrasound sonication method using H_3_PMo_12_O_40_ and H_3_PW_12_O_40_

Manohar’s group established the metal-catalyzed condensation reaction between substituted 2-hydroxy benzaldehydes/acetophenones (**66**) and hydrazine hydrate/phenylhydrazine (**67**) for the synthesis of substituted indazoles using heteropoly acids containing tungsten and molybdenum under conventional and ultrasound sonication method (Scheme 65) [99]. Two different heteropolyacids, H_3_PMo_12_O_40_ and H_3_PW_12_O_40_, were used and identified as outstanding catalysts for the preparation of targeted indazoles (**68a–l**) in conventional and ultrasound sonication methods. However, compared to the traditional method, the ultrasound-promoted process was identified as much more potent, with increased yields of the synthesized products from 92 to 95% using H_3_PW_12_O_40_ heteropoly acid catalyst.

**Scheme 66:**
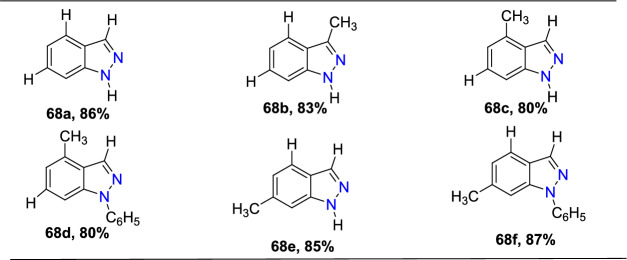
Ultrasound method for preparation of indazole derivatives (**68a–f)** by using H_3_PMo_12_O_40_

Because of their oxidizing and acidic properties, many hetero-poly acids will be employed in organic synthesis of heterocyclic compounds. Initially, ultrasound sonication of various 2-hydroxy benzaldehydes/acetophenones (**66**) reacted with hydrazine hydrate/phenylhydrazine (**67**) on heteropoly acid H_3_PMo_12_O_40_-catalyzed condensation afforded the indazole products **68a–f** in 87–80% yields (Scheme 66).

**Scheme 67:**
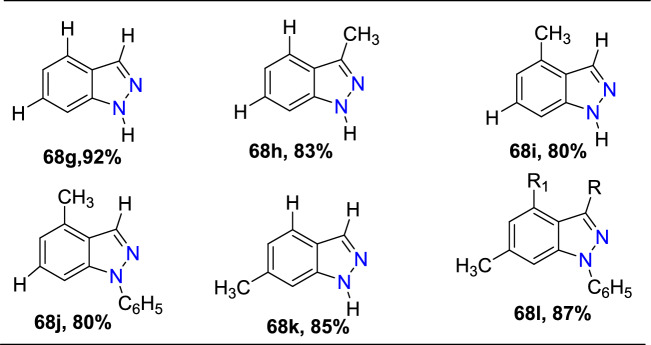
Ultrasound method for preparation of indazole derivatives **63g–l** by using H_3_PW_12_O_40_

The scope of the protocol was further examined with the same 2-hydroxy benzaldehydes/acetophenones and hydrazine hydrate/phenylhydrazine on heteropoly acid H_3_PMo_12_O_40_-catalyzed ultrasound-promoted condensation, which gave the target indazole products **68g–l** in 80–92% yields (Scheme 67).

**Scheme 68:**
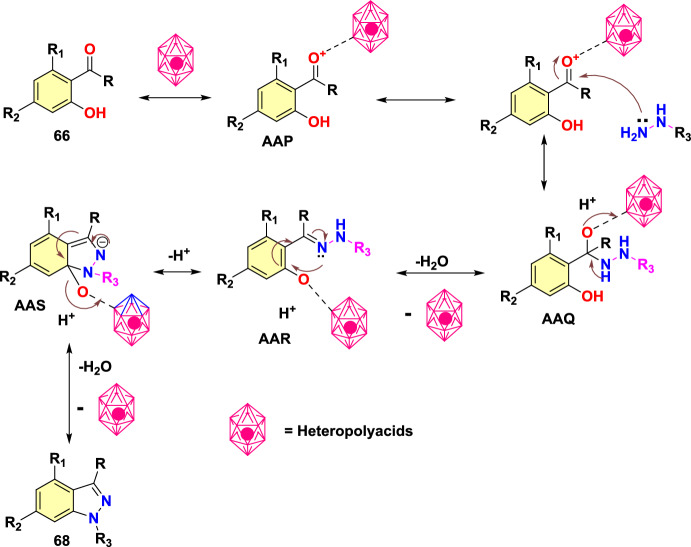
Possible mechanism using H_3_PMo_12_O_40_ and H_3_PW_12_O_40_ for synthesis of **68a–l**

A plausible reaction mechanism for the synthesis of **63a–l** using heteropoly acid is represented in Scheme 68. Initially, the metal atom of the heteropolyacid coordinates with the carbonyl oxygen of 2-hydroxy benzaldehyde/acetophenone to form the complex **AA** and make the carbonyl carbon more electrophilic. Then, the hydrazine (**67**) was added to this electrophilic carbon by donating a pair of electrons and forming the intermediate **AAQ.** Next, the migration of heteropoly acid from the carbonyl oxygen to hydroxyl oxygen and dehydration afforded the intermediate **AAR.** Next, the intermediate **AAR** on deprotonation and cyclization reactions afforded the intermediate **AAS,** which on the elimination of heteropolyacid and water molecules to complete the catalytic cycle, produced the target indazole scaffold **68.**

### Ultrasound-Assisted Synthesis by Cadogan’s Cyclization

**Scheme 69:**
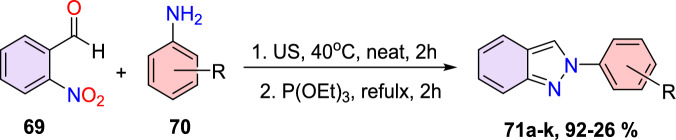
Ultrasound-assisted synthesis of 2-phenyl-2*H*-indazole derivatives

An efficient one-pot synthesis of 2-phenyl-2*H*-indazole scaffolds was reported by Karen et al. [100] that combines Cadogan’s cyclization with ultrasonic-assisted synthesis in clean conditions (Scheme 69). Next, they evaluated their anti-protozoal activity. The results of the biological assays showed that the 2-phenyl ring with electron-withdrawing groups is advantageous for antiprotozoal action. This process combines Cadogan’s cyclization, which involves refluxing the Schiff base with triethyl phosphate with Crawford ultrasound synthesis performed under controlled conditions. This method needs less purification, solvents, and workup time.

**Scheme 70:**
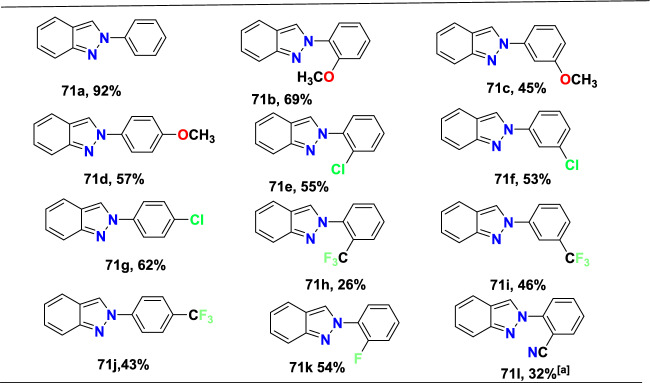
Substrate scope for 2-phenyl-2*H*-indazoles **71a-l**.^a^ The reaction mixture was ultrasonicated for four cycles for 1 h at 40 °C

The optimization was performed using 2-nitrobenzaldehyde (**69**) and aniline (**70a**) as model substrates. At the ideal optimized conditions (shown in Scheme 68), the reaction afforded the target indazole **71a** in excellent yield. The substrate scope for this reported method was explored by taking diversely substituted anilines (Scheme 70). Anilines having an electron-donating –OMe unit at the *ortho*-, *meta-*, and *para*-positions of the aniline afforded the indazole scaffolds **71b–d** in 69–45% yields. Next, the electron-withdrawing 2-Cl, 3-Cl, and 4-Cl substituted anilines gave the indazole products **71e–g** in 55–62% yields. Similarly, anilines with 2-CF_3_, 3-CF_3_, and 4-CF_3_ substituents produced the target indazoles **71h–j** in moderate yields. Moreover, the anilines with 2-F and 2-CN units produced the corresponding products **71k** and **71l** in 54% and 32% yields, respectively. Anilines with similar substituents at 2- or 3-positions of the phenyl ring are also obtained. Aniline with the 2-CN group at the 2-position gave the consecutive indazole derivative in 32% yield by ultrasonicating the reaction mixture for four cycles for 1 h at 40 °C (see below). This method showed better results for the production of target indazoles in better yields than the previously reported conventional methods.

### Ultrasonic-Promoted Synthesis

**Scheme 71:**
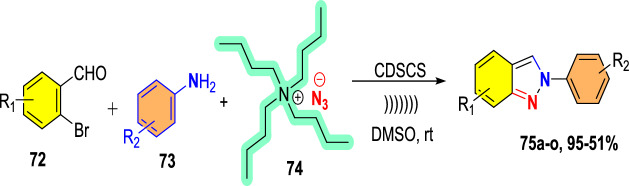
Ultrasonic-promoted synthesis of 2-aryl indazoles**75a–o**

Soltani Rad’s group presented an ultrasonic-based one-pot, three-component reaction of 2-bromo benzaldehyde (**72**), primary amine (**73**), and tetra-butyl ammonium azide (TBAA) (**74**) in the presence of Cu-doped silica cuprous sulphate (CDSCS) to synthesize 2-aryl indazoles (**75a–o**) with superior yields at room temperature (Scheme 71) [101]. The method proceeds through successive condensation, with C–N and N–N bonds created under ultrasonic irradiation employing TBAA as an azide (nitrogen) source and CDSCS as a heterogeneous nano-catalyst. Advantages of this method include low catalyst loading, superior product yields, readily available precursors, moderate conditions, reduced reaction times, simplicity in operation and separation, no use of auxiliary ligand, and reduced by-products and chemical reactions, thus providing a reusable catalyst. This ultrasonic irradiation technique reflects remarkable advances in terms of superior yields and reduced reaction times in contrast to the conventional heating method in current research.

After investigating the ideal reaction conditions via optimization, the authors screened the versatility and the scope of this protocol to other 2-bromo benzaldehyde and primary amine derivatives (Table 1). Further, they also carried out the preparation of 2*H*-indazoles exploiting CDSCS as a catalyst under conventional heating practice (DMSO at 120 °C) and compared the results with ultrasonic-mediated protocols. Dramatic improvements in terms of higher yields and shorter reaction times were observed compared with the conventional heating method. Under ultrasonic conditions, in most cases, the reaction times are shortened, and yields are enhanced. Therefore, the results in Table 1 confirm the advantage of the ultrasonic method over the conventional thermal method.

**Table 1:**
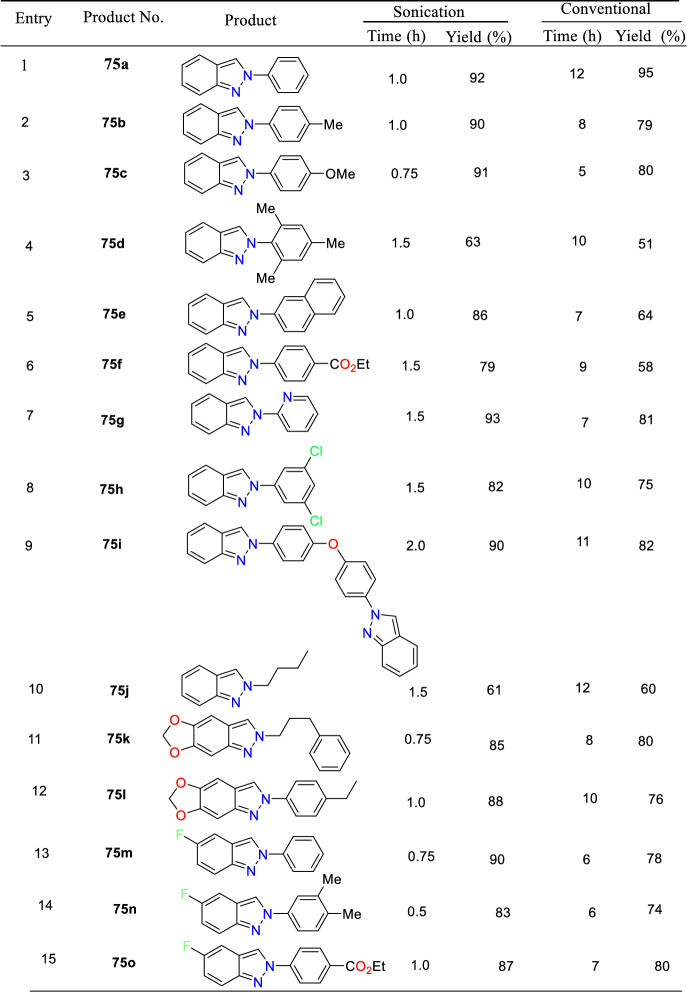
The synthesized 2*H*- indazoles using CDSCS under ultrasonic irradiation

This ultrasonic-promoted protocol works well with structurally diverse aliphatic, aromatic, and heteroaromatic amines and tolerates different functional groups to achieve the consequent 2*H*-indazoles **75a-o** in superior yields. The 2-bromo benzaldehyde combined with aniline and TBAA in the company of CDSCS at room temperature afforded the target indazole **75a** in excellent 92% yield. Next, anilines with electron-donating 4–Me, 4-OMe, 2,4,6-triMe, 4-Et, and 3,4-diMe substituents were efficiently applied in the current protocol to afford the consecutive products **75b–c**, **75l,** and **75n** in excellent yields (Table 1, entries 2, 3, 12, and 14). The sterically crowded amines, such as 2,4,6-trimethyl benzenamine, were successfully converted to 2*H*-indazole **75d** in 63% yield (Table 1, entry 4). The naphthalen-1-amine and heteroaromatic amines, such as 2-aminopyridine, efficaciously afforded the respective indazoles **75e** and **75g** in 86% and 92% yields (Table 1, entries 5 and 7). Anilines with electron-deficient groups such as −CO_2_Et provided the corresponding products **75f** and **75o** in 79 and 87% yields, respectively (Table 1, entries 6 and 15). The reaction of 4,4′-oxy dianiline with two equivalents of 2-bromo benzaldehyde afforded **75i** in an excellent 90% yield (Table 1, entry 9). Moreover, under standard reaction conditions, aliphatic amines such as n-butylamine and 3-phenylpropan-1-amine were readily reacted with 2-bromoaldehyde to produce the favorable yields of indazoles (**75j** and **75k**) (Table 1, entries 10 and 11, respectively). Furthermore, 6-bromobenzo[*d*]-[1,3]dioxole-5-carbaldehyde and 2-bromo-5-fluoro benzaldehyde also show good compatibility towards this method (Table 1, entries 11–15).

In this protocol, in addition to the ultrasonic irradiation and catalytic performance of CDSCS, which influence the reaction, TBAA not only behaves as an azide source but also can extensively promote the reaction because of its phase transfer catalytic (PTC) nature. The recoverability and reusability of heterogeneous catalysts are the essential benefits and advantages over homogeneous catalysts from both environmental and economic points of view. The experimental results also confirmed that CDSCS is a recyclable and reusable catalyst without any significant loss of its activity.

**Scheme 72:**
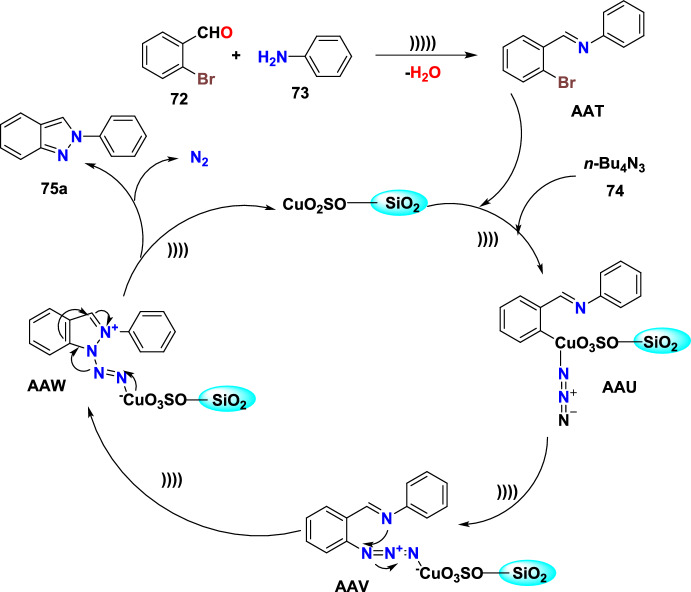
A plausible mechanism for the construction of 2-phenyl-2*H*-indazole **75a**

A plausible mechanism was suggested for the preparation of 2*H*-indazole derivatives via a single-pot three-component reaction of 2-bromo benzaldehydes, amines, and TBAA using CDSCS under ultrasonic irradiation (Scheme 72). According to this mechanism, successive condensation, and C-N and N-N bond formation result in the generation of 2*H*-indazoles. Scheme 72 illustrates the mechanism for the synthesis of 2-phenyl-2*H*-indazole as a sample compound. Initially, the reaction occurs through the condensation of 2-bromo benzaldehyde with aniline to generate the imine (**AAT**) as the primary intermediate. The in situ generation of imine was confirmed at the early stage of the reaction, which can be evidenced by thin-layer chromatography (TLC) or gas chromatography (GC) techniques through the comparison with pre-synthesized imine or provided authentic sample. In continuation, TBAA was reacted with imine (**AAT**) in the presence of CDSCS to afford the intermediate (**AAU**), followed by reductive elimination to the azide–copper complex (**AAV**) through C–N bond formation. Afterwards, NAN bond formation via the intramolecular cyclization of (**AAV**) resulted in intermediate (**AAW**). Eventually, the elimination of N_2_ gas and dissociation of the catalyst led to 2*H*-indazole (**75a**) as the ultimate product.

### Metal-Free Photochemical or Thermochemical Processes

**Scheme 73:**
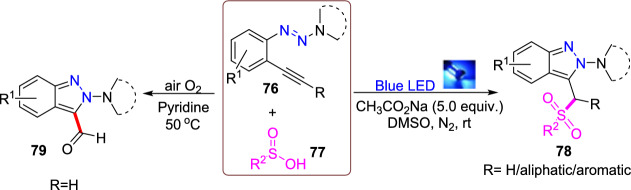
Metal-free synthesis of 2*H*-indazoles in photochemical/thermal conditions

Zhang et al. described an efficient, easy, metal-free synthesis of a 2*H*-indazole skeleton both under photochemical and thermal conditions (Scheme 73) [102]. The reaction comprised 2((aryl/alkyl/H)ethynyl)) aryl triazenes with aryl sulfinic acids to obtain the targeted 3-functionalized 2*H*-indazoles without a photosensitizer via an electron donor–acceptor complex under visible light irradiation at room temperature, without extra photocatalyst. Under thermal conditions, at 50 °C in air, 2*H*-indazole-3-carbaldehydes were obtained via an intramolecular oxidation/cyclization of 2-(ethynyl)aryl triazenes in the presence of arylsulfinic acid. Simple starting materials and kinder, metal-free reaction conditions are the benefits of this approach over traditional methods for synthesizing 2*H*-indazoles.

**Scheme 74:**
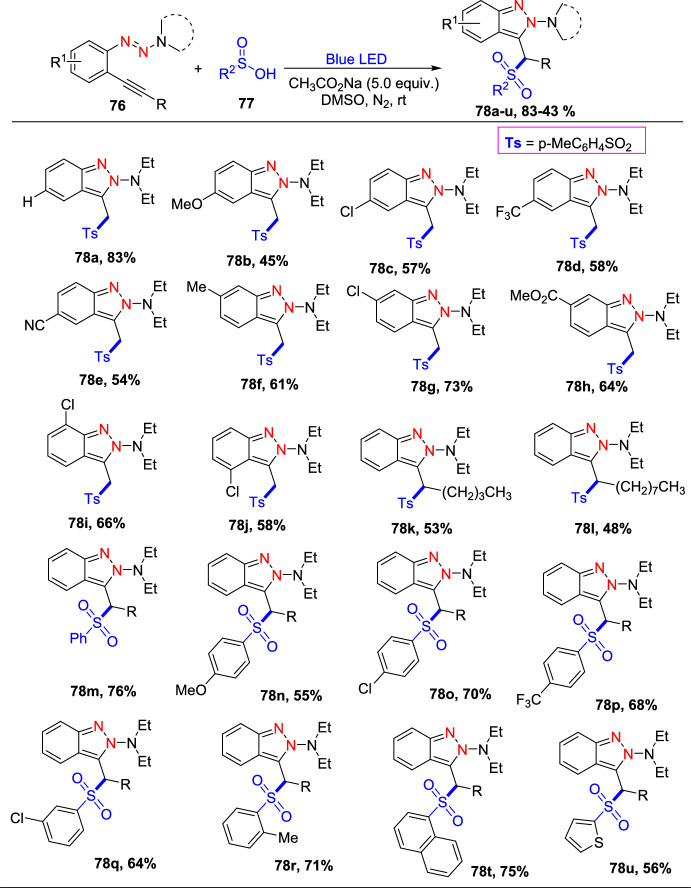
Synthesis of *N*,*N*-diethyl-3-(sufonyl)-2*H*-indazol-2-amines

The standard reaction conditions using (*E*)-3,3-diethyl-1-(2-ethynylphenyl)triazene (**76**) with 4-methylbenzene sulfinic acid (**77**) as model substrates uses CH_3_CO_2_Na as a base, in dimethyl sulfoxide (DMSO) under the irradiation of a blue light-emitting diode (LED, 410−415 nm) without additional photocatalyst under nitrogen atmosphere for 10 h. The reaction afforded the cyclization product **78a** in 83% yield. Among the wavelengths of the LEDs examined, 420−425 nm showed the highest reactivity. The authors explored the scope of the protocol for the reactions of various triazenes and aryl sulfinic acids, and Scheme 74 summarizes the outcomes. The aryl triazene bearing electron-donating −MeO and electron-withdrawing −Cl, −CF_3_, and −CN units at the C4-positions of the aromatic rings reacted smoothly with sulfinic acid to afford the consecutive products **78b** and **78c–e** in 45% and 54–58% yields, respectively. Aryl triazenes having electron-withdrawing substituents (−Me, −Cl, −CO_2_Me) at the C3-positions of the aryl rings also showed good tolerance to provide the target products **78f–h** in 61−73% yields. Moreover, the indazole motifs **78i** and **78j** were prepared in respective 66% and 58% yields using aryl triazenes with 2-Cl and 5-Cl groups. The triazenes with aromatic/aliphatic internal alkynes were used to obtain the consecutive products **78k** and **78l** in 53% and 48% yield, respectively.

The scope with diverse sulfinic acids (**77**) was explored, and phenyl sulfinic acid with diverse substitutions, such as *p-*MeOC_6_H_4_, *p*-ClC_6_H_4_, and *p*-CF_3_C_6_H_4_ at the C4-position, reacted with (*E*)-3,3-diethyl-1-(2-ethynylphenyl)triaz-1-ene (**76**) and afforded the respective indazole products **78n–p** in 55–70% yields. Similarly, phenylsulfinic acids with *m*-Cl and *o*-Me units exhibit good compatibility in the reaction with **76** under standard reaction conditions to obtain the products **78q** and **78r** in 64% and 71% yields, respectively. The target products **78t** and **78u** were attained in superior yields (75%) from the reaction of naphthalene-1-sulfinic acid with (**76**). Moreover, the thiophene-2-sulfinic acid produced the respective indazole product **78u** in 56% yield on reaction with (*E*)-3,3-diethyl-1-(2-ethynylphenyl)triaz-1-ene (**76**).

**Scheme 75:**
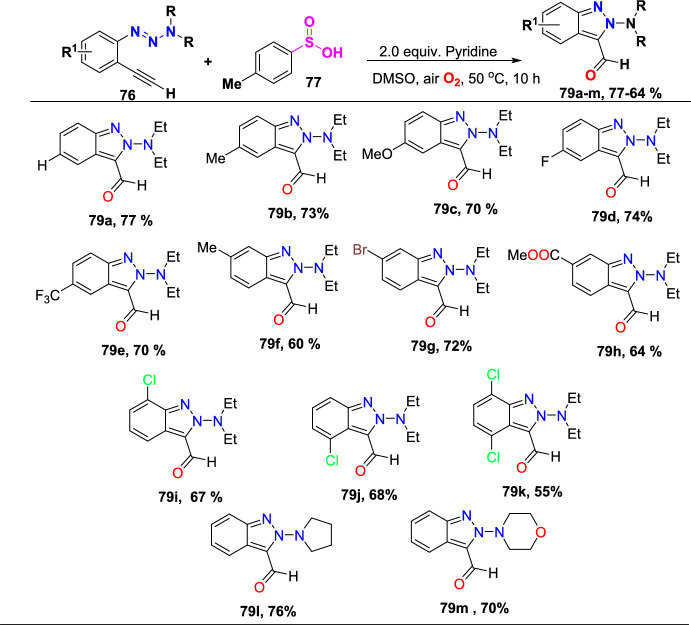
Synthesis of 2*H*-indazole-3-carbaldehydes

The authors additionally extended their work towards the construction of diverse 2*H*-indazole-3-carbaldehydes (**79**) from arylsulfinic acid (**77**) and 2-(ethynyl)aryltriazenes (**76a**) via an intramolecular oxidation/cyclization at 50 °C in air atmosphere (Scheme 75). The (E)-3,3-diethyl-1-(2-ethynylphenyl)triaz-1-ene (**76a**) reacts with paratoulenesulfinic acid (PTSA) in the presence of pyridine base in DMSO at 50 °C to afford the target indazole **79a** in 77% yield. Under similar conditions, various substrates **76** with 4-Me, 4-OMe, 4-F, 4-CF_3_, and 4-CN substituents produced the desired products **79b–f** in 60–74% yields. Compound **76** with a substituent 3-Me, 3- Br, and 3-COOMe at the C3-position of the phenyl ring underwent an intramolecular cyclization smoothly to generate the desired products **79g–i** in 60%–72% yields. In addition, substrates **76** with a 2-Cl, 5-Cl, and 2,5-diCl substituent on the phenyl rings were effective, leading to the corresponding products **79j**, **79k**, and **79l** in 67%, 68%, and 55% yields, respectively. Notably, replacement of the *N*,*N*-diethyl groups in **76** with other groups, such as *N*-pyrrolidinyl and *N*-morpholinoyl, was also compatible for this reaction to afford good yields of consecutive anticipated indazole products **79m** and **79n** in 76% and 70% yields, respectively.

**Scheme 76:**
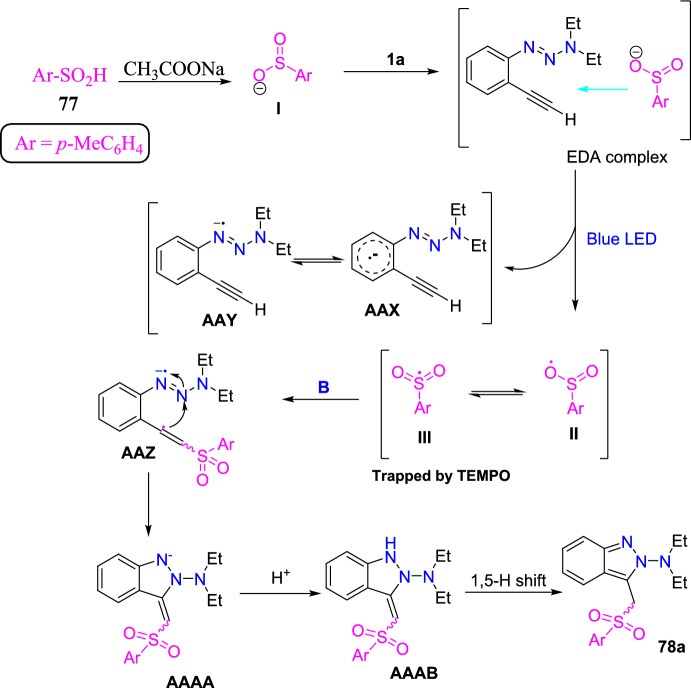
Plausible mechanism for 3-((arylsulfonyl)methyl)-*N*,*N*-diethyl-2*H*-indazol-2-amine

Scheme 76 describes the possible mechanistic pathway under visible light irradiation conditions; initially, an anion species **I** was produced from arylsulfinic acid (**77**) in the presence of CH_3_CO_2_Na. The anion species **I** reacts with substrate **76a** to generate an EDA complex, which on dissociation under visible light radiation gave the **AAX** and **II**. The dissociation of **AAX** or **AAY** produced the intermediate **II** or **III**, respectively. On free-radical transfer with nitrogen, anion radical **AAY** was transformed into the intermediate **AAZ**. A cyclized intermediate **AAAA** was produced from intermediate **AAZ** through a new C−N bond formation. Finally, the intermediate **AAAA** on protonation and 1,5-H shift delivers the final product **78a**. Radical III cannot effectively attack neutral triazenes in the EDA complex by the anion **I** and **76a**. Thus, intermediate III can more effectively form intermediate **AAZ** with radical anion B.

**Scheme 77:**
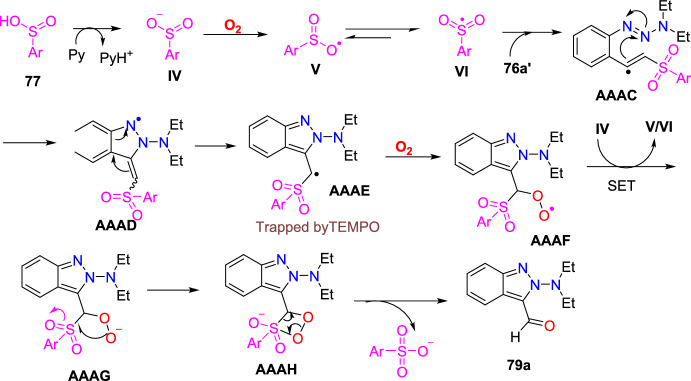
Possible mechanism2-(diethylamino)-2*H*-indazole-3-carbaldehyde **79a**

In addition, Scheme 77 details the possible mechanism proposed for the thermochemical conversion of 2-(ethynyl)aryltriazenes **76a′** to 2-(diethylamino)-2*H*-indazole-3-carbaldehyde (**79a**) in the presence of arylsulfinic acid. Arylsulfinic acid **77**, on deprotonation with pyridine base, produces the aryl sulfonic acid anion **IV**, which on subsequent electron transfer afforded the free radical V that, on rearrangement, gives a thio radical **V**. Next, the C−H bond of **76a′** attacked by **III** provides the carbon radical intermediate **AAAC** that undergoes intramolecular C−N bond formation reaction to afford the nitrogen-free radical intermediate **AAAD**. Through a radical transfer reaction, the carbon radical **AAAE** was obtained from the **AAAD** intermediate, which, on oxidation with molecular oxygen, gives a peroxy radical **AAAF**. Later, the peroxy anion intermediate **AAAG** was obtained along with the generation of **V** or **VI** through a single-electron transfer (SET) of **AAAF** by the aryl sulfinic acid anion **IV**. Next, the peroxy anion in intermediate **AAAG** attacks the sulfur, thus breaking the S=O to produce the intermediate **AAAH**, which finally undergoes intramolecular elimination, affording the target indazole **79a** along with arylsulfonate.

### Transition Metal-Free Synthesis

**Scheme 78:**
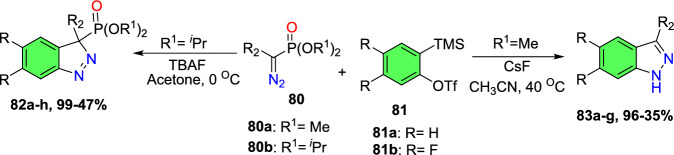
Synthetic route for 3-alkyl/aryl-3*H*-indazole-3-phosphonates and 3-alkyl/aryl-1*H*-indazoles

Peng and coworkers described the preliminary outcomes of the successful construction of 3-alkyl/aryl-3*H*-indazole-3-phosphonates and 3-alkyl/aryl-1*H*-indazoles by a 1,3-dipolar cycloaddition between substituted *α*-diazomethylphosphonates and arynes under gentle reaction conditions [103]. This procedure involved the introduction of both alkyl and aryl units at the C3-site of indazoles. The selectivity for 1*H*- and 3*H*-ndazole synthesis was regulated by the phosphoryl group (Scheme 78). This report covered the construction of 3-alkyl/aryl-3*H*-indazole-3-phosphonates, with the combination of two biologically important scaffolds, indazole and phosphoryl, in a single molecule for the first time. These hybrid indazole scaffolds were shown to be promising for the discovery of novel and lead medicinal chemistry drug motifs. This protocol is advantageous over previously reported methods, which require more steps to form the products associated with 1-aryl-1*H*-indazoles.

**Scheme 79:**
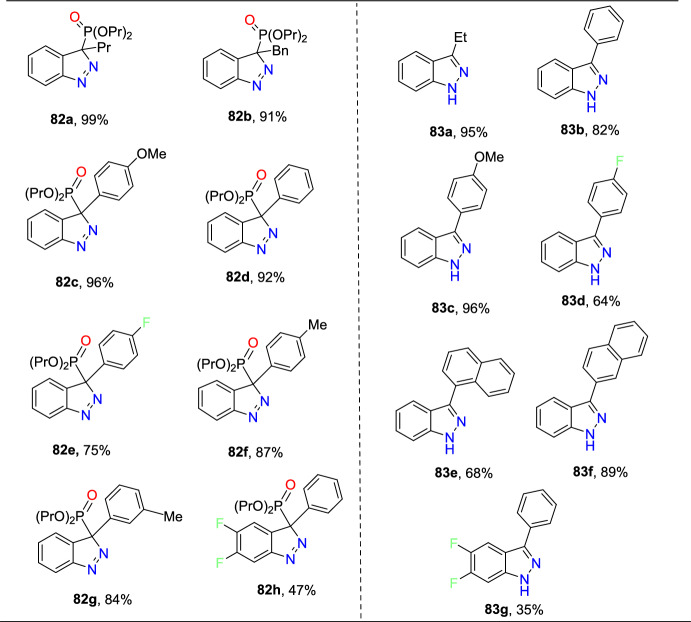
Substrate scope for 3-alkyl/aryl-3*H*-indazole-3-phosphonates and 3-alkyl/aryl-1*H*-indazoles

First, with standard reaction conditions, the authors investigated the reactions leading to diisopropyl 3-alkyl/aryl-3*H*-indazole-3-phosphonates (**82a–h**). Scheme 79 describes the results. All the diisopropyl α-diazoalkyl phosphonates (**80b**) were cyclized with benzyne generated in situ from **81a** to give the products in excellent yield. However, when diisopropyl α-diazoarylmethyl phosphonates were used, the yields varied depending on the substituents on the aromatic ring. Electron-withdrawing groups, such as F, impeded the reactions; the reaction time was prolonged, and the product **82e** was obtained in moderate yields. The diisopropyl α-diazoarylmethylphosphonate with an unsubstituted phenyl ring produced the indazole **82d** in 92% yield. An aryl ring with electron-donating 4–OMe, 4-Me, and 3-Me produced the respective indazole products **82c**, **82f**, and **82g** in 96%, 87%, and 84% yields, respectively. The di-F-substituted aryne (obtained from **81b**) underwent the 1,3-dipolar cyclization and afforded the indazole product **82h** in less (47%) yield.

**Scheme 80:**
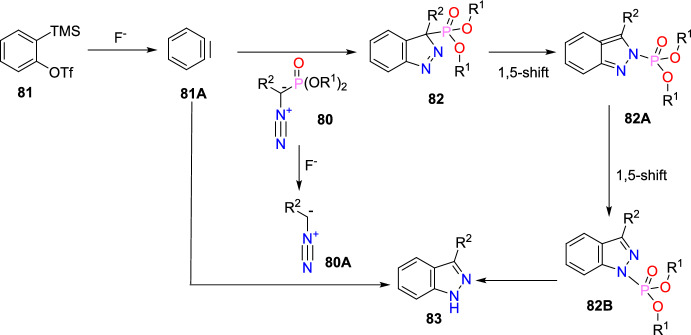
Plausible mechanism for 3-alkyl/aryl-3*H*-indazole-3-phosphonate (**82**) and 3-alkyl/aryl-1*H*-indazole (**83**)

In addition, the cyclization between different *α*-diazomethyl phosphonates (**80**) reacted smoothly with arynes produced in situ from (**81**) and afforded the 3-alkyl-1*H*-indazoles **83a–g** in modest to superior yields (Scheme 80). Dimethyl (1-diazopropyl)phosphonate (**80a**) and aryne (obtained from **81**) on 1,3-dipolar cyclization provided the target indazole **83a** in excellent yield (95%). *α*-Diazoarylmethyl phosphonates with –C_6_H_5_, 4-OMeC_6_H_4_, 4-FC_6_H_4_, 1-napthyl, and 2-napthyl substitutions also reacted smoothly with (**81**), producing the respective 3-aryl-1*H*-indazole scaffolds **83b–f** in moderate to excellent yields. Further, the aryne generated from 4,5-difluoro-2-(trimethylsilyl)phenyl trifluoro methane sulfonate underwent cyclization with dimethyl (diazo(phenyl)methyl)phosphonate to afford the target indazole **83g** in low (35%) yield.

Scheme 80 presents the proposed mechanism of the above reaction. The alkyne **81A** obtained from **81** undergoes 1,3 dipolar addition with *α*-diazophosphonate **80** to give the 3-substituted-3*H*-indazole-3-phosphonate **82**, which undergoes two 1,5-shifts to give 1*H*-indazol-1-yl phosphonate **82B**. Under the reaction condition, **82B** hydrolyzes to give 1*H*-indazole **83**. Alternatively, under the action of cesium fluoride, *α*-diazophosphonate **80** would undergo dephosphonylation to give *α*-diazo intermediate **80A**, which undergoes 1,3-dipolar addition with alkyne **81A** to give **83**. The authors considered both paths and did not ignore any possible route. With diisopropyl *α*-diazoalkylphosphonate, the bulkier isopropyl generates the diisopropylphosphonyl group out of the plane so that the orbital cannot react effectively with that of N-2, which makes the 1,5-shift unavailable.

### By Diazo Activation Strategy

**Scheme 81:**
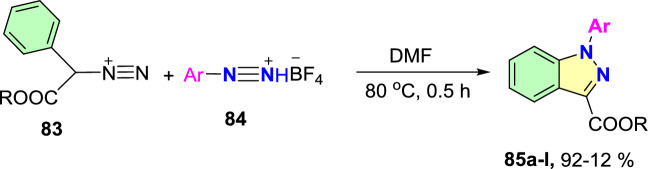
Synthesis of indazoles **85a–l** by diazo activation strategy

Shi and co-workers presented a catalyst and reagent-free donor/acceptor diazo activation approach that progressed by means of condensation with diazonium salts (**84**) for the first time [104]. The diazo activation by diazonium salt produced the key diazenium intermediate. This diazenium intermediate is found to undergo cyclization to form the respective indazoles in exceptional yields (Scheme 81).

**Scheme 82:**
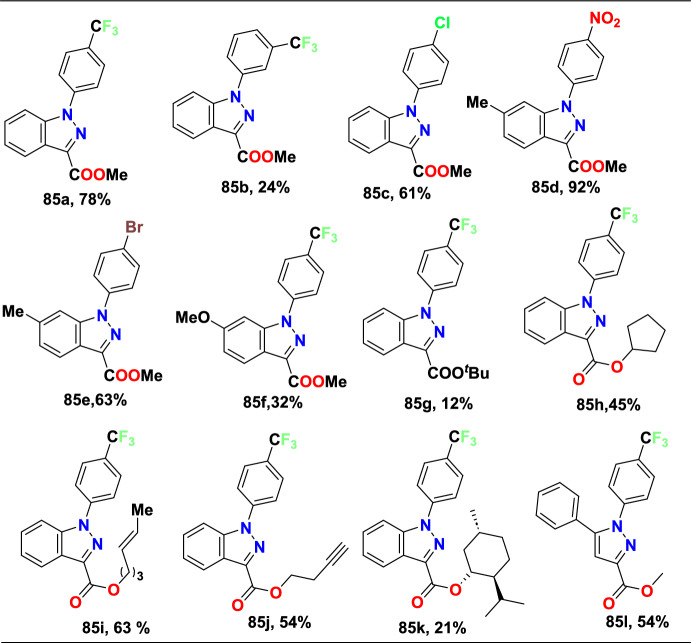
Substrate scope for methyl 1-(aryl)-1*H*-indazole-3-carboxylate

Further, the authors assessed the efficiency of the reported protocol and substrate scope (Scheme 82). Most aryl diazonium salts (**84**) containing electron-withdrawing substituents were suitable for this transformation, giving sound to excellent yields. For instance, the aryl diazonium salts containing electron-withdrawing 4-CF_3_, 3-CF_3_, and 4-Cl substituents afforded the targeted indazole products **85a–c** in 78%, 24%, and 61% yields, respectively. Notably, aryl diazonium salts with electron-donating substituents failed to produce any desired product. *Para-*methyl-substituted aryl diazo esters (**83**) reacted smoothly with aryl diazonium salts having electron-withdrawing 4-NO_2_, 4-Br, and 4-CF_3_ units, affording the cyclized products **85d–f**, respectively, in good to excellent yield. Further, they investigated the differently sized ester groups of the diazo ester constituent. Thus, altering the methyl ester to a larger −^*t*^Bu ester reduced the yield of the product indazole **85g** to only 12%. Diazoesters (**83**) with a customized ester chain enclosing functional units, such as alkene (**83h**), saturated ring system(**83i**), alkyne (**83j**), and menthol (**83k**), were well suited for this transformation. Additionally, vinyl diazoester gave the consequent *N*-arylpyrazole (**83l**) in moderate yield under metal-free conditions. If electron-withdrawing groups were present on the diazo aryl ring, no product formation was observed.

**Scheme 83:**
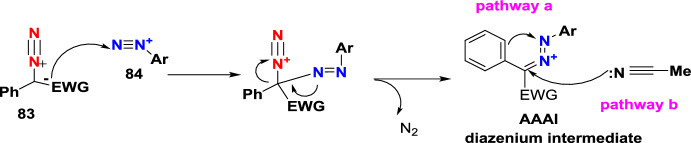
Diazo activation by diazonium and formation of diazenium intermediate

The possible mechanism for the above conversion was reported (Scheme 83). A fascinating process of reaction between the diazonium salt and diazo compound was predicted by means of nucleophilic addition of a diazo compound to the diazonium salts, followed by denitrogenation to form diazenium intermediate **AAAI**, that on intramolecular electrophilic cyclization (pathway) produced the indazole **85**.

### By Cadogan Reductive Cyclization/Phospha-Catalyzed Synthesis

**Scheme 84:**
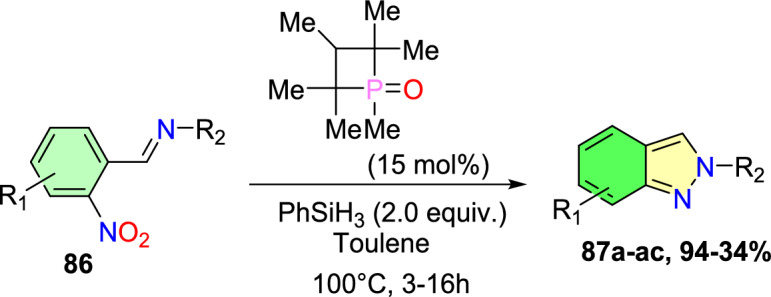
Cadogan synthesis of 2-aryl indazoles

Cadogan’s group established the method for reductive cyclization of nitro groups [105]. The technique was convenient for attaining the nitrogen-containing five-membered rings, such as carbazole motifs, as presented in Scheme 84. With its ability to function in both electron-rich and electron-poor systems, this ring closure procedure has the advantage of not being impacted by the substrate’s electronic state.

Nykaza et al. described [106] the Cadogan synthesis of indazoles from *o*-nitrobenzaldimines/*o*-nitroazobenzenes with 1,2,2,3,4,4-hexamethyl-phosphetane (small-ring phospha cycle) by *N*-*N* bond-formation. Dissimilar to earlier techniques that employed transition metal catalysis, stoichiometric reagent chemistry, or other high-energy azide substrates, this technique offers a simple, valuable, and straightforward phospha-catalytic process for N−N bond-forming mode (Scheme 84). Prior research on P^III^/P^V^=O redox cycling has primarily concentrated on the ring strain theories supporting silane reductants’ catalytic turnover of phosphine oxides. However, this method has progressed through a prevailing electronic component to the whole biphilic function of the phosphetane catalyst.

Table 2 details the scope of this catalytic transformation. With respect to N substitution, aliphatic substituents are well tolerated (Table 2A). For instance, the *o*-nitrobenzaldimines/*o*-nitroazobenzenes having alkyl substitutions such as 1°, 3° alkyl, cyclohexyl, 1-methyl piperidine, benzyl, and furan-2-methenyl units on basic nitrogen afforded the indazole products **87a–g** in good to excellent yields expediently. Likewise, various *N*-aryl-substituted substrates (**86**) also show good compatibility with Cadogan synthesis of indazoles **87h–s** (Table 2B) and afforded the products in excellent yields. *N*-aryl-substituted substrates (**86**) with unsubstituted phenyl afforded the indazole **87h** in 93% yield. The aromatic substrates with diverse functionalities on the phenyl ring, such as halogens (−I, −F), produced the indazole products **87j** and **87p**) in good yields. Substrates (**86**) with electron-rich (−OMe), electron-poor (−CN, −diCF_3_), and unsaturated (**87k**), units all undergo smooth catalytic cyclization and afford the indazole scaffolds **87i**, **87m**, **87n**, and **87k** in 73–86% yields. Further, poly-heterocyclic indazole products **87o** and **87s** can be prepared in excellent yields. Free −OH moieties do not inhibit deoxygenative hetero-cyclization (**87r**), while such substrates might undergo in situ silylation by the PhSiH_3_ reductant; desilylative workup ensured the recovery of the free OH group.

**Table 2:**
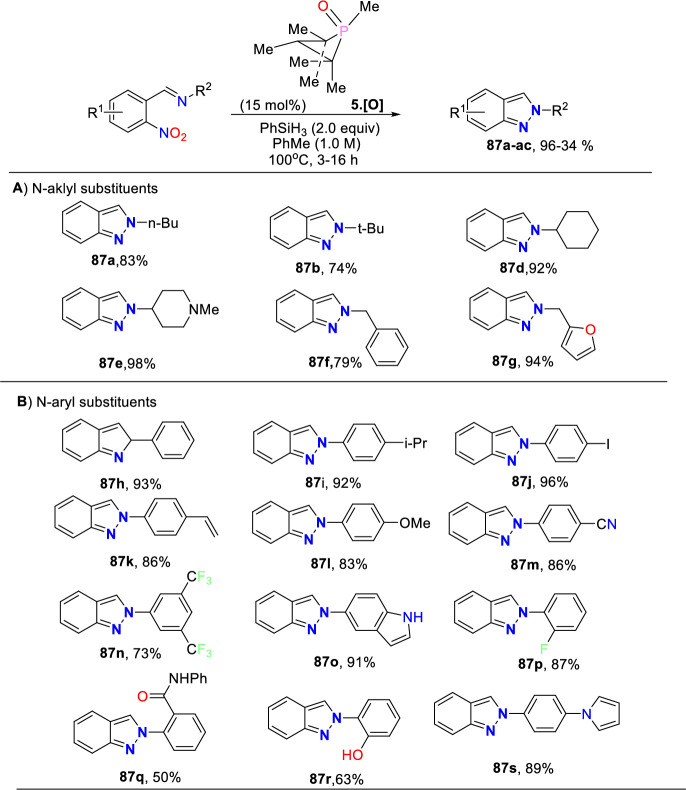

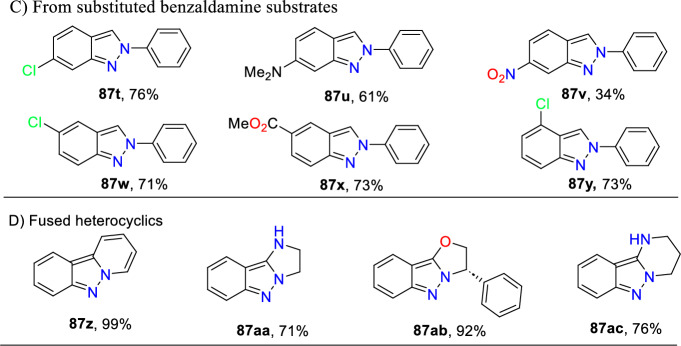
Substrate scope for catalytic Cadagon synthesis of 2-aryl indazoles

Similarly, the bezaldamines with –Cl, −NMe_2_, −NO_2_, and –COOMe units afforded the target indazoles **87t–y** in 34–76% yield (Table 2C). Unsurprisingly, the substrates with multiple nitro units were challenging for this strategy (Table 2C, **87v**); obviously, the catalyst does not selectively recognize the *o*-imino nitro motif. Likewise, the reaction was also amenable to non-benzaldimine substrates, enabling the construction of a variety of heterocyclic motifs through catalytic N−N bond formation reactions (Table 2D). For instance, deoxygenative hetero-cyclization of 2-(2-pyridyl)nitrobenzene delivers the fused poly-heterocyclic pyrido-[1,2-b]indazole (**87z**) in extraordinary yield within 4 h under standard catalytic conditions. Consecutively, indazolodihydroimidazoles (**87aa**), -dihydrooxazoles (**87ab**), and -tetrahydropyrimidines (**87ac**) are accessible in good yields at standard conditions. Stereochemistry adjacent to the reaction centers was retained upon cyclization (**87ab**).

### CH_3_OK (Base)-Promoted Synthesis

**Scheme 85:**
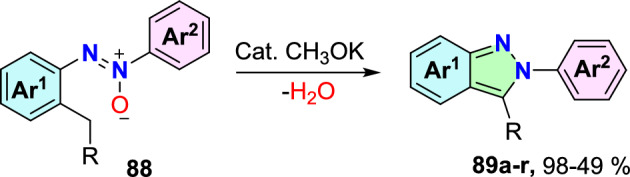
Potassium methoxide-promoted synthesis of 2-aryl-2*H*-indazoles

Zhou and coworkers described an easy and capable base-promoted benzyl C–H deprotonation and cyclization process to construct various 2-aryl-2*H*-indazoles starting from azoxybenzenes with *ortho*-alkyl substitutions (Scheme 85) [107]. In contrast to the transition metal-catalyzed strategies, this green protocol utilized a low-cost CH_3_OK base that eliminated the need for transition metal catalysts and oxidants. According to this method, a strong base would generate *ortho*-alkyl-substituted azoxybenzenes to undergo nucleophilic cyclization, resulting in the production of a series of 2-aryl-2*H*-indazoles and the exclusion of H_2_O. Outstandingly, the synthetic importance and practical capability of this strategy were demonstrated by the construction of numerous fluorescent, bioactive molecules and gram-scale reactions.

**Scheme 86:**
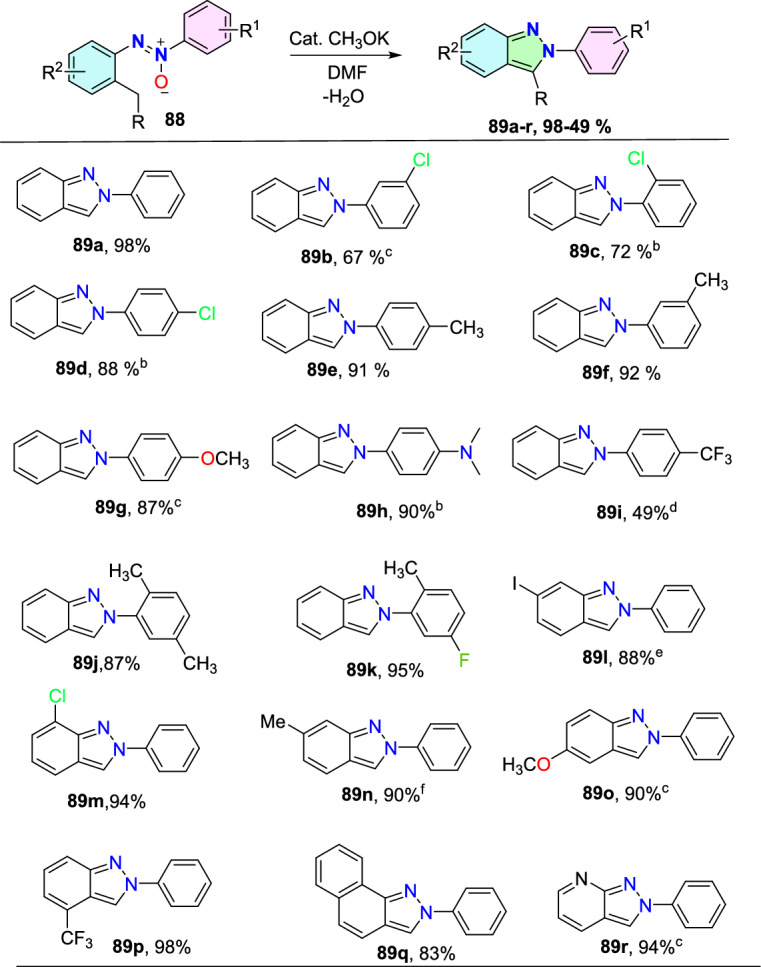
Substrate scope for 2-aryl-2*H*-indazoles

First, the optimization was performed with (*Z*)-1-phenyl-2-(*o*-tolyl)diazene 1-oxide (**88a**) as a model substrate to assess the practicability of direct nucleophilic cyclization to get the 2-phenyl-2*H*-indazole (**89a**). Encouragingly, the target indazole **89a** was obtained in 98% yield under the ideal reaction conditions of 0.2 equiv ^*t*^BuOK in DMF at 90 °C under an argon atmosphere for about 8 h. Next, the substrate scope of the reported intramolecular cyclization was explored (Scheme 86). The second aromatic ring (R^1^) of *ortho*-alkyl substituted azoxybenzenes with diverse substituents such as halogens (2-Cl, 3-Cl, 4-Cl), electron-neutral (4-Me, 3-Me), electron-donating units (4-OMe, 4-NMe_2_), or an electron-withdrawing (4-CF_3_) unit were well tolerated at dissimilar positions on the phenyl ring to afford the products **89b–j** in superior yields. The effect of steric hindrance on the efficacy of this transformation was low, evident from the generation of indazole products **89j** and **89k** in 87% and 90% yields, respectively. The investigation of the effect of diverse substituents on the first aromatic ring (R^2^) showed that R^2^ substituents such as halogens (−I, −Cl), electron-donating −CH_3_ and −OMe, and electron-withdrawing –CF_3_ afforded the respective indazoles **89l–p** in 98–88% yield. Fascinatingly, a fused ring (naphthyl) or a heterocycle was compatible with this reaction to furnish the relevant indazole motifs **89q** and **89r** in excellent yield. Higher CH_3_OK loading or higher temperature was required for complete conversion in some cases.

**Scheme 87:**
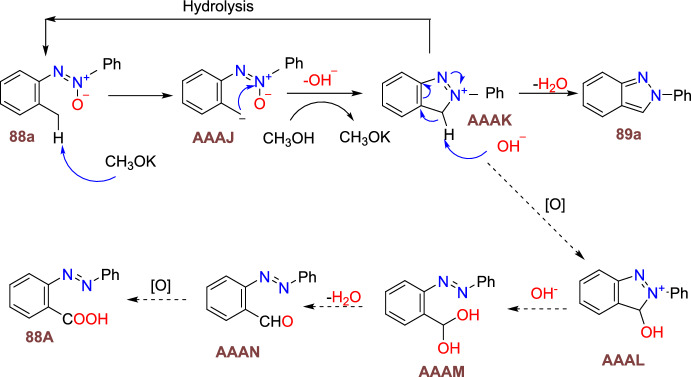
Plausible mechanism for 2-phenyl-2*H*-indazole **89a**

A plausible mechanism for the reported CH_3_OK-catalyzed cyclization of (*Z*)-1-phenyl-2-(*o*-tolyl)diazene 1-oxide (**88a**) is depicted in Scheme 87. Primarily, CH_3_OK deprotonates **88a** to generate carbanion intermediate **AAAJ** that, on intramolecular nucleophilic cyclization, produces the intermediate **AAAK**, followed by dehydration and isomerization processes to develop the intended product **89a**. Conspicuously, intermediate **AAAK** hydrolyzes simply to starting material **88a**, indicating that the reaction was water-sensitive. Apart from that, **AAAK** was oxidized to **AAAL** by the oxidant. Afterwards, the attack of hydroxyl ion (nucleophile) towards **AAAL** generated the acetal **AAAM** that transformed into **AAAN** by dehydration. Finally, the intermediate **AAAN** on further oxidation generated the target product **88A**.

### By conventional methods

#### Hydroxyl Acetophenone

**Scheme 88:**
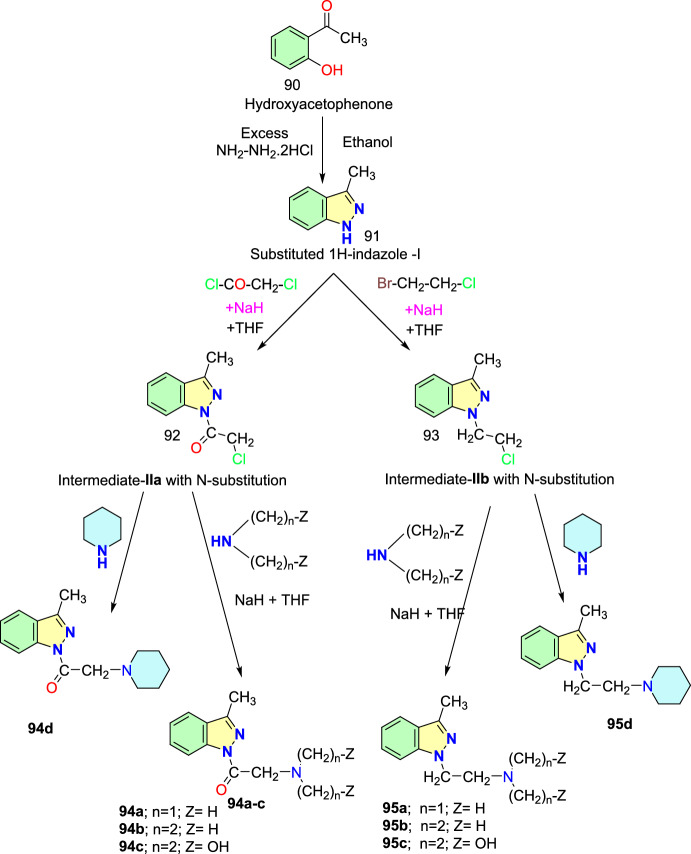
Reaction pathway for the synthesis of indazole derivatives **1a–d** and **2a–d**

Farrukh’s group described a simple and economical synthesis of 3-methyl-1*H*-indazoles derivatives (**94a–d** and **95a–d**) in three-step reactions and evaluated their antibacterial activity [108] (Scheme 88).

**Scheme 89:**
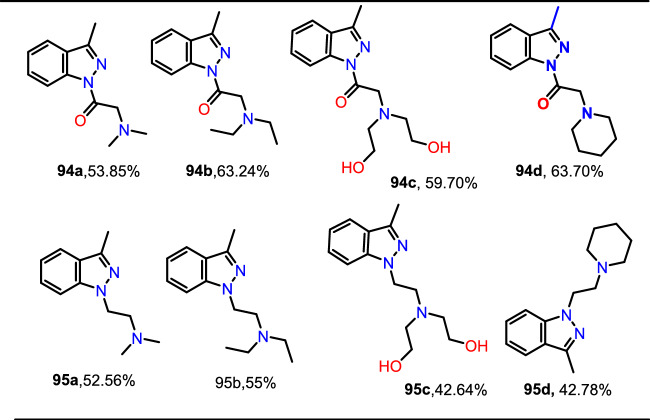
Indazole derivatives **94a–d** and **95a–d**

Initially, *o*-hydroxy acetophenone reacts with an excess amount of hydrazine dihydrochloride in ethanol to afford 3-methyl-1*H*-indazole. Next, the 3-methyl-1*H*-indazole was combined with either chloroacetyl chloride or 1-bromo-2-chloroethane in the presence of NaOH and tetrahydrofuran to form the two consequent intermediates 1-(2-chloroethan-1-oyl)-3-methyl-1*H*-indazole and 1-(2-chloroethyl)-3-methyl-1*H*-indazole, respectively. Finally, in the third step, the generated two intermediates were treated separately with dimethyl amine, diethyl amine, diethanolamine, or piperidine to afford the final indazole derivatives **94a–d** and **95a–d** in superior yields (Scheme 89).

#### Using 2-Chloro Benzonitrile and Aryl Hydrazines

**Scheme 90:**
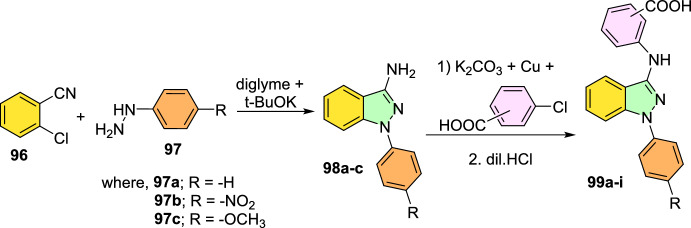
Reaction path for the synthesis of different 1,3-substituted 1*H*-indazoles

Kumar’s group prepared the novel indazole compounds by three-step reactions [109] starting from the reaction of 2-chloro benzonitrile (**96**) with phenyl/aryl hydrazine (**97**) in the presence of catalyst potassium ^*t*^butoxide(t-BuOK) and the solvent diglyme to produce substituted 3-amino-1-phenyl-1*H*-indazole derivates (**98**). Next, C–N coupling takes place between the amino group of the intermediate **(98)** and *o*-chloro benzoic acid using dry K_2_CO_3_ as a base and Cu powder as a metal catalyst in DMF to form the consequent intermediate, *N*,1-diaryl-1*H*-indazol-3-amines (**99a–i**) as presented in Scheme 90.

**Scheme 91:**
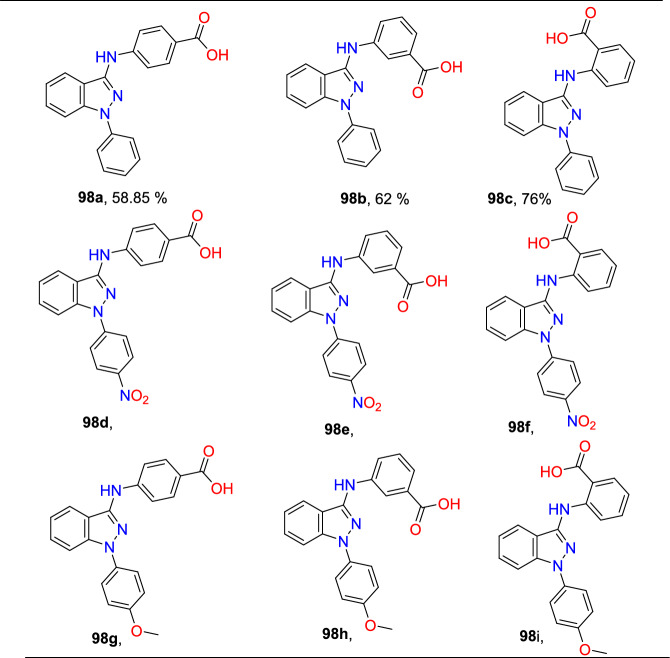
Synthesis of *N*,1-diaryl-1*H*-indazol-3-amines

The authors described the preparation of a sequence of 1,3-substituted 1*H*-indazole motifs **98a–i** that is shown in Scheme 91. First, in the first step, various 3-amino-1-aryl-1*H*-indazole derivates **98a–c** were prepared by the nucleophilic addition on nitrile of 2-chloro benzonitrile (**96**) with phenylhydrazine (**97a**), 4-nitro phenylhydrazine (**97b**), and 4-methoxyphenyl hydrazine (**97c**). Next, the phenylhydrazine (**97a**) on deprotonation with *t−*BuOK followed by nucleophilic addition with nitrile of 2-chloro benzonitrile (**96**) afforded the 1-phenyl-1*H*-indazol-3-amine (**98a**) in 77.85% yield. Next, the presence of base (K_2_CO_3_) indazole **98a** on Cu promoted C–N coupling with 4-Cl, 3-Cl, and 2-Cl benzoic acids consecutively to afford the respective 1-phenyl-1*H*-indazol-3-yl)amino benzoic acids **98a–c** in good yields. Further, the 1-(4-nitrophenyl)-1*H*-indazol-3-amine (**98b**) on Cu promoted C–N coupling with 4-Cl, 3-Cl, and 2-Cl benzoic acids in the presence of K_2_CO_3_, producing the respective *N*-(nitrophenyl)-1-phenyl-1*H*-indazol-3-amine motifs **99d**, **99e**, and **99f.** Moreover, the 1-(4-methoxyphenyl)-1*H*-indazol-3-amine (**98c**) on similar reaction with 4-Cl, 3-Cl, and 2-Cl benzoic acids afforded respective N-(methoxyphenyl)-1-phenyl-1*H*-indazol-3-amines **99g–99i** (Scheme 91).

**Scheme 92:**
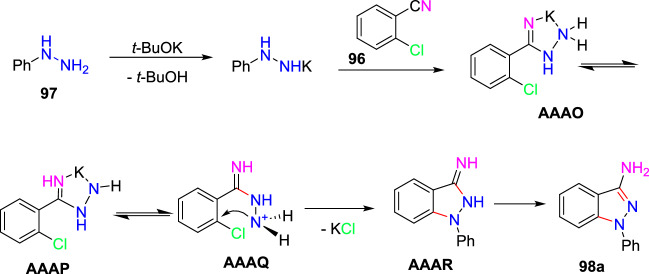
Possible mechanism for 1-phenyl-1*H*-indazol-3-amine (**98a**)

The predicted mechanism for the first step of synthesis of the reaction towards amino indazole product (**98a**) is presented in Scheme 92. Initially, the ^*t−*^BuOK treatment of hydrazine (**97**) directly generated the active PhNHNHK species, which, on nucleophilic addition with the nitrile of 2-chloro benzonitrile (**96**), formed the intermediate **AAAO** that produced the intermediate **AAAP** through a reversible 1,4 *H*-shift of species **AAAO.** Next, the intermediate **AAAP** is transformed to intermediate AAAQ upon subsequent isomerization. The intermediate **AAAQ** on subsequent nucleophilic substitution formed the intermediate **AAAR** that on aromatization, afforded the required free aminoindazole product **98a**.

#### Green approach

**Scheme 93:**
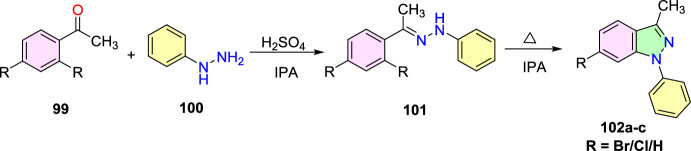
Synthetic pathway for 3-methyl-1-phenyl-1*H*-indazole motifs (**102a–c**)

Susmitha’s group reported a simple, efficient, and economically viable synthesis of indazoles and their derivatives (**102a–c**) by a novel green synthetic protocol involving aryl hydrazones as an intermediate (Scheme 93) [110]. The sequence involves C–N bond formation through simple ipso substitution instead of a metal-promoted cross-coupling reaction. This method is green and atom-economic, and maintains mild conditions throughout the experiment.

Initially, various 1-(2,4-disubstituted)-phenyl-ethylidene-2-phenylhydrazone intermediates (**101a–c**) were acquired by the reaction of phenylhydrazine (**100**) with 2,4-dibromo, 2,4-dichloro, and 2-fluoro acetophenones, respectively. These intermediates on metal-free C–N bond formation through simple *ipso* substitution produced the indazole products **102a–c** in good yields. The results of the optimization reaction conditions were interesting for chemists working on 1-arylethylidene-2-phenylhydrazine and 3-methyl-1-phenyl-1*H*-indazoles. Protic solvents were readily available for this protocol. Different solvents utilized for the construction of 1-(2,4-dibromophenyl)ethylidene-2-phenylhydrazine (**101a**) and 1-(2,4dichlorophenyl)ethylidene-2-phenylhydrazine **101b** at constant temperature are presented in Table 3. The experimental results revealed that isopropyl alcohol, one of the three solvents used, typically yields better results than methanol and ethanol.

**Table 3:**
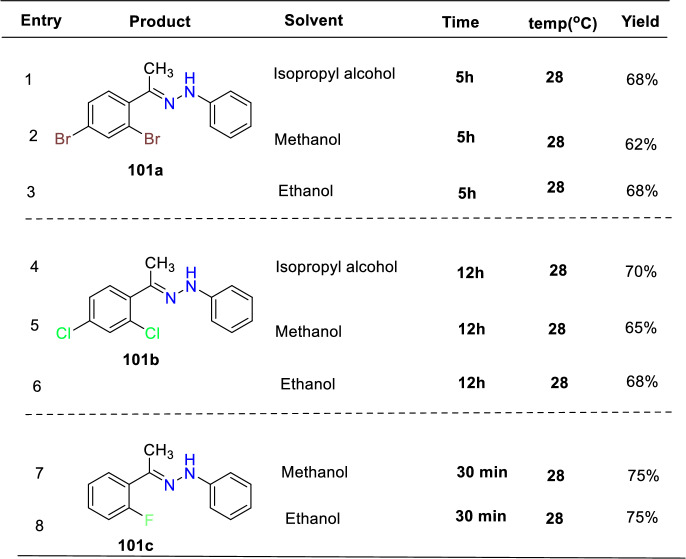
Optimized reaction conditions for the synthesis of **3a**, **3b**, and **3c**

Table 4 shows that the compound 1-(2-fluorobenzylidene)-2-phenylhydrazine (**101c**) obtained potential outcomes in both ethanol and methanol solvents. The yield of the products 6-bromo-3-methyl-1-phenyl-1Hindazole (**102a**) and 6- chloro-3-methyl-1-phenyl-1*H*-indazole (**102b**) was obtained in high percentage using isopropyl alcohol as solvent compared to methanol and ethanol. For the synthesis of 1-phenyl-1*H*-indazole (**102c**), both ethanol and methanol gave similar yields.

**Table 4:**
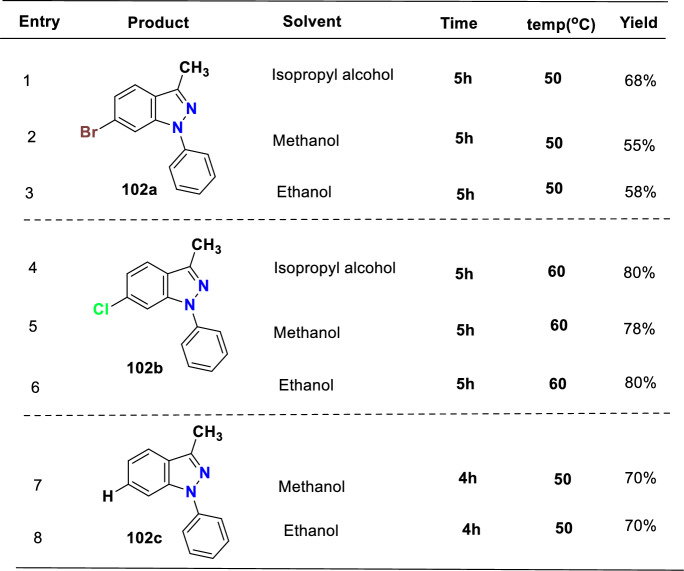
Optimized reaction conditions for the synthesis of **4a**, **4b**, and **4c**

Instead of utilizing an expensive metal-catalyzed cross-coupling reaction for indazole synthesis, this method provided a more eco-friendly, practical, and effective *ipso*-substitution method beginning with readily available starting materials under a mild environment throughout the experiment.

## Conclusion

The broad range of pharmacological activities exhibited by indazole derivatives makes them indispensable in the field of medicinal chemistry. Many vital bioactive molecules that are important in the pharmaceutical industry have been synthesized using this motif. These scaffolds can be combined with many bioactive motifs to design potential drug candidates. This review covered the literature on the biological significance of some existing indazole drugs and the biological properties of a few indazole moieties under clinical trials, with particular attention to the latest synthetic strategies such as metal-catalyzed, metal-free, ultrasound- and microwave-assisted, and conventional methods reported from 2017 to the present (2024). The current review described the progression of novel ideas and synthetic strategies in addition to traditional techniques for the production of different novel indazole derivatives. Therefore, we believe that this review will inspire research scientists and chemists to create new procedures for constructing and assessing the pharmacological characteristics of innovative molecules with indazole motifs that exhibit superior performance.

## Data Availability

No datasets were generated or analyzed during the current study.

## Abbreviations

NSAID: Non-steroidal anti-inflammatory drug
NTRK: Neurotrophic tyrosine receptor kinase
RCC: Renal cell carcinoma
ROS: Reactive oxygen species
ALK: Anaplastic lymphoma kinase
HIV: Human immunodeficiency virus
NRTI: Nucleoside reverse transcriptase inhibitor
SAR: Structure–activity relationship
Dppf: 1,1′-Bis(diphenylphosphino)ferrocene
NaO^t^Bu: Sodium tertiary butoxide
TBAF: Tetra-n-butylammonium fluoride
DCE: 1,2-Dichloroethane
DFT: Density functional theory
TFE: 2,2,2-Trifluoroethanol
DMEDA: *N*,*N*′-Dimethylethylenediamine
*p*-ABSA: 4-Acetamidobenzenesulfonyl azide
DBN: 1,5-Diazabicyclo[4,3,0]non-5-ene
DMSO: Dimethylsulfoxide
PEG: Polyethylene glycol
MW: Microwave
DMF: Dimethylformamide
Phen: 1,10-Phenanthroline
KOCN: Potassium cyanate
TFA: Trifluoroacetic acid
H_3_PMo_12_O_40_: Phosphomolybdic acid
H_3_PW_12_O_40_: Phosphotungstic acid
US: Ultrasound
P(OEt)_3_: Triethyl phosphate
CDSCS: Copper-doped silica cuprous sulphate
TBAA: Tetrabutylammonium azide
PhSiH_3_: Phenylsilane
IPA: Isopropyl alcohol
